# Comparative cranial biomechanics reveal macroevolutionary trends in theropod dinosaurs, with emphasis on Tyrannosauroidea

**DOI:** 10.1002/ar.70126

**Published:** 2026-01-27

**Authors:** Evan Johnson‐Ransom, Paul Gignac, Daniel E. Barta, Ryan N. Felice, Eric Snively

**Affiliations:** ^1^ Department of Organismal Biology University of Chicago Chicago Illinois USA; ^2^ University of Arizona Health Sciences Tucson Arizona USA; ^3^ College of Osteopathic Medicine at the Cherokee Nation Oklahoma State University Tahlequah Oklahoma USA; ^4^ Centre for Integrative Anatomy, Cell and Developmental Biology University College London London UK; ^5^ Research Department of Genetics, Evolution, and Environment University College London London UK

**Keywords:** biomechanics, feeding, phylogeny, Theropoda, Tyrannosauroidea

## Abstract

*Tyrannosaurus* is viewed as a model organism in vertebrate paleontology, with numerous studies analyzing its feeding biomechanics. Nonetheless, the evolution of this feeding performance has been under‐addressed in Tyrannosauroidea, especially in basal tyrannosauroids. Here we used muscle‐force reconstruction and finite element analysis (FEA) to quantify the cranial performance of tyrannosauroids and outgroup theropod clades. 2D (planar) cranial models, set to standardized skull lengths and jaw adductor forces, were used to analyze the evolution of feeding behavior in a large sample size of Tyrannosauroidea and other theropods. Sampled stresses matched well between planar and 3D analyses of three disparately shaped crania, suggesting valid interpretations from 2D models along the lateral sides of theropod crania if symmetrical bite loadings are assumed. We traced cranial evolution by sampling stresses at homologous points of theropod crania and input their average stress values into a maximum likelihood ancestral character state estimation. Our results show tyrannosauroids having moderate‐to‐low cranial stresses. We further tested whether the average stress value correlates with head size through phylogenetic generalized least square regressions. We found that 2D FEA provides significant information on the evolution of feeding performance in a major dinosaur clade. Along internal branches of Tyrannosauroidea, hypothetical common ancestors exhibit low cranial stress values owing to a combination of robust skulls and cranial protuberances. These traits may have been passed down to later tyrannosauroids, enabling them to handle high forces. Our results additionally demonstrate a possible correlation between cranial shape (brevirostrine versus longirostrine) and inferred cranial performance in non‐tyrannosauroid clades.

## 1 INTRODUCTION

Predators have evolved diverse morphological features that allow them to dispatch prey. For example, mammalian carnivores possess heterodont teeth capable of puncturing, holding, slicing, and cracking prey tissues (Wroe et al., 2005; Lanszki et al., 2020). The jaws of some large crocodylians are lined with rows of conical teeth used to secure especially large‐bodied prey for drowning (Gignac & Erickson, 2016; O'Brien et al., 2019). Even edentulous birds predate by pinning and puncturing prey with talons and recurved beaks (Fowler et al., 2009; Snively & Russell, 2007). Through niche divergence, prey resources are subdivided across a food web based on adaptation and exclusion (Hardin, 1960; Lyson & Longrich, 2010; Van Valkenburgh, 1985), relating to predator sizes, morphologies, or behaviors. Often, the largest carnivores become apex predators, feeding upon larger prey, while medium‐sized and smaller predators typically prey upon animals within their own, or smaller, body size ranges (Van Valkenburgh & Molnar, 2002). By comparing body sizes, jaw morphologies, feeding behaviors, and ecologies in extant vertebrates, we can predict how predatory roles of extinct ones, such as large theropod dinosaurs, may have been partitioned (Calandra et al., 2008; Keddy, 1989; Lehman, 1987).

Theropods are a clade spanning from the Late Triassic Period, as plesiomorphically carnivorous dinosaurs, to today as birds. Carnivorous Mesozoic theropods came in a variety of sizes and possessed diverse craniomandibular and dental morphologies, ranging from the short‐faced abelisaurids, the crocodile‐snouted spinosaurids, and the deep‐snouted tyrannosaurids (Johnson‐Ransom, 2021; Johnson‐Ransom et al., 2024; Rowe & Rayfield, 2025). The goal of the current study is to examine predatory niche occupations among theropods through the lenses of cranial biomechanics and feeding capabilities. We achieve this aim by leveraging muscle‐force reconstructions and finite element analysis (FEA) to study cranial form and function in theropods. We focus on the theropod *Tyrannosaurus rex* (Osborn 1905) and its kin within the clade Tyrannosauroidea. Tyrannosauroidea represents a valuable model group because the clade consists of numerous well‐preserved taxa with a range of craniodental morphologies and has been subject to ongoing phylogenetic characterization (Brusatte & Carr, 2016; Voris et al., 2025). For example, the clade includes small, early tyrannosauroids with gracile crania, flattened bladelike teeth, and relatively long, three‐clawed forelimbs (e.g., *Guanlong* and *Dilong*) as well as large derived tyrannosauroids with deeply robust crania, widely expanded teeth, and reduced forelimbs (e.g., *Tyrannosaurus*). Tyrannosauroids of various body sizes and phylogenetic positions allow for understanding functional adaptations with respect to extreme feeding capabilities among extinct predators (Bates & Falkingham, 2012; Brusatte, Norell, et al., 2010; Gignac & Erickson, 2017; Johnson‐Ransom et al., 2024; Rayfield, 2007). Functional analyses indicate that adult *T. rex* had reinforced skulls able to resist high feeding loads, equipped with immobile cranial joints and fused nasals that strengthened the rostrum for biting, alongside powerful neck muscles that assisted in the removal of flesh from carcasses during feeding (Cost et al., 2020; Molnar, 1991; Rayfield, 2004; Snively et al., 2006; Snively & Russell, 2007). Notably, jaw adductor system models indicate that an adult *Tyrannosaurus* was capable of exerting a high bite force in the range of 18,014 to 64,000 N, enabled by robust teeth, stout jaws, and hypertrophied jaw muscles (Bates & Falkingham, 2012; Cost et al., 2020; Erickson & Olson, 1996; Gignac & Erickson, 2017). Trace fossils from *T. rex* teeth, along with the analyses of skull and neck biomechanics, support *T. rex* using the “puncture‐pull” method of feeding. Puncture‐pull feeding is a process whereby a predator bites into its prey, retracts its head, and thus drags its teeth through and across the soft and hard tissues of the prey (Erickson & Olson, 1996; Rayfield, 2004), removing large sections of tissue.

While there is a wealth of data on feeding in *Tyrannosaurus*, there is considerably less understanding of the feeding behaviors of its immediate relatives within the clade Tyrannosauroidea. This group showed a remarkable range of body sizes and craniodental phenotypes, which track the expansion of the feeding system present in *Tyrannosaurus* from its early evolutionary origins. Among tyrannosauroids, Proceratosauridae was an early diverging clade consisting of small and large theropods. These included diminutive species with pneumatized crests, from Asia and Europe of the Middle‐Jurassic and Early Cretaceous periods (Rauhut et al., 2009; Xu et al., 2006) as well as large‐bodied taxa like *Yutyrannus* and *Sinotyrannus* (Ji et al., 2009; Xu et al., 2012). Pantyrannosauria were non‐proceratosaurid tyrannosauroids whose early representatives, such as *Dilong*, *Moros*, and *Suskityrannus*, possessed relatively gracile crania (Nesbitt et al., 2019; Xu et al., 2004; Zanno et al., 2019). Most of the small, early tyrannosauroids possessed relatively small jaws and thin teeth (e.g., *Dilong* and *Proceratosaurus*), implying that these animals lacked the feeding phenotypes necessary for dispatching large and robust prey. Instead, it is thought that these early tyrannosauroids fed exclusively on small prey (Brusatte & Carr, 2016; Brusatte, Norell, et al., 2010; Rauhut et al., 2009; Xu et al., 2004). These forms may have been similar to juvenile tyrannosaurids such as specimens of *Raptorex* and *Tarbosaurus*, which also lacked robust jaws and teeth (Johnson‐Ransom et al., 2024; Rowe & Rayfield, 2024; Rowe & Snively, 2021). Initially, early adult tyrannosauroids lived alongside larger predatory dinosaurs, including allosauroids; however, upon the extinction of allosauroid theropods in the Northern Hemisphere, coelurosaurian theropods (e.g., dromaeosaurids and tyrannosauroids) and ceratosaurs (e.g., abelisaurids) exploited the vacancy (Brusatte & Carr, 2016; Loeuff & Buffetaut, 1991; Tortosa et al., 2014; Zanno et al., 2019). Abelisaurids such as *Tarascosaurus* and *Arcovenator* were the predominant predators of the Late Cretaceous Period of Europe (Loeuff & Buffetaut, 1991; Tortosa et al., 2014). The Late Cretaceous European archipelago currently lacks tyrannosauroid fossils. Tyrannosauroids, specifically tyrannosaurids (e.g., *Gorgosaurus*, *Tarbosaurus*, and *Tyrannosaurus*), became the predominant predators across Asiamerica (Brusatte, Norell, et al., 2010; Rowe & Rayfield, 2024, 2025; Rowe & Snively, 2021; Zanno et al., 2019; Zanno & Makovicky, 2013). Adult members of Tyrannosauridae had massive skulls and robust teeth reinforced for forceful biting. Exemplars included taxa such as *Gorgosaurus*, *Tarbosaurus*, *Daspletosaurus*, and *Tyrannosaurus* (Hone & Tanke, 2015; Hurum & Sabath, 2003; Therrien et al., 2021).

Although most tyrannosaurids possessed robust skulls, this pattern was contrasted by alioramins, such as *Alioramus* and *Qianzhousaurus*, which had relatively gracile crania. Alioramins may not have engaged in forceful biting, in contrast to their deep‐snouted relatives (Foster et al., 2022; Lü et al., 2014; Rowe & Rayfield, 2024). Nonetheless, as tyrannosauroid body size increased, trends in the evolution of the head skeleton may have had important contributions to this group's overall transition from small‐bodied, generalized carnivores to large‐bodied apex predators (Brusatte & Carr, 2016; Foth & Rauhut, 2013; Johnson‐Ransom et al., 2024) capable of extreme osteophagy (Gignac & Erickson, 2017), the ability to splinter and consume bone.

We used finite element analysis (FEA), a non‐invasive quantitative technique that models a complex structure's responses to forces, in evaluating stress and strain levels (Bright, 2014; Rayfield, 2007). Here we introduce FEA conceptually to link its results to feeding biology, and detail our application of FEA in the Methods. FEA elucidates structural performance in skulls with inferences made to feeding behavior (Johnson‐Ransom et al., 2024; Rayfield, 2007; Rowe & Snively, 2021). Finite element analysis allows us to evaluate the cranial structural performance of tyrannosauroids and other theropods, and time‐scaled phylogenies elucidate macroevolutionary patterns, such as those in theropod feeding functional morphology, by exploiting large sample sizes (Johnson‐Ransom, 2021; Ma et al., 2022). To capture a sizeable phylogenetic sample, we applied 2D models to compare and evaluate the cranial stress magnitudes of tyrannosauroids with non‐tyrannosauroid theropods, similar to previous 2D FEA analyses (Ma et al., 2022; Rayfield, 2011; Rowe & Snively, 2021). For example, Rayfield (2011) evaluated the structural performance of megalosauroid and allosauroid theropod crania, with larger theropods (*Carcharodontosaurus* and *Spinosaurus*) exhibiting higher cranial stress than smaller theropods (*Afrovenator*). Ma et al. (2022) examined the mechanical performance of carnivorous and herbivorous coelurosaurian theropod mandibles in an evolutionary context. The authors (Ma et al., 2022) reconstructed the jaw muscles and positioned them at specific angles and applied alligator bone properties to the theropod mandibles (Zapata et al., 2010). Coelurosaurian theropods with robust mandibles (oviraptorosaurs and tyrannosaurids) exhibited lower stress and a higher bite force than coelurosaurian theropods with gracile mandibles (dromaeosaurs and ornithomimosaurs), and relative mandibular strength increased through tyrannosaurid ontogeny (*Tyrannosaurus* and *Tarbosaurus*). Rowe and Snively (2021) similarly applied planar FEA to mandibles of diverse large theropods in comparison with a growth series of *Tyrannosaurus* specimens, validated against 3D models from independent analyses (Mazzetta et al., 2009; Rowe & Snively, 2021). Building on these efforts, our current study offers a broad comparison of the functional feeding performance of coelurosaurian (tyrannosauroids) and non‐coelurosaurian theropods (e.g., allosauroids, megalosauroids, and ceratosaurs). It evaluates the cranial performance of *Tyrannosaurus* at different ontogenetic stages with similarly sized tyrannosauroids and non‐tyrannosauroid theropods.

While 3D models provide volumetric geometry of the theropod specimens, 2D models enable a larger, more inclusive phylogenetic comparison with the benefit of suggesting hypotheses for further investigation through 3D methods. 2D and 3D models of biological objects are not necessarily comparable, as 2D models are limited to a specific plane (lateral view) and 3D models capture the full geometry of a biological object; Rayfield (2011) addressed limitations and caveats with 2D FEA. Most crucially, 2D analyses are misleading about stress and strain in structures that are asymmetrical and/or curved (Smith et al., 2023), especially with asymmetrical and torsion‐inducing loading regimes (Smith et al., 2023).

As a testament to this, results for 2D and 3D stress distribution (regional relative magnitudes) have been more similar for relatively planar structures and symmetrical loadings, such as mandibles of *Dunkleosteus* and other arthrodires, and some theropod dinosaurs (Rowe & Snively, 2021; Snively et al., 2010). Critically, theropod adductor musculature (Cost et al., 2020; Gignac & Erickson, 2017; Rowe & Snively, 2021) had minor medial or lateral components relative to the sagittal plane of the mandibular rami, which differs from the torsional loading and induced strain regimes in primates and ungulates (Smith et al., 2023). Thus, for the current study, interpretations of 2D results are necessarily limited to relative stress magnitudes on the lateral surface of the crania, with the absolute fidelity of such results unknown.

We tested the 2D analyses against 3D model results from a previous study (Johnson‐Ransom, 2021; Johnson‐Ransom et al., 2024) to assess how informative the 2D results would be. Assessing correspondence between 2D and 3D results required: (1) comparison of relative stress magnitudes at sampled locations, (2) consideration of biological differences between 2‐ and 3D models, and (3) caution against overinterpreting 2D results. The match between 2D and 3D results can inform the complexity of hypotheses testable by the 2D models. We find that stress distributions match sufficiently for provisional confidence in the 2D procedures (see section 4) for testing circumscribed hypotheses of structural performance in theropod crania, and for raising further comparative hypotheses about their feeding function. Additionally, some tyrannosauroids have ornamented skulls, which may alter stress–strain distributions. For example, biomechanical simulations have presented contrasting results where cranial crests may have resisted high loadings (Johnson‐Ransom et al., 2024; Rayfield, 2011) or were structurally weak during feeding (Xing et al., 2015). To evaluate the influence of cranial ornamentation on cranial stress performance of theropods, we reconstructed crestless alternates of *Dilophosaurus* and *Guanlong* to interpret the level of stress present, including for comparison to other, naturally crestless theropods.

## 2 HYPOTHESES

The following main hypotheses were tested by simulating and comparing the levels of stress and strain present in the 2D theropod cranium models.

Hypothesis 1 Based on the structural mechanics of slender structures, the crania of gracile‐snouted and/or small‐bodied tyrannosauroids (e.g., alioramins; small, early tyrannosauroids; and juvenile tyrannosaurines) will exhibit higher cranial stress than deep‐snouted tyrannosauroids when subject to standardized forces.

Hypothesis 2 Similar to Hypothesis 1, we expect a similar scenario with gracile‐snouted non‐tyrannosauroid theropods (spinosaurids, carcharodontosaurids, and dromaeosaurids) exhibiting higher cranial stress than deep‐snouted theropods (abelisaurids) when subjected to standardized forces.

Hypothesis 3 The crests of theropods such as *Guanlong* and *Dilophosaurus* acted as a buttress when the animals were biting, thus enabling crested forms to manage higher standardized forces.

Hypothesis 4 Phylogenetic analyses such as maximum likelihood and ancestral character state estimates will show that as tyrannosauroids evolved and diversified into larger and more robust forms, cranial stress magnitudes decreased relative to skull length, enabling them to resist increasing bite forces.

These hypotheses can inform plausible theropod niche occupation and divergence scenarios via interpretation of the cranial stress magnitudes and distribution in our 2D theropod models. Cranial performance and phylogenetics considered together will elucidate the evolution of feeding function and performance of theropod clades.

## 3 MATERIALS AND METHODS

### 3.1 Theropod specimens

Below we detail procedures for 2D FEA and those for 3D models that test for sufficient, interpretable realism of 2D results. For a large phylogenetic sample and comparison, we generated 2D models of tyrannosauroid and non‐tyrannosauroid theropod crania (Table S1, Supporting Information and Figure 1). While the crania of the theropod taxa were set to 1 m for the 2D finite element modeling, we recorded the actual lengths of the crania in the image processing software FIJI (Schindelin et al., 2012) (Table S1). The 2D models were derived from photos of skeletal mounts and scientific illustrations pertaining to the holotypes (e.g., *Majungasaurus* FMNH PR 2100) or exceptionally complete specimens (*Tyrannosaurus* AMNH FARB 5027) of the included taxa. Some of the theropod cranial models were composites with the missing materials filled in by comparative osteology with sister taxa, proximate outgroups, or individuals of varying body sizes (Table S1). Examples of composite taxa include *Deinonychus* (Scott Hartman), the composite megaraptorid (T. K. Robinson), and *Spinosaurus* (Sereno et al., 2023). The *Deinonychus* model was a composite of YPM 5232 and AMNH FARB 5210. The cranial bones of *Deinonychus* YPM 5232 include the right maxilla, nasals (left and right), premaxillae (right and left), jugals (left and right), right squamosals, postorbitals (left and right), right lacrimal, left palatine, and right quadratojugal (Ostrom, 1969). The cranial bones of *Deinonychus* AMNH FARB 5210 include the quadratojugal (left and right), squamosal (left and right), and left jugal (Paul, 1988). Because preserved megaraptoran skulls are incomplete, the composite megaraptorid cranium consisted of *Megaraptor* (nasals, premaxilla, maxilla, and frontals), *Murusraptor* (lacrimals, postorbitals, and braincase), *Orkoraptor* (quadrate), and proximate theropod outgroups. The *Spinosaurus* model was a composite of MSNM V4047 (rostrum), the neotype FSAC‐KK‐11888 (posterior nasals, nasal crest, lacrimals), and *Irritator* (braincase); the posterior cranial elements were scaled to match the proportions of the rostrum of MSNM V4047. In our results and discussion, we provide interpretations only for those bones that are known from the individuals that make up the composite specimens.

**FIGURE 1:**
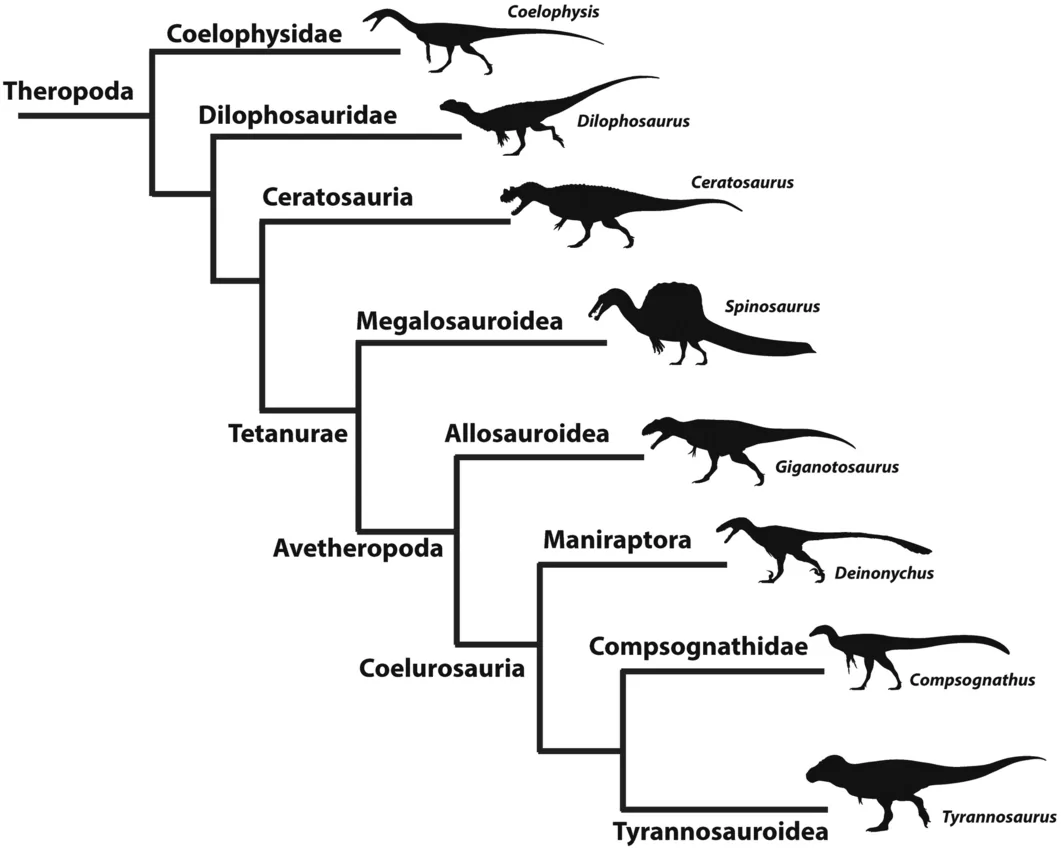
Cladogram of theropod groups used for the 2D FEA study. The cladogram was modified from Brusatte et al. (2014). Theropod silhouette images were from Phylopic. Artist credit: Scott Hartman (*Deinonychus*, *Giganotosaurus*, *Ceratosaurus*, and *Coelophysis*), Jagged Fang Designs (*Compsognathus*), Ivan Iofrida (*Spinosaurus*), Tasman Dixon (*Dilophosaurus*), and Jack Mayer Wood (*Tyrannosaurus*).

### 3.2 Museum abbreviations

AMNH FARB, American Museum of Natural History Fossil Amphibians, Reptiles, and Birds, New York City, USA; BHI, Black Hills Institute of Geological Research, Hill City, USA; BMRP, Burpee Museum Rockford Paleontology, Rockford, USA; BYU, Brigham Young University Museum of Paleontology, Provo, USA; CAGS, Chinese Academy of Geological Sciences, Beijing, China; CMN, Canadian Museum of Nature, Ottawa, Canada; CV, Municipal Museum of Chongqing, Chongqing, China; DINO, Dinosaur National Monument, Vernal, USA; ELDM, Erlianhaote Dinosaur Museum, Erenhot, China; FMNH, Field Museum of Natural History, Chicago, USA; FSAC, Aïn Chock Faculty of Sciences in Casablanca, Casablanca, Morocco; GM, Ganzhou Museum, Ganzhou City, China; IGM, Institute of Geology, Mongolian Academy of Sciences, Ulaanbaatar, Mongolia; IVPP, Institute of Vertebrate Paleontology and Paleoanthropology, Beijing, China; JMP, Jinzhou Museum of Paleontology, Jinzhou, China; LH PV, Long Hao Institute of Geology and Paleontology, Hohhot, China; MCF, Carmen Funes Municipal Museum, Plaza Huincul, Argentina; MC, Carlos Ameghino Provincial Museum, Cipolletti, Argentina; MPEF, Paleontological Museum of “Egidio Feruglio,” Chubut, Argentina; MUCP, Museum of Natural Sciences, National University of Comahue, Comahue, Argentina; MPCA, Carlos Ameghino Provincial Museum, Cipolletti, Argentina; MPM, Provincial Regional Museum “Father Jesus Molina,” Río Gallegos, Argentina; MACN, Argentine Museum of Natural Sciences Bernardino Rivadavia, Buenos Aires, Argentina; MMCH, Ernesto Bachmann Paleontological Museum, El Chocón, Argentina; MNBH, Boubou Hama National Museum, Niamey, Niger; MNHN, The French National Museum of Natural History, Paris, France; MPC, Institute of Paleontology and Geology of the Mongolian Academy of Sciences, Ulaanbaatar, Mongolia; MSNM, Milan Natural History Museum, Milan, Italy; NHMUK, Natural History Museum London, London, UK; NMMNH, New Mexico Museum of Natural History, Albuquerque, USA; NCSM, North Carolina State Museum, Raleigh, USA; SGM, Ministère de l'Énergie et des Mines, Rabat, Morocco; TCMI, The Children's Museum of Indianapolis, Indianapolis, USA; UC OBA, University of Chicago, Organismal Biology and Anatomy, Chicago, USA; UCMP, University of California Museum of Paleontology, Berkeley, USA; UMNH, Utah Museum of Natural History, Salt Lake City, USA; USNM, United States National Museum of Natural History, Washington D.C., USA; YPM, Yale Peabody Museum of Natural History, New Haven, USA; ZPAL, Institute of Paleobiology of the Polish Academy of Sciences, Warsaw, Poland.

### 3.3 2D finite element modeling

Most of the models for the analyses were based on 2D technical illustrations of theropod crania in lateral view (Table S1). All drawings were opened with Adobe Illustrator and scaled to the length of the original cranial specimen (in mm) based on the scaled measurements from FIJI as seen in Figure 2, from the posterior end of the quadrate to the anterior end of the premaxilla in lateral view. In addition to scaling the illustrations, we oriented the illustrations (e.g., the crania) horizontally to align the quadrate and ventral extremity of the maxilla. After scaling the illustrations, we traced over the crania of the theropods in lateral view with Illustrator's “Pen Tool.” The digitized images included the outlines of the theropods' crania and the fenestrae (Figure 2a). The digitized images were exported as .dxf files, prior to being imported into the FEA software, COMSOL Multiphysics. While the imported .dxf files reflect the real‐life skull lengths of the theropod crania, the digitized crania were scaled to 1 m, similar to the cranial length of the adult *Tyrannosaurus rex* specimen USNM 555000. Our reasoning for scaling the cranial length was to compare the FEA magnitude and distribution results of tyrannosauroids and non‐tyrannosauroid theropods with different cranial morphology at similar lengths, equivalent forces, and comparable extruded bone thicknesses. We also rendered crestless models of crested theropods (e.g., *Guanlong*, *Monolophosaurus*, *Dilophosaurus*), via the subtraction tool of Adobe Illustrator, by subtracting the digitized points representing the crests. We tested both the crestless and crested theropod models to determine if there was a difference in the level of cranial stress present (e.g., if crested theropod models showed low cranial stress and crestless theropod models showed high cranial stress). This allowed us to test Hypothesis 3, that the crests strengthened the theropods' rostra when biting.

**FIGURE 2:**
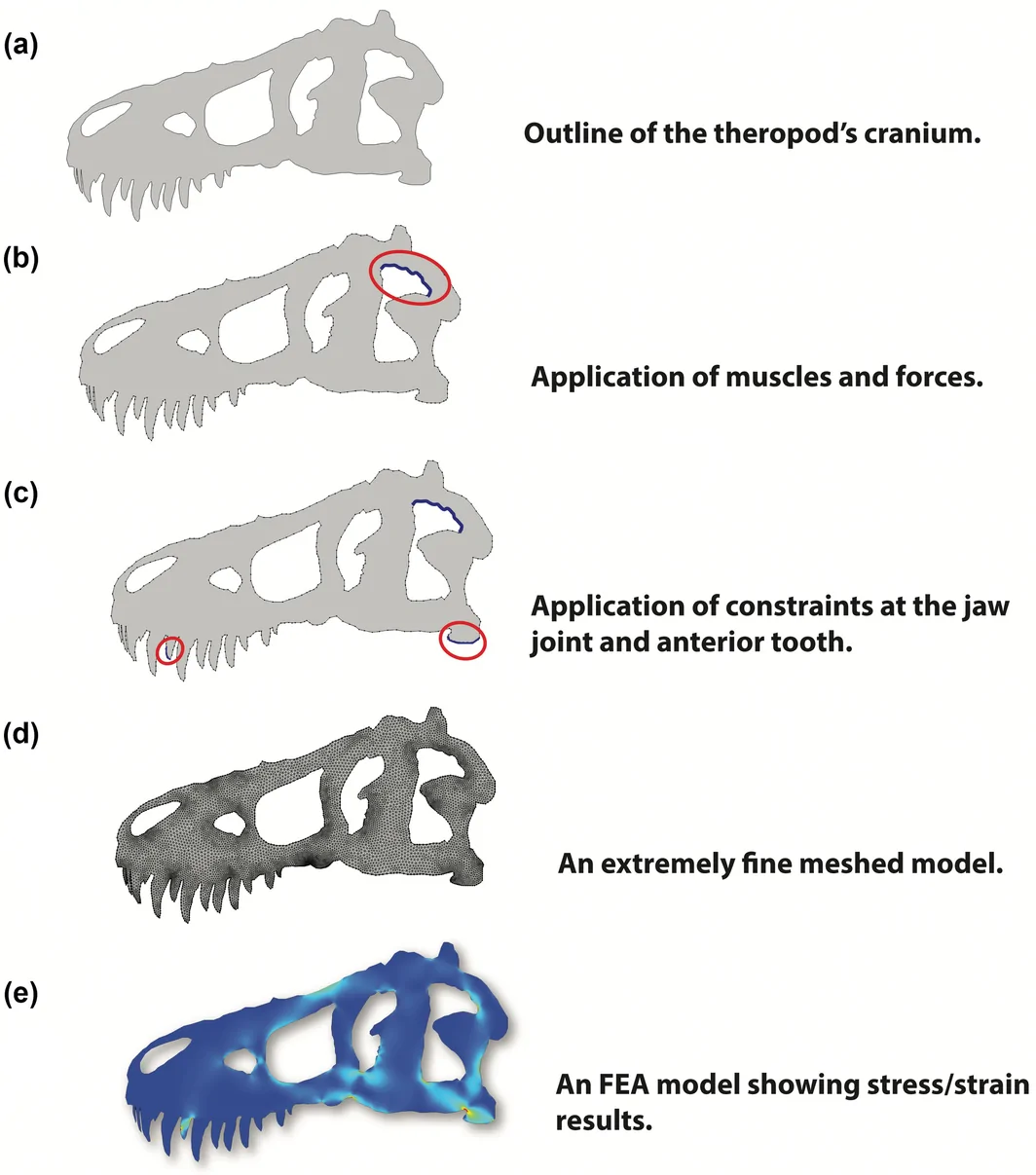
Reconstructing the 2D theropod crania for FEA. (a) Outline of the cranium of the *Tyrannosaurus rex* specimen with the fenestra subtracted (AMNH FARB 5027 and exported as .dxf files into the simulator software, COMSOL Multiphysics. (b) The cranium is assigned bone material properties (e.g., compact bone) and loaded with muscle forces at areas of muscle attachment sites (e.g., the *m. adductor mandibulae externus superficialis* originating at the squamosals). (c) The cranium is constrained at the second maxillary tooth and the jaw joint. (d) The 2D model is converted into a high‐resolution mesh of nodes and elements. (e) The distribution of stress in the 2D FEA model, with the evaluation of von Mises indicating the level of yield or breaking in the cranial models; warm colors indicate high stress and cool colors indicate low stress. The visualized stress was set to 6 MPa, to compare the stress magnitudes of the theropod models.

### 3.4 Parameters and procedures for 2D FEA

The digitized 2D models were imported into COMSOL Multiphysics, where the 2D FEA studies were conducted (Figure 2). We used the 1‐m scaled 2D models to test Hypothesis 1 and Hypothesis 2; that the scaled‐up theropod crania (e.g., *Qianzhousaurus*, *Guanlong*, and *Deinonychus*) display high levels of stress and strain in comparison to large deep‐snouted theropods (e.g., *Tyrannosaurus*, *Daspletosaurus*, *Carnotaurus*, and *Giganotosaurus*). In the Solid Mechanics environment of COMSOL Multiphysics, the cranial profiles were given the same thickness of 0.08 m. Separately, the skull outline and the fenestrae were imported, with the fenestrae subtracted through COMSOL's “Difference” function (Figure 2a). The 2D models were supplied with properties of dense, compact bone (elastic modulus average *E* = 17 GPa, Poisson's ratio 0.3).

To calculate standardized forces for the 2D models (all scaled to 1 m), we applied ratios of muscle force magnitudes for *Tyrannosaurus rex* USNM 555000 (Johnson‐Ransom et al., 2024; based on Gignac & Erickson, 2017 for the former specimen BHI 3033) relative to that of *Musculus pterygoideus ventralis* (the most forceful single muscle: Gignac & Erickson, 2017). The force magnitude of *M. pterygoideus ventralis* was set at 1000 N. Other force magnitudes were scaled accordingly, with 1000 N multiplied by a given muscle's estimated force (Gignac & Erickson, 2017) divided by that of *M. pterygoideus ventralis* (Gignac & Erickson, 2017). We chose the arbitrary 1000 N value for *M. pterygoideus ventralis* to emphasize that the 2D analyses tested relative structural performance, rather than precise comparison of stresses with those of *T. rex* USNM 555000 (Table 1). We simulated the 2D models to perform a static bite for the current analysis.

**TABLE 1:** Jaw Muscle Forces of *Tyrannosaurus* (USNM 555000).

USNM 555000 muscle forces (N)	M.ames	M.amp	M.ptd	M.ptv	Temporal complex	Total force
1.3 m contractile muscle forces (N)	7291	5623	2011	24,281	20,164	59,370

We applied forces as “Boundary Loads” to parts of the skull based on muscle attachment sites (Figure 2b) (Gignac & Erickson, 2017; Holliday, 2009; Lautenschlager, 2012; Rowe & Snively, 2021; Witmer, 1995). The muscle force components were trigonometrically calculated by multiplying the magnitude of muscle force by the sine or cosine of the muscle resultant's angle relative to the horizontal. Because these were 2D models, we had to approximate where the jaw muscles originated, in contrast to the exact attachments of a 3D model. Anterior and posterior constraints placed on the crania simulated biting (Figure 2c). Because the 2D cranial models were scaled at similar lengths and had different proportions (deep‐snouted vs. long‐snouted), the anterior constraints were placed at analogous points (albeit at different tooth positions; e.g., pre‐ and maxillary teeth). The posterior constraints were applied onto the jaw joints for the 2D models. After applying the constraints, we converted the 2D model into a finite element mesh with COMSOL Multiphysics' adaptive setting (smaller, denser elements for smaller details) and “Extremely Fine” resolution with a minimum element length of 0.0205 mm (Figure 2d). Most meshes consisted of 13,000–14,000 triangular internal elements and 1100–1200 boundary elements (Figure 2d). We used the von Mises stress criterion (proximity to failure stress at low strains) to assess the overall magnitude and distribution of stresses displayed in the theropod crania with colors indicating stresses that were least and most significant (Figures 2, 3, 4, 5, 6, 7, 8, 9, 10, 11, 12, 13, 14).

In addition to von Mises stress, we gathered the principal stresses for the 2D models to identify areas of the crania subject to specific forms of stress (see Figures 15 and 16). First principal stress is particularly informative about tensile values, which refer to the response of an object to forces that could tear it apart by pulling or stretching (Figure 15). Third principal stress informatively reflects compression, which refers to the response to forces that cause an object to deform by pushing or crushing (Figure 16). Previous FEA studies have emphasized von Mises stress, whereas first and third principal stresses are less commonly reported in biomechanical studies. We chose to calculate these metrics (first and third principal stresses) because they provide directional information about stress distributions that von Mises stress—a scalar quantity—cannot capture. First principal stress identifies maximum tensile loading, whereas third principal stress identifies maximum compressive loading. These distinctions are particularly important for interpreting the behavior of crania resisting multi‐directional forces during feeding. For regions serving as muscle attachment sites and bones that house teeth, understanding specific tensile versus compressive stress patterns is essential for contrasting structural performance differences.

### 3.5 Representing, visualizing, and comparing stress results

von Mises stress results were used to evaluate the performance of the theropod crania and overall stress magnitudes, with a color scale representing the level of von Mises stress present (e.g., bright red indicating high stress, dark blue indicating low stress; Table S3). Principal stress (Table S4) was also used to evaluate magnitudes of major types of stress present in the 2D models, with first principal stress primarily informative about the level of tension or pulling in the 2D crania (dark blue = low tensile stress, bright red = high tensile stress) and third principal stress reflecting the level of compression or compaction that occurs in the 2D crania (dark red = low compressive stress, light blue = high compressive stress). von Mises stresses were log‐transformed. When qualitatively categorizing the von Mises stress averages, 0.0–2.0 MPa was labeled low stress, 2.1–3.0 MPa being moderate stress, and 3.1+ MPa being high stress (stress values were rounded to the nearest 0.1 MPa).

### 3.6 2D or not 2D: Testing FE stress results from 2D projections against full 3D analyses

This study relies on the interpretation of FE stress results from 2D projections of the lateral sides of theropod crania. To test how realistic and informative the 2D results are for biological interpretations, we compared 2D FE stress distributions against the results of 3D analyses for the tyrannosaurines *Raptorex*, a subadult eutyrannosaurian (BMRP 2002.4.1), and adult *Tyrannosaurus* (Table S2 and Figure 4) (Johnson‐Ransom, 2021; Johnson‐Ransom et al., 2024). The models of the tyrannosauroids parallel an ontogenetic comparison of *Tyrannosaurus* and serve as a more thorough comparative analysis building on results of Johnson‐Ransom (2021). Johnson‐Ransom et al. (2024) details methods of 3D finite element model construction and linear static analyses of high‐resolution meshes in Strand7.

These 3D analyses of tyrannosaur crania incorporated anisotropic material properties of *Alligator* (Porro et al., 2011). Absolute estimates of muscle force (Rowe & Snively, 2021) were scaled from subtemporal fenestra areas of each specimen relative to that of a reference specimen with already‐calculated forces (i.e., former specimen BHI 3033: Gignac & Erickson, 2017). The relative contribution of individual muscles was assumed to be the same as that in the reconstruction of the same specimen (Gignac & Erickson, 2017). The signs of force components reversed as needed from the absolute values in the mandible analyses of Rowe and Snively (2021) of the same specimens. Constraints at the quadrates and anterior maxillary teeth matched constraint positions in the current paper. This comparison is potentially a more stringent test than the 2D versus 3D mandible comparisons of Snively et al. (2010) and Rowe and Snively (2021) because crania are more complex than mandibles.

To quantitatively compare results between the 2D and 3D analyses, we sampled stress values at areas of the cranium of the tyrannosauroid specimens that are analogous to those of the 2D models (e.g., dorsal portion of the nasals in the 3D model of *Tyrannosaurus* and the superoanterior portion of the nasals in the 2D model of *Tyrannosaurus*; Figure 3). We plotted the sampled 2D and 3D von Mises stress values together on respective plots for each animal and compared the values of roots (whole number + decimal) at respective sampled locations (Figure 4). For a statistical comparison, we performed a paired sample *t* test between the sampled 2D and 3D stress values to evaluate significance of differences between the 2D and 3D models.

**FIGURE 3:**
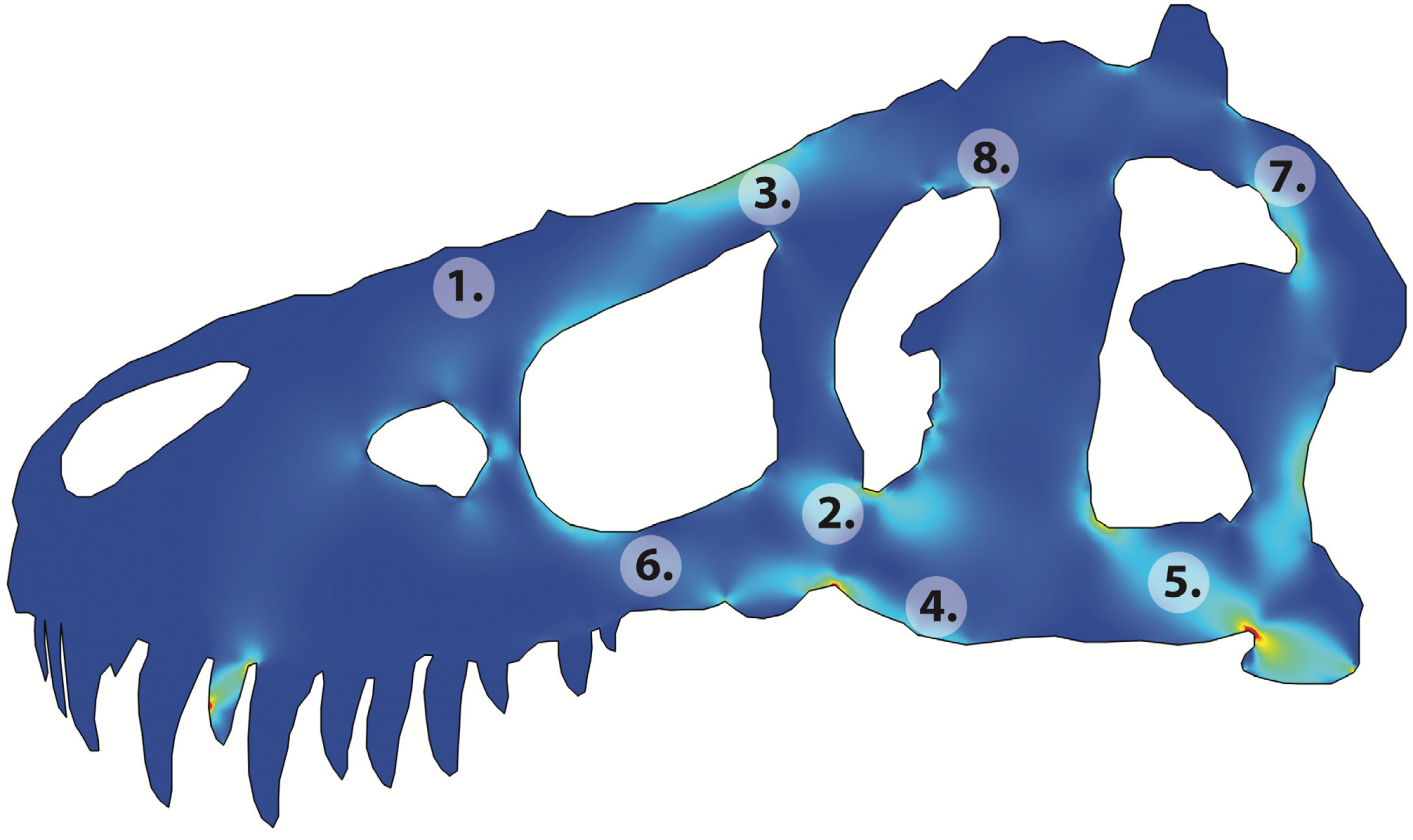
von Mises stress sampling in the 2D crania of theropods (e.g., *Tyrannosaurus* specimen AMNH FARB 5027). The homologous sampled cranial locations include the nasals (1), lacrimal (2), nasal‐lacrimal suture (3), inferior region of the jugal (4), posterior region of the jugal (5), maxilla‐jugal suture (6), squamosal (7), and postorbital‐lacrimal suture (8).

**FIGURE 4:**
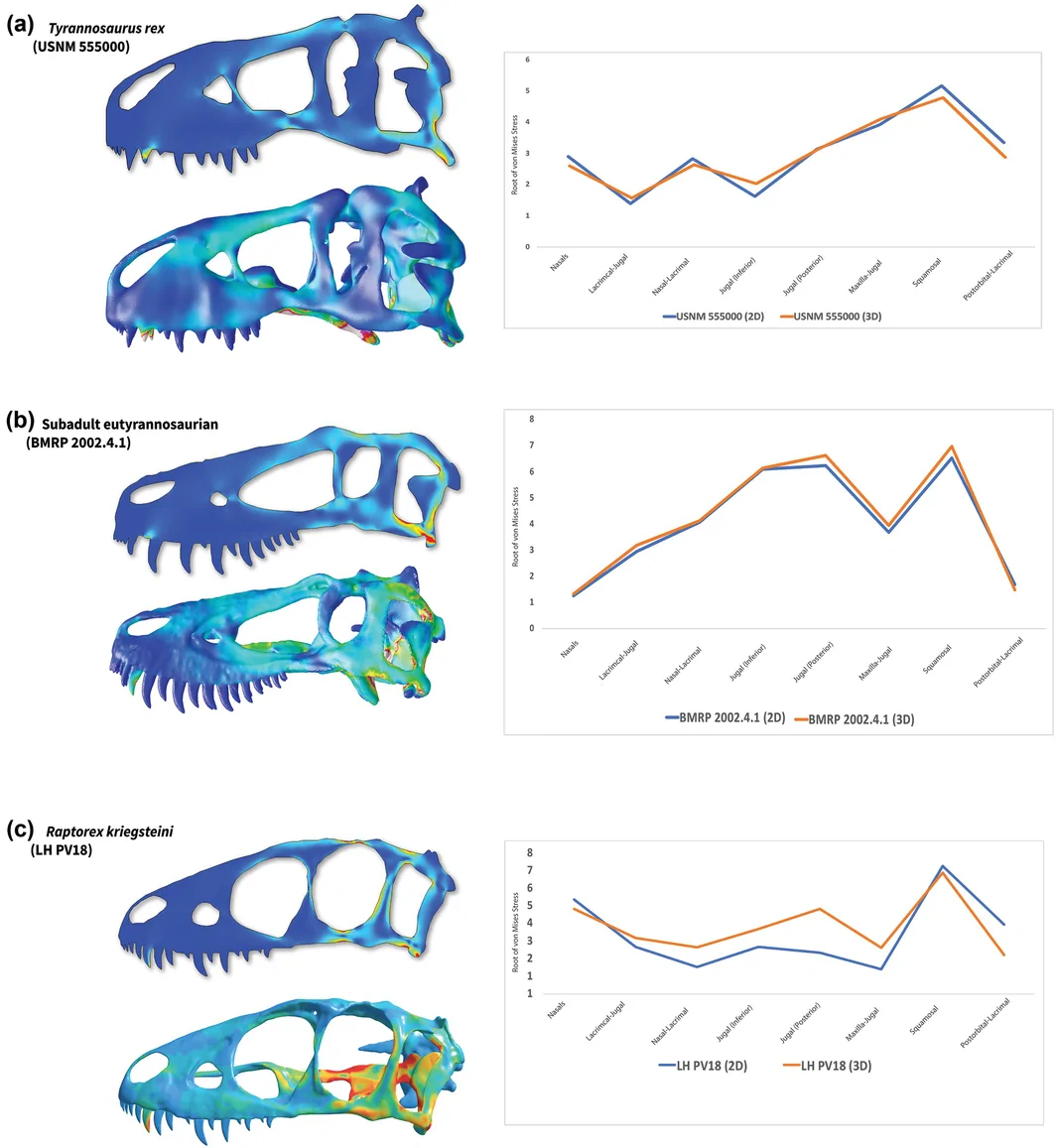
von Mises comparisons of the 2D (top) and 3D (bottom) for adult *Tyrannosaurus*, subadult eutyrannosaurian (BMRP 2002.4.1), and *Raptorex* models. The sampled roots of 2D and 3D von Mises stress in the adult and juvenile *Tyrannosaurus* specimens were nearly identical, with more variance but a similar pattern in *Raptorex*. The stress values on the y‐axis are unitless, given the different orders of magnitude of the 2D and 3D results. The *p*‐values for evaluating significant stress differences in the tyrannosauroid specimens were 0.5348 (USNM 555000), 0.346 (BMRP 2002.4.1), and 0.07526 (LH PV18).

### 3.7 Phylogenetic hypothesis

We generated a tree topology by manually constructing a cladogram reflecting the results of previous cladistic studies of theropod relationships in RStudio version 2024.12.0 + 467 (Figures 17 and 18) (Brusatte et al., 2014; Brusatte & Carr, 2016; Carrano et al., 2012; Choiniere et al., 2014; Ezcurra & Brusatte, 2011; Godefroit et al., 2013; Hartman et al., 2019; Loewen et al., 2013; Lü et al., 2014; Naish & Cau, 2022; Pol & Rauhut, 2012; Porfiri et al., 2014; Rolando et al., 2022; Smith et al., 2007; Sues et al., 2011; Tortosa et al., 2014; Turner et al., 2012). To generate divergence time estimates, we used the fossilized birth‐death (FBD) tip‐dating model (Heath et al., 2014) implemented in MrBayes (Huelsenbeck & Ronquist, 2001; Ronquist & Huelsenbeck, 2003). First, we collected first and last occurrence dates for each taxon from the Paleobiology Database. When the date of occurrence was imprecise (e.g., “Early Cretaceous”), we sought and used dates for the geological formations of occurrence for relevant sister groups, with the earliest sister taxon anchoring the beginning age of a taxon's duration. Recent FBD tip‐dating analyses have demonstrated that MrBayes can ignore topological constraints at the root (Herrera‐Flores et al., 2021). Thus, we added two additional taxa as outgroups to retain the topology of our focal clade: *Tawa* and *Eoraptor*. In order to maintain correct time scaling of the dated phylogeny in millions of years from the present, it is essential to have at least one extant taxon included in the topology, so we added *Passer* as the sister to Deinonychosauria. We then generated a MrBayes control file using the “createMrBayesTipDatingNexus” function in the paleotree v3.3.25 R package (Figure 17) (Bapst, 2012). We used default recommended priors (Matzke & Wright, 2016) and ran the analysis for 400,000 generations. For each subsequent analysis, we used the maximum clade credibility (MCC) tree and a random sample of 10 dated trees. *Passer*, *Tawa*, and *Eoraptor* were pruned from the tree before proceeding.

### 3.8 Phylogenetic comparative analyses

We quantified phylogenetic signal (Pagel's lambda) in mean von Mises stress using the “phylosig” function in the phytools v1.0‐1 R package (Pagel, 1999; Revell, 2012). Across the 10 randomly sampled trees, the mean phylogenetic signal was *λ* = 0.41 (standard deviation = 0.07), indicating a moderate influence of phylogeny on stress. As such, we used phylogenetically informed analyses to test subsequent hypotheses. To test whether stress is correlated with head size, we carried out phylogenetic generalized least squares regressions (Freckleton et al., 2002; Ksepka et al., 2020). We fit regressions for each of the 10 random trees using the estimated lambda value for that tree, with log‐transformed head length as the predictor variable and log‐transformed average von Mises stress as the response variable.

### 3.9 Ancestral state reconstructions

The purpose of using an ancestral state reconstruction was to test for evolutionary patterns within the internal branches of the clade Tyrannosauroidea and other theropod groups (Figures 18 and [Link], [Link]). We incorporated non‐tyrannosauroid theropods for a broader phylogenetic comparison with tyrannosauroids. Ancestral state reconstructions allow us to evaluate and infer the level of stress magnitudes present in the internal branches of the theropod clades (e.g., Tyrannosauroidea, Allosauroidea, Megalosauroidea).

We used stress magnitude as a complex character with a continuous state value. Here, stress is calculated based on integrated phenotypes (Pigliucci, 2003) of skull shape, material property values, and muscle configuration, coupled with instantaneous behavioral inputs of consistent tooth position and joint constraints, and loading through static jaw‐muscle contraction and reaction forces (see above). By standardizing skull size dimensions, material properties modeled, number of boundary nodes, and locations of forces applied, we ensure that the output stresses from the skull models of our sampled taxa represent a comparable complex character state. We, thus, treat stress magnitude as analogous to other previously studied complex characters that represent behavioral repertoires (Price & Lanyon, 2002), growth trajectories (Erickson et al., 2004; Sander et al., 2004), and allometry (Ksepka et al., 2020)—each of which is similarly shaped by numerous genetic and environmental inputs such that standardization is necessary to derive meaningful comparisons. We interpret this approach to provide valuable insights into evolutionary trends based on mechanical performance metrics, particularly for evolutionary questions focused on the fossil record. For example, later diverging theropod clades may show lower stress magnitudes than early diverging theropod clades (e.g., Coelophysoidea). Ancestral character state reconstruction was conducted using the “contMap” function in phytools, which calculated the maximum likelihood estimate for the character state at each node (Revell, 2012). The sampled von Mises values of the theropod crania were averaged and used as a summational representation of cranial stress of the theropod clade (e.g., Tyrannosauroidea, Megalosauroidea) for broader phylogenetic comparisons. The color scale of the key and branch lengths are analogous to the color scale gradient of von Mises stress.

We also explored ancestral character states for stress magnitude at specific cranial regions (e.g., nasals, jugal, and nasal‐lacrimal suture) using squared change parsimony in the phylogenetics software Mesquite (version 3.81). The selected cranial regions (points) have biomechanical significance, either acting as resistance to bending or attachments for adductor jaw muscles (Figure 3 and Table 2) (Cost et al., 2020; Hurum & Sabath, 2003; Johnson‐Ransom et al., 2024; Rayfield, 2004; Snively et al., 2006). This model‐free approach allowed us to characterize the biomechanical performance of each clade using phylogenetically informed ancestral values derived from minimizing sums of squared changes along the branches of the phylogenetic tree (Maddision, 1991). The unweighted squared change parsimony analysis does not require divergence time estimates (Maddision, 1991) and therefore allows for comparison of stresses among theropod clades independent of the uncertainty in divergence times for some clades in the dataset.

**TABLE 2:** Sampled Cranial Regions for Ancestral Character State Estimation.

Cranial region	Biomechanics significance	Source
Nasals	Resistance to shear, bending, and torsion	Snively et al. (2006)
Lacrimal	Redirection of stress during feeding	Hurum and Sabath (2003) and Rayfield (2004)
Nasal‐lacrimal suture	Resistance to tension and bending	Snively et al. (2006) and Rayfield (2004)
Inferior region of jugal	Resistance to tension, and position of 2D attachment of the pterygoid muscles	Rayfield (2004)
Posterior region of jugal	Resistance to tension, and position of 2D attachment of the pterygoid muscles	Johnson‐Ransom et al. (2024)
Maxilla‐jugal suture	Akinetic articulation of the cranium	Cost et al. (2020)
Squamosal	Attachment for the m. adductor externus superficialis	Johnson‐Ransom et al. (2024) and Cost et al. (2020)
Postorbital‐lacrimal suture	Akinetic articulation of cranium and resistance to shear	Cost et al. (2020) and Rayfield (2004)

## 4 RESULTS

The following results detail von Mises, first, and third principal stresses. von Mises stress is a function of principal stresses and describes overall stress levels, and we interpret relative von Mises values as low or high overall stress. First and third principal stresses reflect primarily tensile and almost exclusively compressive stress, respectively. For intuitive communication, we therefore report first and third principal stresses as tensile and compressive. Figures for these quantities depict any compressive components of first principal stress and tensile components of third principal stress.

### 4.1 2D and 3D comparisons

The 2D and 3D models of the tyrannosaurids converge well in patterns of relative magnitude of von Mises stress, although they are not identical (Figure 4). The 2D *Raptorex* model has high stress concentration occurring in the temporal region and bones encompassing the orbital fenestra, while the 3D *Raptorex* model has high stress magnitudes concentrating in the squamosals and quadrates (Figure 4c). The 2D subadult eutyrannosaurian (BMRP 2002.4.1) model shows overall low cranial stress with a small portion of high stress occurring at the jaw joint; the 3D subadult eutyrannosaurian (BMRP 2002.4.1) model has high stress occurring at regions of the cranium similar to the 3D *Raptorex* model (e.g., squamosals and quadrate; Figure 4b). Both the 2D and 3D models of the adult *Tyrannosaurus* show moderately low cranial stress, with high stress occurring in the jaw joint for both models (Figure 4a). The 3D cranial bones of the adult *Tyrannosaurus* (USNM 555000) and subadult eutyrannosaurian (BMRP 2002.4.1) show similar values that were analogous to similarly sampled portions in the 2D cranial models of the adult *Tyrannosaurus* and subadult eutyrannosaurian. The 2D and 3D models of *Raptorex* differed in relative values but were consistently off by <1 integer value of roots. While the 2D and 3D models differed in stress magnitudes, the models show consistent stress values being only separated by units (Pascals and MegaPascals). The analogously sampled cranial locations with consistent relative stress values validate the use of 2D FEA.

### 4.2 Predicted patterns of cranial stress through proxies for tyrannosaur ontogeny

*Raptorex* is a juvenile tyrannosaurine and reasonably serves as a model for other early‐stage juvenile tyrannosaurids (e.g., *Tarbosaurus*, *Tyrannosaurus*) based on its immature cranial anatomy and histologically determined young age (Fowler, Woodward, et al., 2011; Hurum & Currie, 2000; Hurum & Sabath, 2003; Johnson‐Ransom et al., 2024; Maleev, 1955, 1974; Rowe & Rayfield, 2024; Rowe & Snively, 2021; Saveliev & Alifanov, 2007; Tsuihiji et al., 2011). Similar to *Raptorex*, the subadult eutyrannosaurian (BMRP 2002.4.1) identified as either *Nanotyrannus lethaeus* (Zanno & Napoli, 2025) or *Tyrannosaurus rex* (e.g., Carr, 2020; Woodward et al., 2020) serves as a model for late‐stage juvenile tyrannosaurines, given its immature status compared to *Nanotyrannus lancensis* (NCSM 40000) and BMRP 2002.4.1 having a relatively gracile skull and hindlimb morphology, traits likely present in late‐stage juvenile tyrannosaurines (e.g., *Tyrannosaurus* and *Tarbosaurus*; Johnson‐Ransom et al., 2024; Rowe & Snively, 2021; Zanno & Napoli, 2025). The juvenile tyrannosaurine *Raptorex* and the subadult eutyrannosaurian (BMRP 2002.4.1) show high stress concentrations occurring at the more distal region of the cranium and jugal, as well as the nasal‐lacrimal connection. Consistent with the 3D analyses, as tyrannosaurine body size increased, the adult crania became more deeply robust. Lower stress magnitude occurs in the rostral region of the crania, and a small portion of high stress occurs at the ventral ridge of the squamosal bar and jaw joint (Johnson‐Ransom, 2021; Johnson‐Ransom et al., 2024) (Figure 4).

### 4.3 Theropod clade von Mises and principal stress comparisons

#### 4.3.1 Non‐averostran theropods

*Coelophysis* shows moderately low stress at the dorsal surface of the nasals (1.3 MPa), squamosals (1.1 MPa), and jugal (1.9 MPa), forming a pattern similar to those of basal tyrannosauroids (e.g., *Guanlong*) (Figures 5f and 8b). Similar to *Guanlong* and *Monolophosaurus*, *Dilophosaurus* shows low von Mises stress at the crests, with moderately low stress occurring at the rostral‐most jugal (1.0 MPa) and nasals (0.2 MPa; UCMP 77270) (Figures 6f and 8a). *Coelophysis* and *Dilophosaurus* have an overall average von Mises stress of 1.4 and 0.9 MPa, respectively, indicative of relatively low cranial stress magnitudes (Figures 5f, 6f, 8, S1, and S2).

**FIGURE 5:**
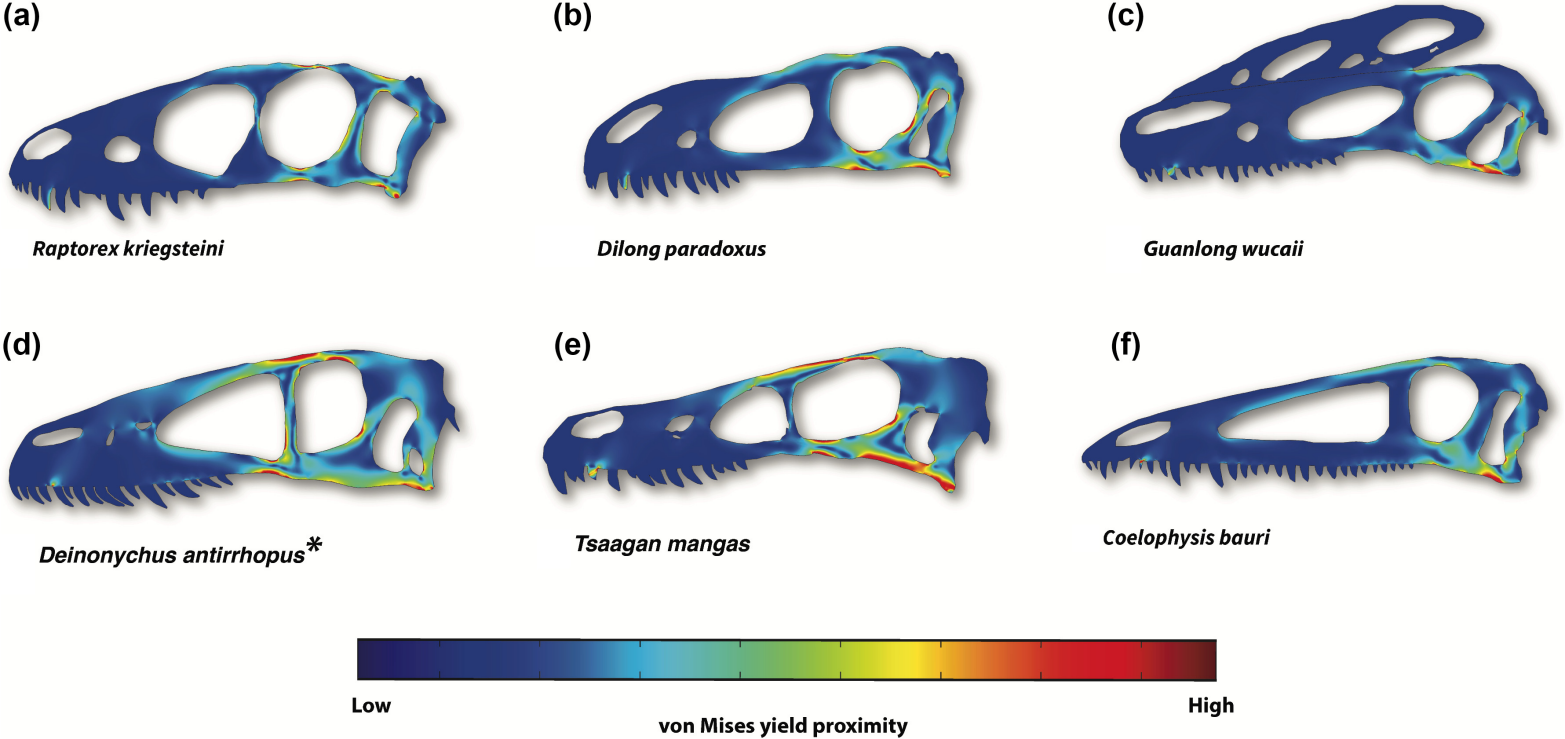
von Mises stress (megapascals) in planar models of small, early tyrannosauroids [*Dilong paradoxus* (b), *Guanlong* wucaii (c)], juvenile tyrannosaurids [*Raptorex kriegsteini* (a)], and other small theropods [e.g., dromaeosaurids and *Coelophysis bauri* (f)] scaled to the length (1 m) and muscle force of adult *Tyrannosaurus rex* (USNM 555000); “*” indicates that the theropod model is a composite. Dromaeosaurid crania [*Deinonychus antirrhopus* (d) and *Tsaagan mangas* (e)] show greater von Mises stress than small early tyrannosauroids and juvenile tyrannosaurids. 6 MPa is the greatest visualized stress for the theropod crania.

**FIGURE 6:**
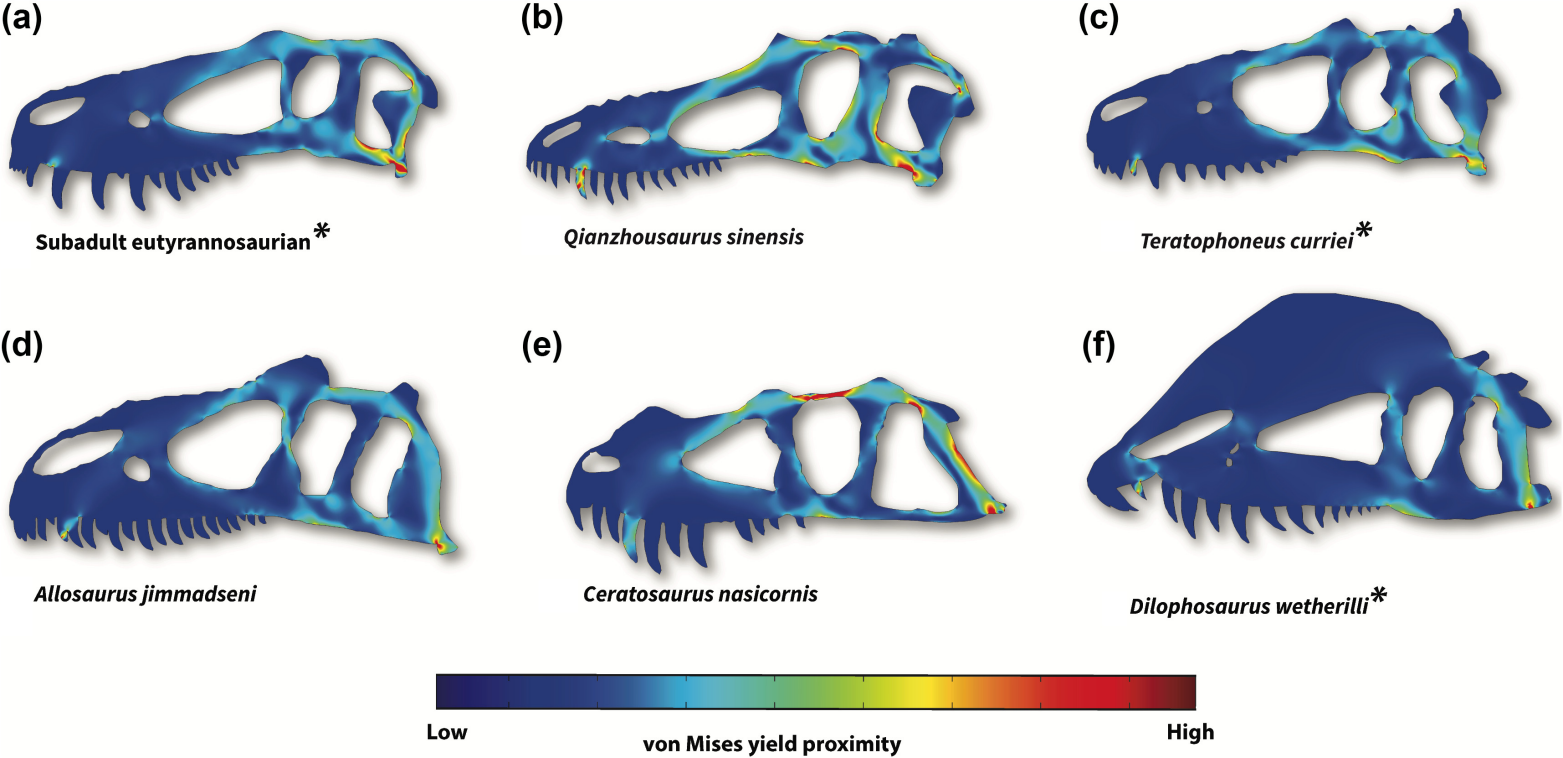
von Mises stress (megapascals) in planar models of medium‐sized tyrannosauroids [subadult eutyrannosaurian (BMRP 2002.4.1) (a), *Qianzhousaurus sinensis* (b), and *Teratophoneus curriei* (c)] and other medium‐sized theropods [*Allosaurus jimmadseni* (d), *Ceratosaurus nasicornis* (e), and *Dilophosaurus wetherilli* (f)] scaled to the length (1 m) and muscle force of adult *Tyrannosaurus rex* (USNM 555000). “*” indicates that the theropod model is a composite. Theropods with relatively gracile crania and shallow snouts (e.g., medium‐sized tyrannosauroids and [*Ceratosaurus* (e)]) show relatively greater von Mises stress than medium‐sized theropods with the latter having short and robust crania (e.g., [*Allosaurus* (d)]). 6 MPa is the greatest visualized stress for the theropod crania.

*Coelophysis* exhibits moderately low tensile stress, with prominent peaks occurring at the maxillary‐jugal connection and jugal (Figure S1b). *Coelophysis* tensile stress is lower than most similarly sized theropods (e.g., *Dilong* and *Tsaagan*). *Coelophysis* shows low compressive stress at the dorsal surface of the nasals, with prominent peak stress occurring at the squamosal and quadrate (Figure S2b). *Dilophosaurus* shows low tensile stress in its cranium, with peak stress occurring at the jugal (UCMP 77270) and low stress occurring at the crests (Figure S1a). *Dilophosaurus* shows low compressive stress, with peak stress occurring at the temporal complex of its cranium (UCMP 77270) (Figure S2a).

#### 4.3.2 Ceratosauria

In the ceratosaurids, *Ceratosaurus* has an average von Mises cranial stress of 1.8 MPa, with high von Mises stress magnitudes occurring at the articulation between the lacrimal and postorbital (5.2 MPa), ventral region of the squamosal (2.1 MPa), quadrate, and quadratojugal (Figures 6e and 9f). Moderately low stress occurs at the lacrimal crests (1.1 MPa), nasals (0.6 MPa), and temporal region of *Ceratosaurus*. *Masiakasaurus* has a relatively greater von Mises stress average of 1.9 MPa, with high von Mises stress magnitudes in the postorbital (2.9 MPa; FMNH PR 2457) and low stress in the lacrimal (1.1 MPa; FMNH PR 2473), analogous to gracile‐snouted dromaeosaurids and basal tyrannosauroids (Figure 9e). Most of the abelisaurids show low cranial stress magnitudes with some abelisaurids having localized areas of high stress occur at the quadratojugal, similar to the deep‐snouted tyrannosaurids (Figure 9a–d). *Abelisaurus* (average von Mises stress of 1.5 MPa) shows moderately high von Mises stress at the nasals (1.1 MPa), temporal region, quadratojugal, and maxilla (Figure 9d). Short‐snouted abelisaurids such as *Majungasaurus* (average von Mises stress of 0.8 MPa; Figure 9b), *Skorpiovenator* (average von Mises stress of 1.0 MPa; Figure 9c), and *Carnotaurus* (average von Mises stress of 0.8 MPa; Figure 9a) have a small portion of high stress at the quadratojugal, while having primarily low stress throughout the crania. Among the ceratosaurian theropods, the abelisaurids generally showed a von Mises cranial stress average of 0.8–1.5 MPa, which was relatively lower than *Ceratosaurus* (1.8 MPa) and *Masiakasaurus* (1.9 MPa). Abelisaurids generally show lower von Mises stress magnitudes than other ceratosaurian clades (Figures 6e, 9, S3, and S4).

*Ceratosaurus* shows moderately low tensile stress at the temporal complex and postorbital, with prominent tensile peaks at the maxilla and jugal (Figure S3f). *Ceratosaurus* has high compressive stress at the nasals, temporal complex, lacrimal, postorbital, and quadratojugal (Figure S4f). *Masiakasaurus* shows moderately low tensile stress, with prominent peaks occurring at the maxilla and jugal (mirrored from FMNH PR 2183; Figure S3e). *Masiakasaurus* shows low compressive stress in its cranium, with prominent stress peaks occurring at the frontals (FMNH PR 2496; Figure S4e). Most of the abelisaurids exhibit low tensile stress analogous to that of deep‐snouted tyrannosaurids, with low tensile stress being present at the nasal‐lacrimal connection, squamosal, and the jugal (Figure S3a–d). The deep‐snouted *Carnotaurus* (Figure S3a) and *Abelisaurus* (Figure S3d) show greater tensile stress peaks at the maxillae than *Majungasaurus* (Figure S3b) and *Skorpiovenator* (Figure S3c). The abelisaurids (Figure S4a–d) show low compressive stress at the nasals and temporal complex, with *Abelisaurus* (Figure S4d) showing greater stress peaks than the short‐faced abelisaurids.

#### 4.3.3 Megalosauroids and Monolophosaurus

Megalosaurids such as *Torvosaurus* (Figure 10d), *Afrovenator* (Figure 10e), and *Dubreiullosaurus* (Figure 10f) display high von Mises stress at the quadrate (*Torvosaurus*' quadrate BYUVP 5110; Figure 10d). Moderately low stress occurs in the ventral portions of the maxillae (1.0 MPa in *Afrovenator*, 1.3 MPa in *Dubreuillosaurus*, and 1.1 MPa in *Torvosaurus*) and quadratojugals in the crania of megalosaurids. Among the megalosaurids, *Torvosaurus* and *Afrovenator* show von Mises cranial stress averages of 1.6 and 1.3 MPa, relatively greater than *Dubreuillosaurus* (1.9 MPa). Spinosaurids were initially expected to show high levels of von Mises stress (average von Mises stress of 3.0 MPa or greater), but the calculated results show the spinosaurids to have relatively low cranial stress (except for *Baryonyx*; Figure 10c). *Spinosaurus* and *Suchomimus* show cranial von Mises stress averages of 1.2 and 1.8 MPa, respectively, in contrast to the *Baryonyx*'s von Mises stress average of 2.4 MPa. The baryonychine spinosaurids show von Mises stress occurring in the nasals, with *Suchomimus* (1.9 MPa; Figure 10b) showing lower nasal stress than *Baryonyx* (2.5 MPa); the latter has a proportionally longer snout than *Suchomimus*. *Suchomimus* shows moderately low von Mises stress occurring at the anterior jugal (1.3 MPa) and quadratojugal. *Baryonyx* shows higher von Mises stress that is present at the ventral portion of the maxilla (2.4 MPa), lacrimal (2.5 MPa), postorbital bar, quadrate, and quadratojugal. The large spinosaurine, *Spinosaurus* (Figures 7c and 10a), shows lower von Mises stress than the baryonychines, moderately low stress occurring at the dorsal portion of the nasals (1.3 MPa; MSNM V4047) and maxilla‐jugal suture (0.6 MPa; based on *Irritator* SMNS 58022), as well as moderately high stress present in the quadrate (based on *Irritator* SMNS 58022). Very low stress is also present in the nasal crest of *Spinosaurus* (FSAC‐KK‐11888). The cranium of *Spinosaurus* differs from the baryonychines', given the former having a taller and more robust cranium. The basal tetanuran *Monolophosaurus* shows moderately low stress in the quadratojugal, ventral portion of the maxilla (1.4 MPa), and dorsal surface of the quadrate (Figure 10g). Moderately low stress is present in the squamosal bar (1.9 MPa) and temporal complex of *Monolophosaurus*. Similar to *Spinosaurus*, low stress is present in the crest of *Monolophosaurus*. Spinosaurids show a general cranial von Mises stress average of 1.2–2.4 MPa, with megalosaurids having a cranial von Mises stress average of 1.3–1.9 MPa. Except for *Baryonyx*, spinosaurids and megalosaurids show a consistent von Mises stress average (Figures 7c, 10, S5, and S6).

**FIGURE 7:**
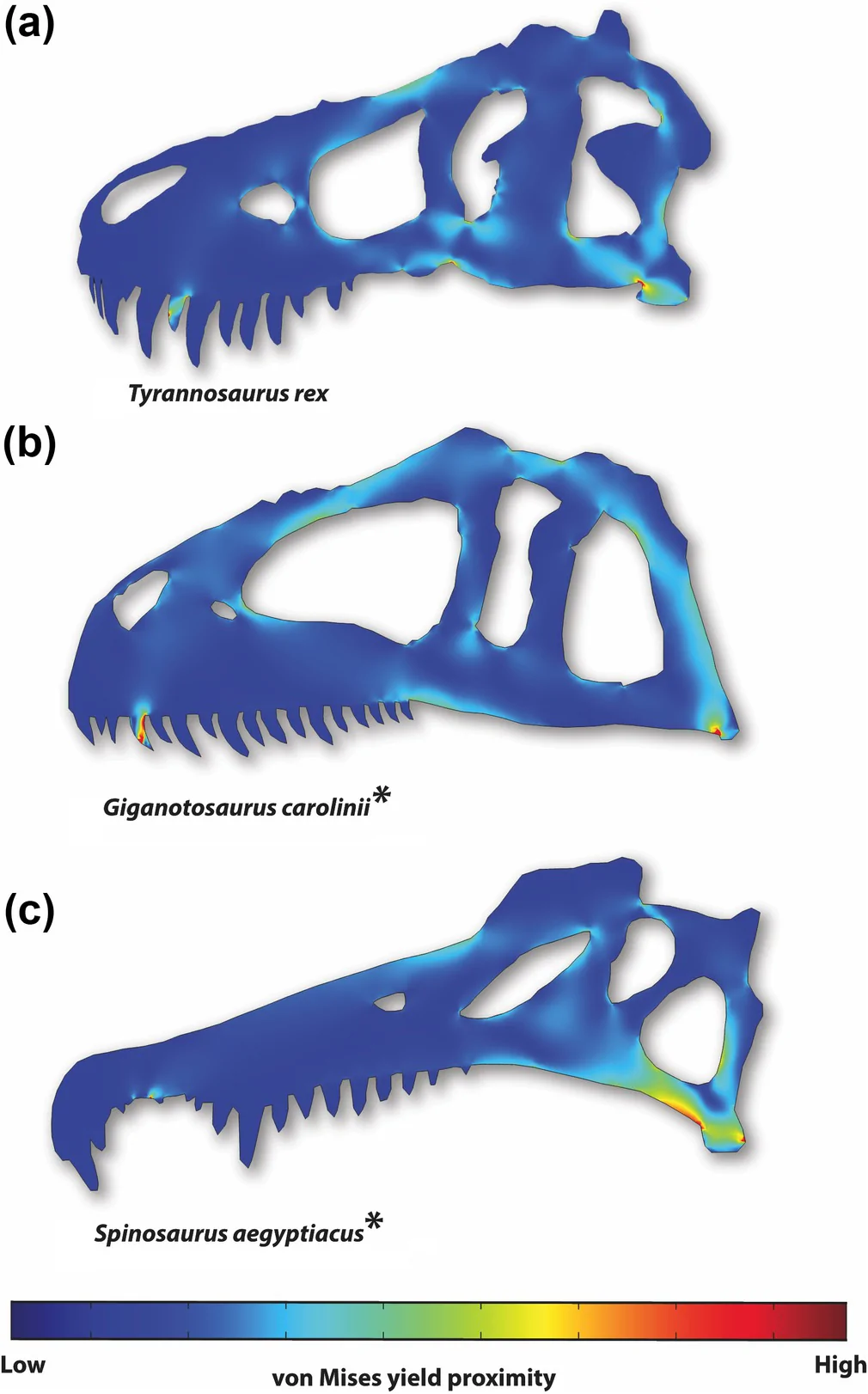
von Mises stress (megapascals) in planar models of large theropods [*Tyrannosaurus* rex (a), *Giganotosaurus carolinii* (b), and *Spinosaurus aegyptiacus* (c)] scaled to the length (1 m) and muscle force of adult *Tyrannosaurus* (USNM 555000); (*) indicates theropod model being a composite. The crania of *Giganotosaurus* (b) and *Spinosaurus* (c) show greater von Mises stress than the adult *Tyrannosaurus* (a). 6 MPa is the greatest visualized stress for the theropod crania.

**FIGURE 8:**
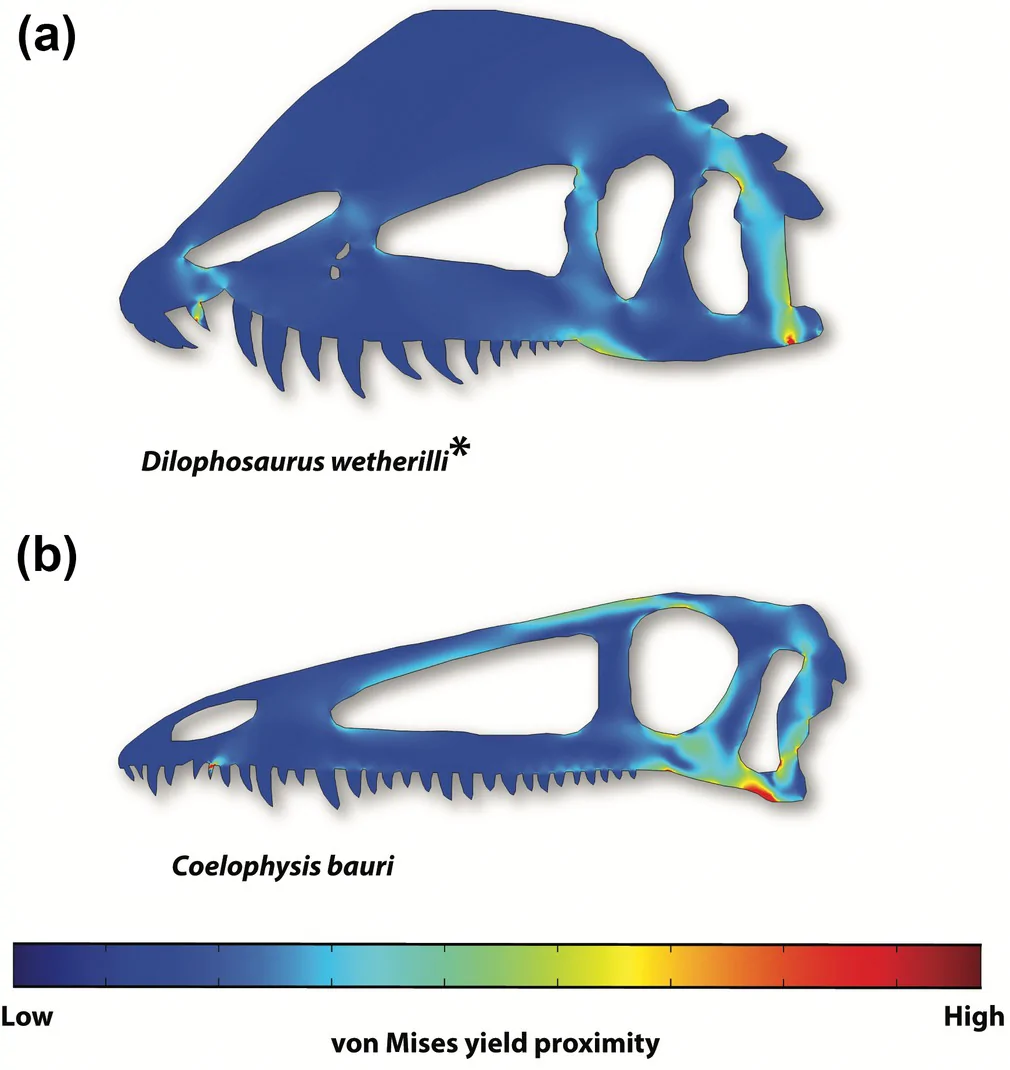
von Mises stress (megapascals) in planar models of the early theropods *Coelophysis bauri* (b) and *Dilophosaurus wetherilli* (a), scaled to the length (1 m) and muscle force of adult *Tyrannosaurus rex* (USNM 555000); (*) indicates theropod model being a composite. Stress is greater (hotter colors) in the small theropod *Coelophysis bauri* (b) than in *Dilophosaurus wetherilli* (a). The cranial stress is analogous to compsognathids and early tyrannosauroids (e.g., temporal complex, postorbital, and quadratojugal). Like the crested proceratosaurids (e.g., *Guanlong*), *Dilophosaurus wetherilli* (a) exhibits little substantial stress in the crest. 6 MPa is the greatest visualized stress for the theropod crania.

**FIGURE 9:**
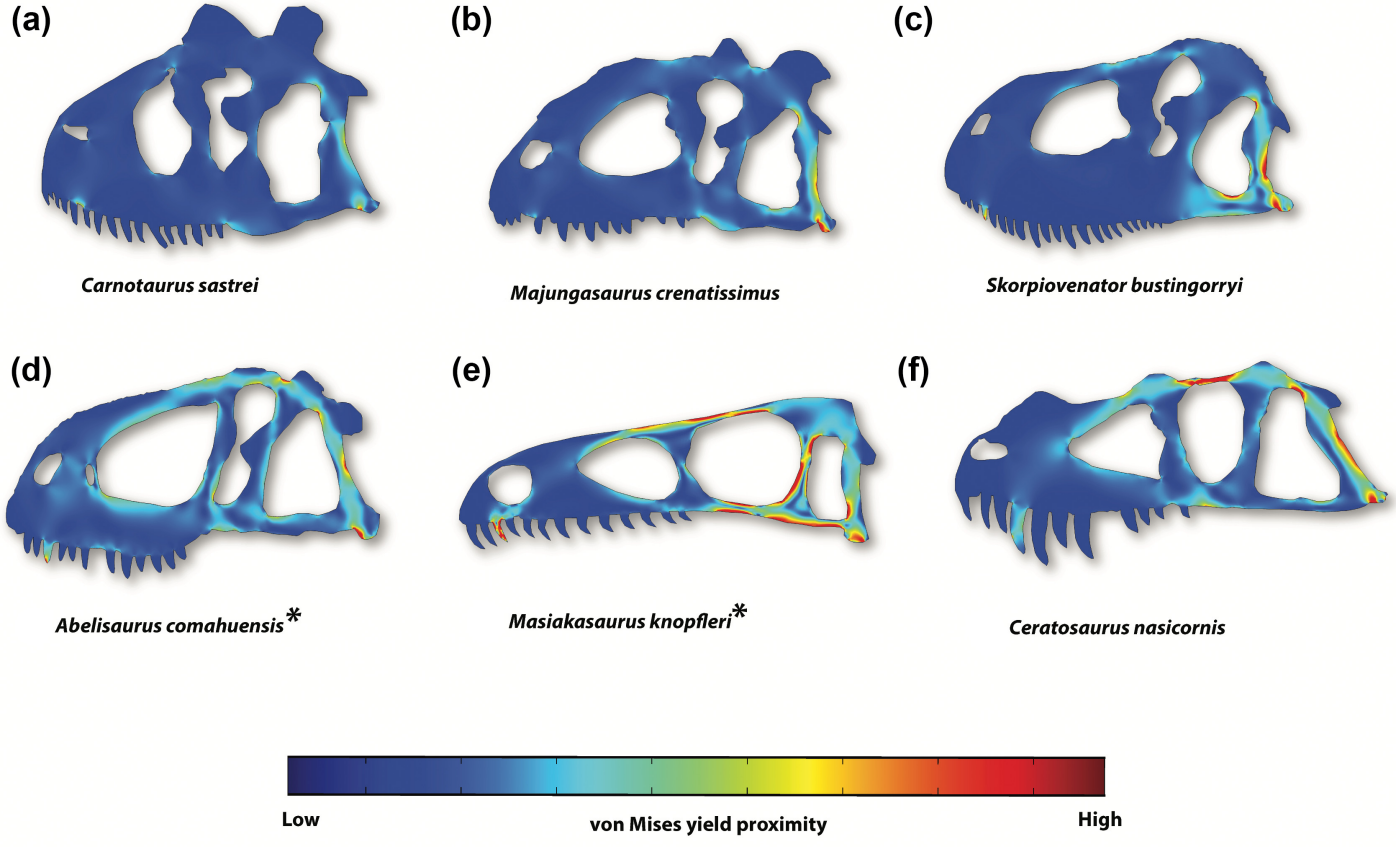
von Mises stress (megapascals) in planar models of ceratosaurian theropods, scaled to the length (1 m) and muscle force of adult *Tyrannosaurus rex* (USNM 555000); (*) indicates that the theropod model is a composite. *Ceratosaurus nasicornis* (f) and *Masiakasaurus knopfleri* (e) show high cranial stress in the temporal complex, postorbital bar, and quadratojugal. When scaled to the same length of an adult *T. rex*, abelisaurids [*Carnotaurus sastrei* (a), *Majungasaurus crenatissimus* (b), *Skorpiovenator bustingorryi* (c), and *Abelisaurus comahuensis* (d)] show primarily low cranial stresses (cool colors). 6 MPa is the greatest visualized stress for the theropod crania.

**FIGURE 10:**
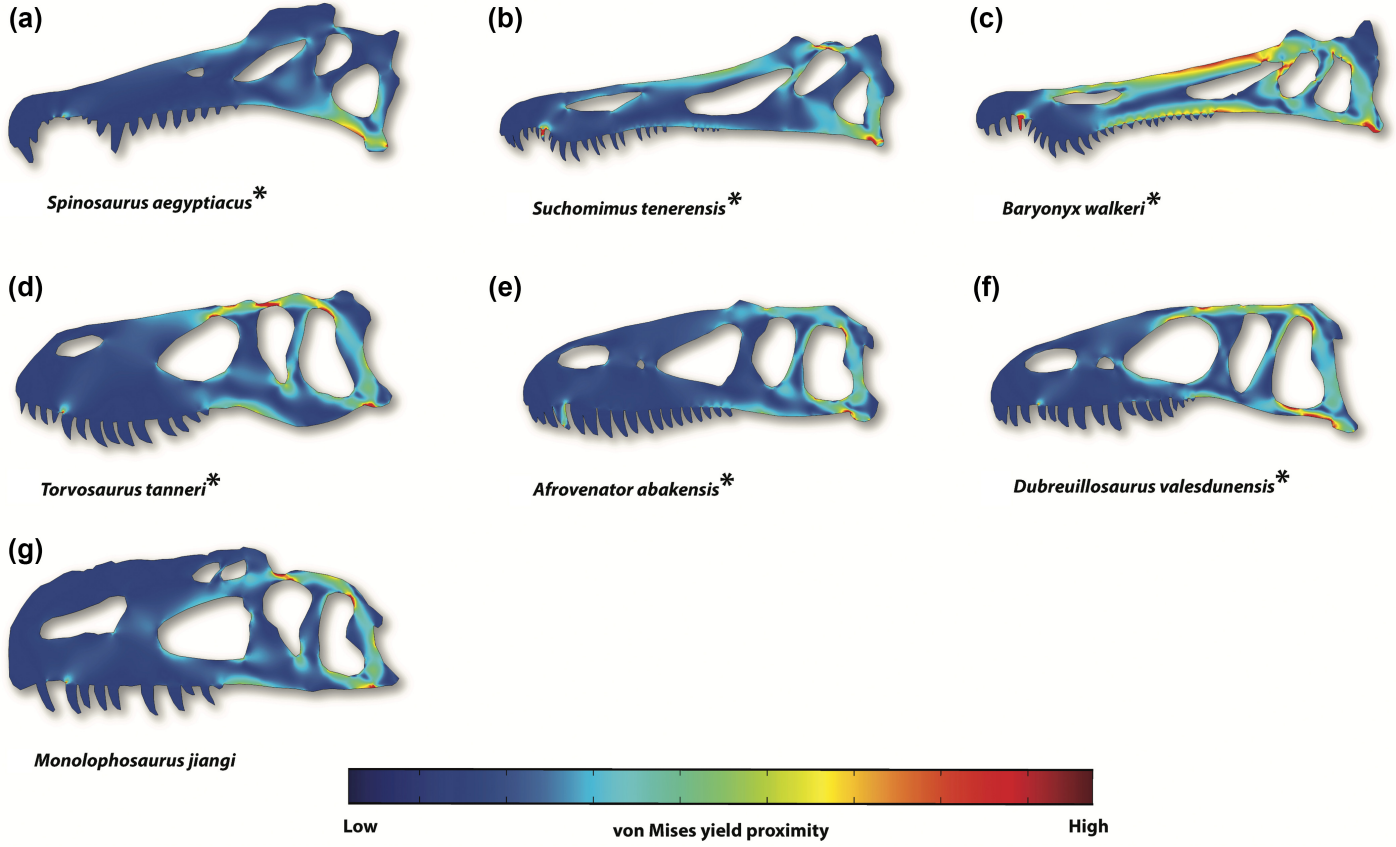
von Mises stress (megapascals) in planar models of megalosauroid theropods and basal tetanuran [*Monolophosaurus jiangi* (g)], scaled to the length (1 m) and muscle force of adult Tyrannosaurus rex (USNM 555000); (*) indicates that the theropod model is a composite. The megalosaurids [e.g., *Torvosaurus tanneri* (d), *Afrovenator abakensis* (e), and *Dubreuillosaurus valesdunensis* (f)] show lower cranial stresses than the slender‐snouted spinosaurids. The level of cranial stress decreases with increasing spinosaurid body size, with *Spinosaurus aegyptiacus* (a) showing the lowest cranial stress, *Suchomimus tenerensis* (b) having the second lowest cranial stress, and *Baryonyx walkeri* (c) showing the highest cranial stress. *Monolophosaurus jiangi* exhibits cranial stress patterns analogous to megalosaurids, with low stress occurring at the crest, similar to *Spinosaurus aegyptiacus*. 6 MPa is the greatest visualized stress for the theropod crania.

**FIGURE 11:**
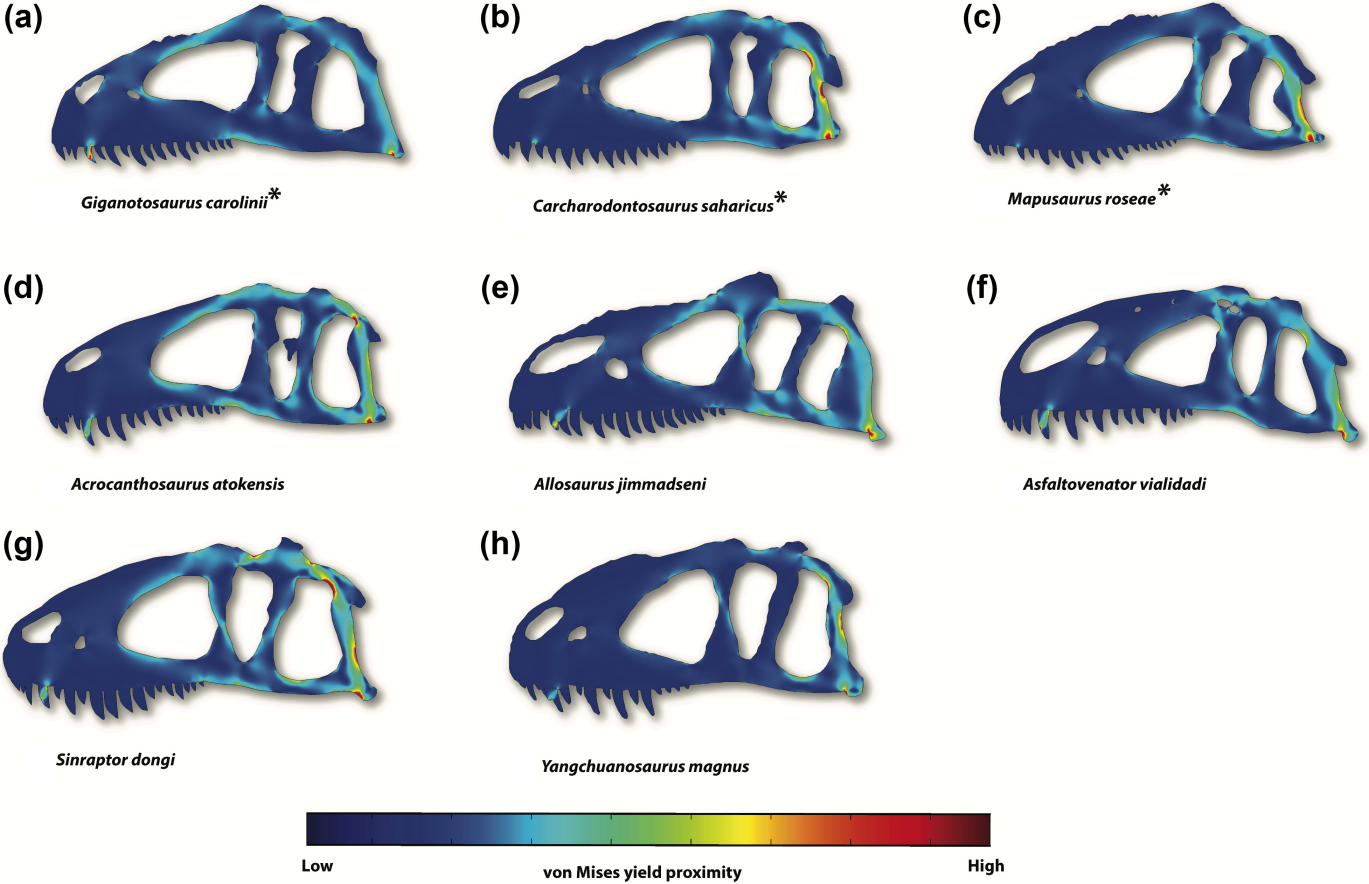
von Mises stress (megapascals) in planar models of allosauroid theropods, scaled to the length (1 m) and muscle force of adult *Tyrannosaurus rex* (USNM 555000); (*) indicates that the theropod model is a composite. Allosauroids show cranial stresses analogous to those in deep‐snouted members of Tyrannosauridae. The stresses displayed in the crania were relatively low in allosauroids, suggesting that having a deeper snout enabled allosauroids such as *Allosaurus jimmadseni* (e), *Asfaltovenator vialidadi* (f), *Sinraptor dongi* (g), and *Yangchuanosaurus magnus* (h) to exhibit low cranial stress and handle high forces. In the larger carcharodontosaurids, the level of stress magnitudes increases [*Giganotosaurus carolinii* (a), *Carcharodontosaurus saharicus* (b), *Mapusaurus roseae* (c), and *Acrocanthosaurus atokensis* (d)]. Allosauroids show high stress in the lacrimal bar, temporal complex, and quadratojugal. 6 MPa is the greatest visualized stress for the theropod crania.

**FIGURE 12:**
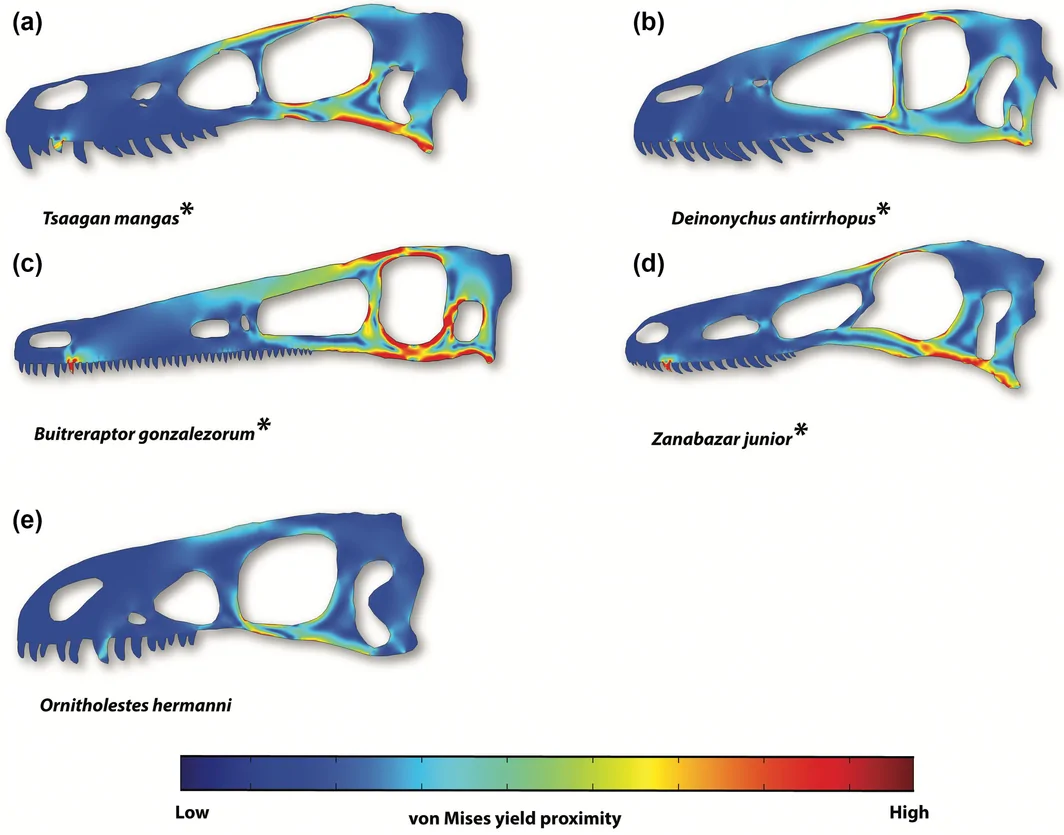
von Mises stress (megapascals) in planar models of maniraptoran theropods scaled to the length (1 m) and muscle force of adult *Tyrannosaurus rex* (USNM 555000); (*) indicates that the theropod model is a composite. The slender‐snouted unenlagiine *Buitreraptor gonzalezorum* (c) and the velociraptorine *Tsaagan mangas* (a) show very high cranial stress compared to deep‐snouted maniraptorans [*Deinonychus antirrhopus* (b) and *Ornitholestes hermanni* (e)] and the troodontid *Zanabazar junior* (d). The deep‐snouted morphology of some maniraptorans may have allowed them to resist high forces during biting. 6 MPa is the greatest visualized stress for the theropod crania.

**FIGURE 13:**
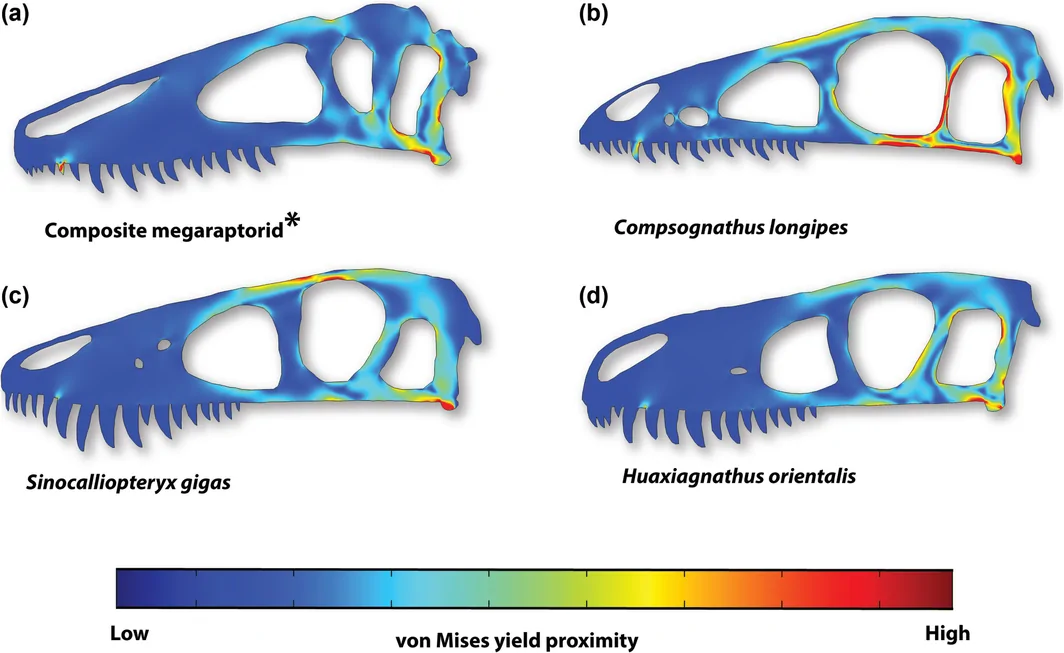
von Mises stress (megapascals) in planar models of coelurosaurian theropods with phylogenetic affinities to Tyrannosauroidea scaled to the length (1 m) and muscle force of adult *Tyrannosaurus rex* (USNM 555000); (*) indicates that the theropod model is a composite. The composite megaraptorid (a) and compsognathids show von Mises cranial stress consistent with early tyrannosauroids (e.g., nasal stress and stress occurring around the postorbital fenestra). The large compsognathids *Sinocalliopteryx gigas* (c) and *Huaxiagnathus orientalis* (d) show lower von Mises stress magnitudes than the small *Compsognathus longipes* (b). 6 MPa is the greatest visualized stress for the theropod crania.

**FIGURE 14:**
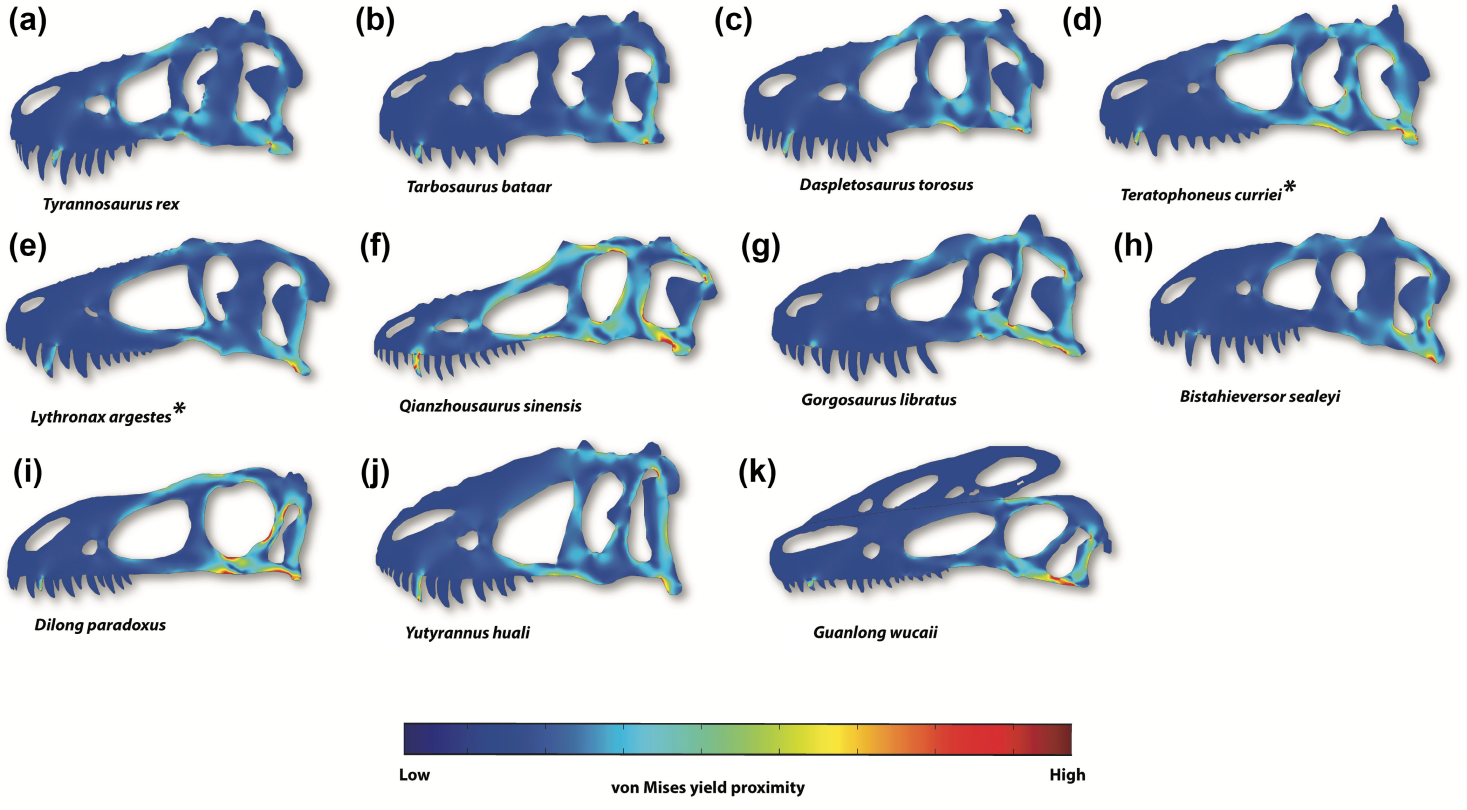
von Mises stress (megapascals) in planar models of tyrannosauroids scaled to the length (1 m) and muscle force of adult *Tyrannosaurus* (USNM 555000); (*) indicates that the theropod model is a composite. Most of the tyrannosaurids and *Bistahieversor* (h) display low levels of cranial stress except for alioramins [e.g., *Qianzhousaurus sinensis*, (f)]. The slender‐snouted alioramins show higher levels of cranial stress than deep‐snouted tyrannosaurids [e.g., *Tyrannosaurus rex* (a), *Tarbosaurus bataar* (b), *Daspletosaurus torosus* (c), *Teratophoneus curriei* (d), *Lythronax argestes* (e), and *Gorgosaurus libratus* (g)]. Small, early tyrannosauroids such as *Dilong paradoxus* (i) and *Guanlong wucaii* (k) show cranial stress magnitudes like early‐stage juvenile tyrannosaurids (e.g., *Raptorex*); low stress is present in the crest of *Guanlong*. The large proceratosaurid *Yutyrannus* (j) shows low cranial stresses and stress patterns like large, deep‐snouted tyrannosaurids. 6 MPa is the greatest visualized stress for the theropod crania.

**FIGURE 15:**
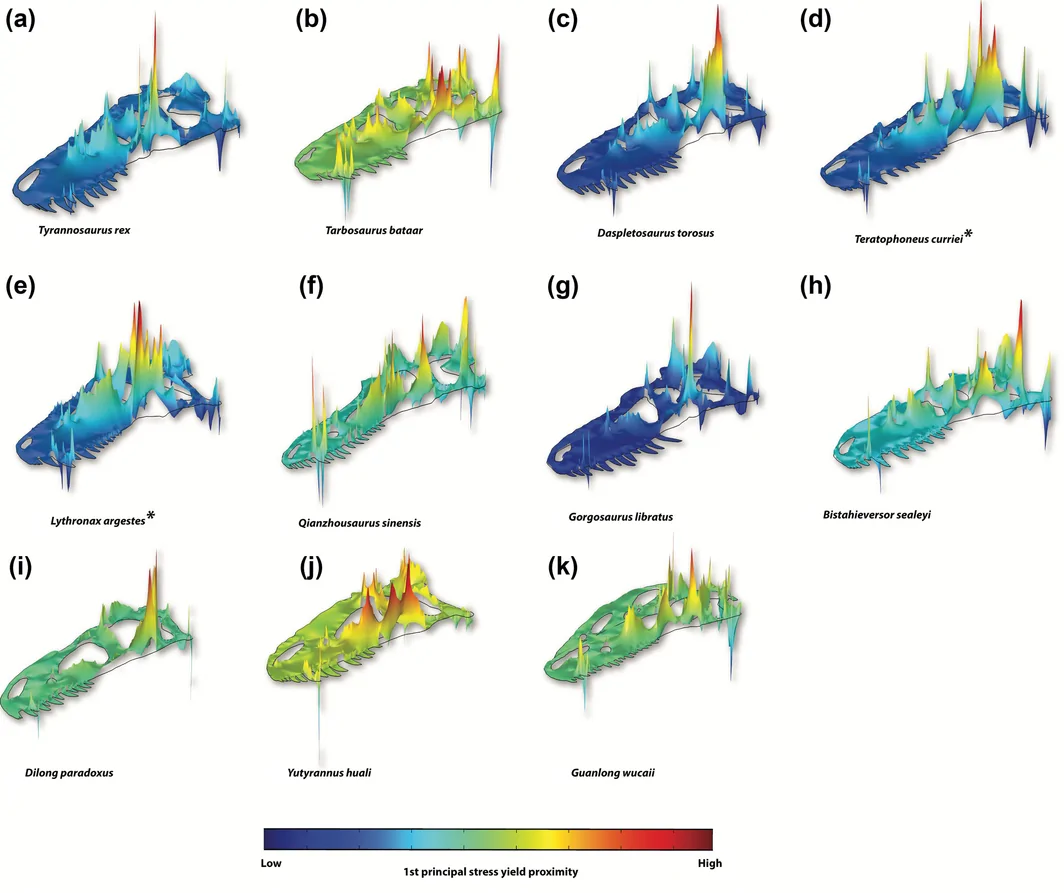
First principal stress (megapascals) in 2D planar models of tyrannosaurids; (*) indicates that the theropod model is a composite. First principal stress primarily reflects tension. Height represents the magnitude of tension, and depth represents the magnitude of compression. The peak magnitudes reflect artifacts at constraints (e.g., anterior teeth and jaw joint). The deep‐snouted tyrannosaurids *Tyrannosaurus rex* (a), *Tarbosaurus bataar* (b), *Daspletosaurus torosus* (c), *Teratophoneus curriei* (d), *Lythronax argestes* (e), and *Gorgosaurus libratus* (g) show tensile stress occurring at the maxillary‐jugal suture, lacrimals, jugal, temporal complex, and squamosal. Tyrannosaurines and *Bistahieversor sealeyi* (h) show a wide distribution of tensile stress at the crania but are in lower magnitudes than the slender‐snouted alioramine *Qianzhousaurus sinensis* (f). Both *Guanlong wucaii* (k) and *Dilong paradoxus* (i) show tensile stress occurring at the maxillary‐jugal suture, jugal, and quadratojugal. The level of tensile stress in the two early tyrannosauroids is widely distributed at the back of the crania. The large proceratosaurid, *Yutyrannus huali* (j), shows high tensile stress occurring at the maxillary‐fugal suture, jugal, nasal‐lacrimal suture, temporal complex, and quadratojugal.

**FIGURE 16:**
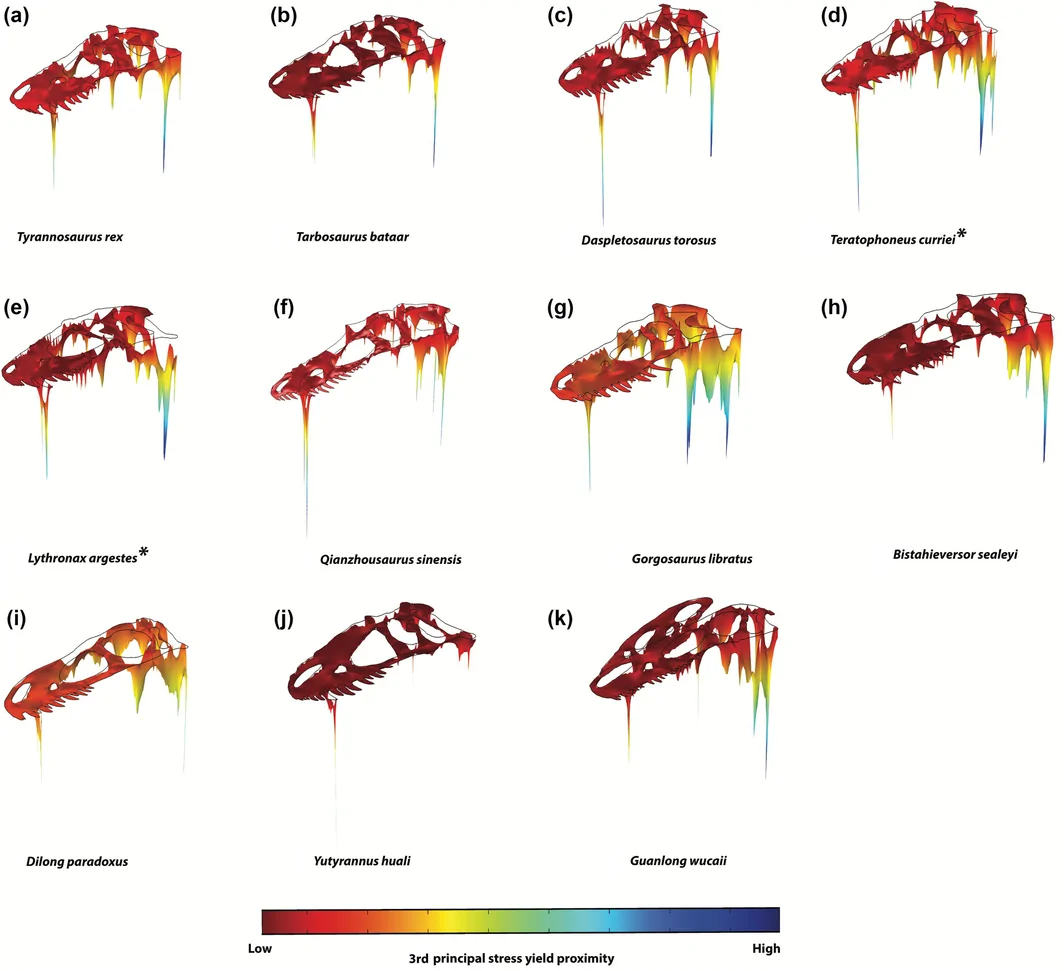
Third principal stress (megapascals) in 2D planar models of tyrannosaurids; (*) indicates that the theropod model is a composite. Third principal stress reflects compression. Depth represents the magnitude of compression. The peak magnitudes reflect artifacts at constraints (e.g., anterior teeth and jaw joint). The areas of the crania in tyrannosaurids that show peak compressive stress are analogous to the compressive stress in early tyrannosaurs. While tyrannosaurids show overall low peak compressive stress, the slender‐snouted *Qianzhousaurus sinensis* (f) and the albertosaurine *Gorgosaurus libratus* (g) show higher peak compressive stress than the deep‐snouted tyrannosaurids [*Tyrannosaurus rex* (a), *Tarbosaurus bataar* (b), *Daspletosaurus torosus* (c), *Teratophoneus curriei* (d), *Lythronax argestes* (e), and *Bistahieversor sealeyi* (h)]. The early tyrannosauroids show compressive stress occurring at the temporal complex, jugal, quadratojugal, nasals, and nasal‐lacrimal suture. The proceratosaurids *Guanlong wucaii* (k) and *Yutyrannus huali* (j) show lower compressive peak stress than the early pan tyrannosaur *Dilong paradoxus* (i). Among the small early tyrannosauroids, *Guanlong* (k) shows relatively higher peak compressive stress magnitudes than *Dilong* (i) in the posterior region of the crania.

**FIGURE 17:**
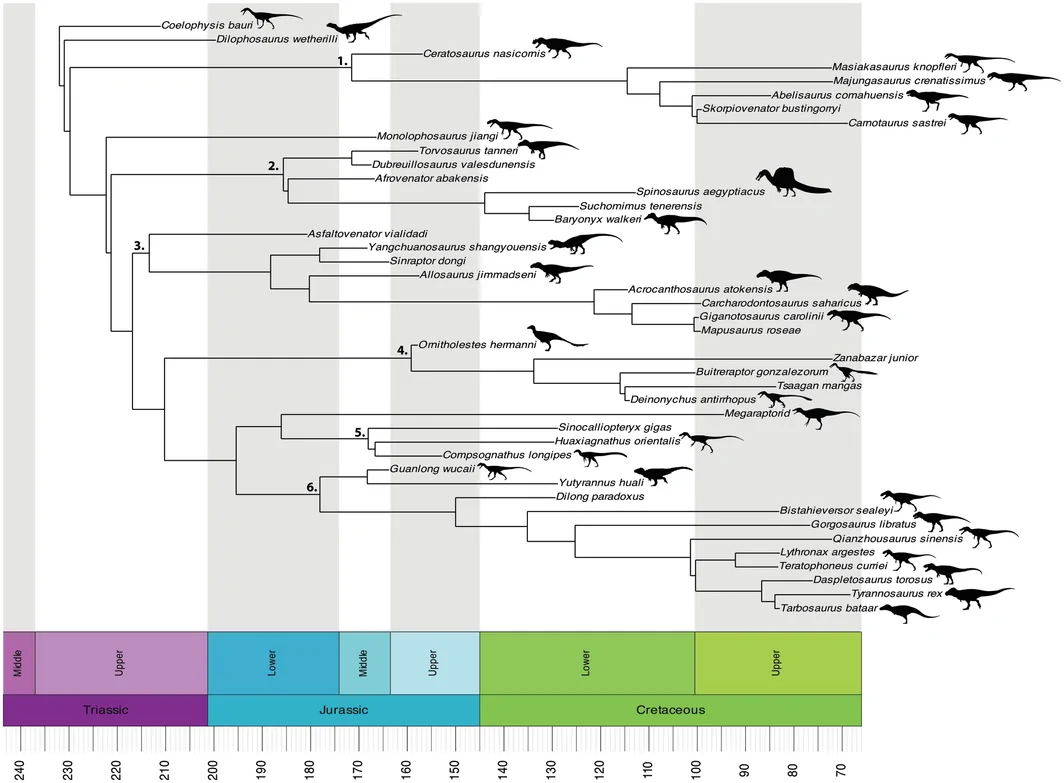
Cladogram showing the time‐scaled phylogeny of Theropoda, and the theropods used in the 2D FEA study. The theropod clades depicted include Ceratosauria (1), Megalosauroidea (2), Allosauroidea (3), Maniraptora (4), Compsognathidae (5), and Tyrannosauroidea (6). Images from Phylopic. Artist credit: Scott Hartman (*Coelophysis*, *Ceratosaurus*, *Majungasaurus*, *Carnotaurus*, *Giganotosaurus*, *Deinonychus*, *Huaxiagnathus*, *Monolophosaurus*, *Suchomimus*, *Guanlong*, *Masiakasaurus*, *Bistahieversor*), Jagged Fang Designs (*Compsognathus*, *Torvosaurus*, *Acrocanthosaurus*, *Daspletosaurus*, *Yangchuanosaurus*), Ivan Iofrida (*Spinosaurus*), Tasman Dixon (*Dilophosaurus*, *Allosaurus*, *Carcharodontosaurus*, *Yutyrannus*), and Jack Mayer Wood (*Tyrannosaurus*), Alessio Ciaffi (*Abelisaurus*), Ivan Iofrida (*Baryonyx*, *Buitreraptor*, *Gorgosaurus*), Matt Martyniuk (*Ornitholestes*, *Tarbosaurus*), T. K. Robinson (composite megaraptorid), Ashley Patch (*Qianzhousaurus*), Conty (*Tarbosaurus*), and T. Michael Keesey (*Tarbosaurus*).

**FIGURE 18:**
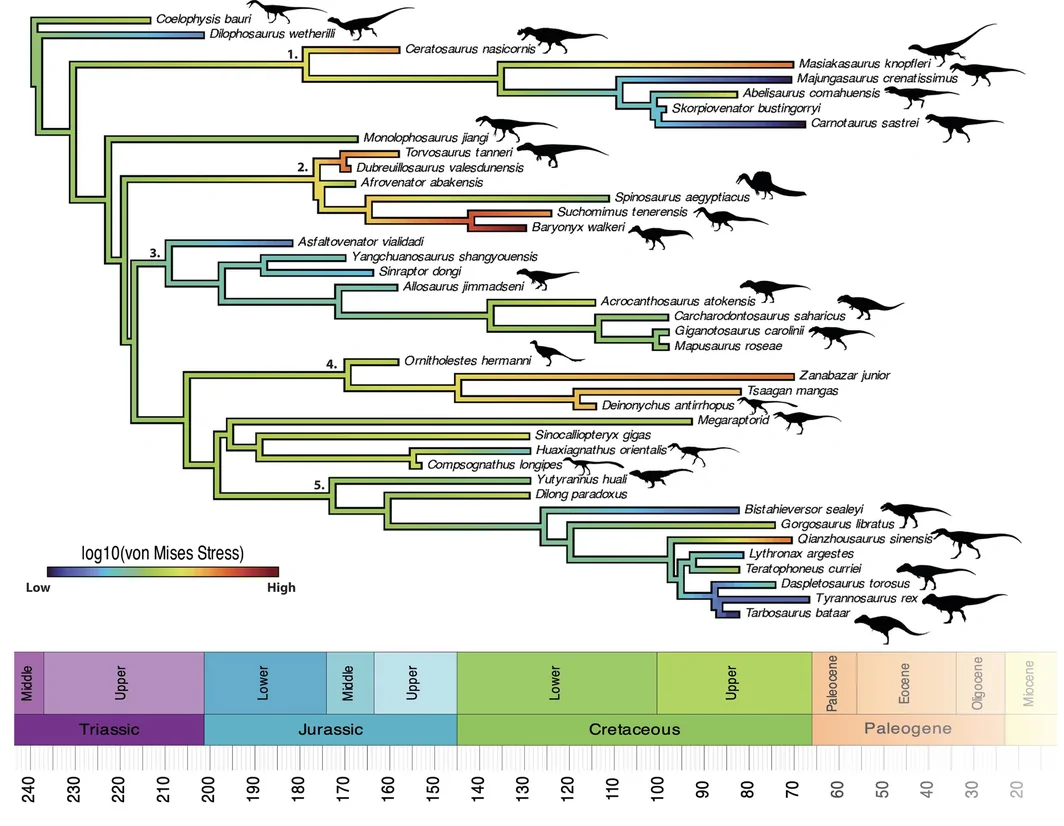
Cladogram showing the average von Mises stress present in the theropod crania and their respective theropod clades (1. Ceratosauria; 2. Megalosauroidea; 3. Allosauroidea; 4. Maniraptora; 5. Tyrannosauroidea). The ancestral condition of Tyrannosauroidea and Allosauroidea shows moderately lower von Mises stress averages than the ancestral condition of Megalosauroidea, Ceratosauria, and Maniraptora. Artist credit: Scott Hartman (*Coelophysis*, *Ceratosaurus*, *Majungasaurus*, *Carnotaurus*, *Giganotosaurus*, *Deinonychus*, *Huaxiagnathus*, *Monolophosaurus*, *Suchomimus*, *Guanlong*), Jagged Fang Designs (*Compsognathus*, *Torvosaurus*, *Acrocanthosaurus*, *Daspletosaurus*), Ivan Iofrida (*Spinosaurus*), Tasman Dixon (*Dilophosaurus*, *Allosaurus*, *Carcharodontosaurus*, *Yutyrannus*), and Jack Mayer Wood (*Tyrannosaurus*, *Masiakasaurus*), Alessio Ciaffi (*Abelisaurus*), Ivan Iofrida (*Baryonyx*, *Buitreraptor*, *Gorgosaurus*), Matt Martyniuk (*Ornitholestes*, *Tarbosaurus*), T. K. Robinson (composite megaraptorid), Ashley Patch (*Qianzhousaurus*), Conty (*Tarbosaurus*), and T. Michael Keesey (*Tarbosaurus*).

In megalosaurids, high tensile stress magnitudes are present at the maxilla near the maxillary fenestra, the lacrimal crests, and jugal (Figure S5d–f). Out of the three megalosaurids, *Dubreiullosaurus* (Figure S5f) shows the lowest tensile stress magnitudes, with *Afrovenator* (Figure S5e) having the second highest stress magnitudes, and *Torvosaurus* (Figure S5d) having the highest tensile stress magnitudes. In contrast to the tensile stress (larger megalosaurids having relatively greater tensile stress than smaller megalosaurids), the compressive stress magnitudes show an inverse with smaller megalosaurids like *Dubreiullosaurus* (Figure S6f) having relatively greater compressive stress than larger megalosaurids like *Afrovenator* (Figure S6e) and *Torvosaurus* (Figure S6d). The compressive stress of the three megalosaurids occurs in the nasals and nasal‐lacrimal connection (Figure S6d–f). This *Torvosaurus* (Figure S6d) specimen lacks preserved nasals, and stress here is, therefore, less interpretable. Spinosaurids display very high tensile stress at the jugal and maxillary‐jugal contact, with *Spinosaurus* (Figure S5a) and *Suchomimus* (Figure S5b) showing prominent tensile stress at the squamosals. High tensile stress occurs at the subnarial gap between the premaxilla and maxilla in the spinosaurids (Figure S5a–c). High compressive stress occurs at the nasals, temporal complex, and squamosals of the spinosaurids, with *Suchomimus* (Figure S6b) showing the higher compressive stress than *Baryonyx* (Figure S6c) and *Spinosaurus* (Figure S6a). *Monolophosaurus* shows moderately high tensile stress at the jugal, lacrimals, and at the temporal complex (Figure S5g). *Monolophosaurus* has compressive stress occurring in similar areas of the cranium but is higher at the temporal complex and jugal (Figure S6g).

#### 4.3.4 Allosauroids

Most of the high von Mises stresses in the allosauroids are consistently present at the quadratojugal and quadrate (Figures 6d, 7b, and 11). Additionally, high von Mises stress occurs at the lacrimals and temporal complex. The metriacanthosaurid *Sinraptor* (Figure 11g) displays high stress at the inferior region of the squamosals (sampled squamosal stress being 1.2 MPa), quadrate, quadratojugal, and the posterior portion of the postorbital (1.3 MPa). The deep‐snouted metriacanthosaurid *Yangchuanosaurus* shows predominantly low von Mises stress with moderately low stress occurring at the squamosal (0.6 MPa) and quadratojugal (Figure 11h). Among the metriacanthosaurids, *Yangchuanosaurus* shows a cranial stress average of 0.3 MPa, compared to *Sinraptor's* 1.0 MPa. Both the early‐diverging allosauroid *Asfaltovenator* (Figure 11f) and *Allosaurus* (Figures 6d and 11e) display high stress at the lacrimals (0.9 MPa = *Asfaltovenator*, 0.7 MPa = *Allosaurus*), temporal complex, quadrate, and quadratojugal. *Allosaurus* shows a von Mises stress average of 1.1 MPa, and *Asfaltovenator* has an average von Mises stress of 0.9 MPa. The large‐bodied carcharodontosaurids display stress magnitudes that are similar to those present in *Allosaurus*, with each carcharodontosaurid exhibiting high stress magnitudes in distinct areas of the crania. *Acrocanthosaurus* (Figure 11d) exhibits higher von Mises stress magnitudes in its quadratojugal, quadrate, and lacrimals than *Giganotosaurus* (Figures 7b and 11a), *Carcharodontosaurus* (Figure 11b; lacking a quadrate), and *Mapusaurus* (Figure 11c; MCF‐PVPH‐108.11 maxilla, MCF‐PVPH‐108.6 quadrate, MCF‐PVPH‐108.5 lacrimal). The latter three carcharodontosaurids exhibit lower stress magnitudes at the aforementioned areas of the crania, with stress largely encompassing the quadratojugal and quadrate (Figure 11a–c). Among the large carcharodontosaurids, *Acrocanthosaurus* shows an average cranial von Mises stress of 1.5 MPa, which is relatively greater than *Giganotosaurus* (1.2 MPa), *Carcharodontosaurus* (1.2 MPa), and *Mapusaurus* (1.3 MPa). This would indicate the large carcharodontosaurids have low cranial stress during feeding (Figures 6d, 7b, 11, S7, and S8).

The allosauroids *Asfaltovenator* (Figure S7f), *Sinraptor* (Figure S7g), *Yangchuanosaurus* (Figure S7h), *Allosaurus* (Figure S8e), and the large carcharodontosaurids (Figure S7b–d) exhibit high tensile stress magnitudes at the maxilla, maxilla‐jugal contact, jugal (except for *Giganotosaurus*; Figure S7a), lacrimal, nasal‐lacrimal contact, and temporal complex. Compressive stress magnitudes occur in consistent areas of the crania of the allosauroids such as the nasals, maxilla, temporal complex, and jugal (Figure S8). Medium‐sized allosauroids such as *Asfaltovenator* (Figure S8f), *Sinraptor* (Figure S8g), *Yangchuanosaurus* (Figure S8h), *Allosaurus* (Figure S8e) exhibit lower compressive stress magnitudes in contrast to the higher compressive stress in the crania of large‐bodied carcharodontosaurids (e.g., *Giganotosaurus*; Figure S8a and *Acrocanthosaurus*; Figure S8d).

#### 4.3.5 Maniraptorans

Dromaeosaurids show high von Mises stress magnitudes that are present in the nasals and bones encompassing the orbit (Figures 5d–e and 12a–c). The Mongolian dromaeosaurid *Tsaagan* shows high von Mises stress present at the dorsal portion of the frontals and lacrimals (sampled lacrimal stress = 0.8 MPa; Figures 5e and 12a). The unenlagine dromaeosaurid *Buitreraptor* (Figure 12c) shows a von Mises stress distribution similar to *Tsaagan* (Figures 5e and 12a) such as the frontal and nasals (sampled nasal stress of *Buitreraptor* = 2.1 MPa) but are higher in magnitude, especially in bones surrounding the orbital fenestra (e.g., frontal, quadrate, and ventral portion of postorbital) and the nasals. *Deinonychus* (Figures 5d and 12b) shows von Mises stress magnitudes occurring at areas of the cranium similar to *Tsaagan* (Figures 5e and 12a) and *Buitreraptor* (Figure 12c) but are lower in magnitude. In *Deinonychus*, moderately low von Mises stress occurs in YPM 5232's nasals (1.3 MPa), posterior jugal (2.1 MPa), and postorbital (1.8 MPa) and AMNH FARB 5210's squamosal (1.8 MPa) and jugal. Small portions of high von Mises stress occur in YPM 5232's mirrored lacrimal. The troodontid *Zanabazar* (Figure 12d) shows low von Mises stress magnitudes present in the maxilla‐jugal suture (1.1 MPa). High von Mises stress is highly prevalent in the quadrate. *Ornitholestes*, a basal maniraptoran, shows primarily low cranial stress with the nasals (0.8 MPa) and ventral portion of the jugal (1.5 MPa) showing moderately low von Mises stress magnitudes (Figure 12e). The unenlagiine *Buitreraptor* shows relatively greater average von Mises stress average (3.1 MPa) than the dromaeosaurids *Deinonychus* (1.7 MPa) and *Tsaagan* (1.7 MPa), the troodontoid *Zanabazar* (1.9 MPa), and the maniraptoriform *Ornitholestes* (1.4 MPa) (Figures 5d–e, 12, S9, and S10).

The dromaeosaurids show moderately low tensile stress in the crania with prominent peaks occurring in the jugals (except for *Tsaagan*; Figure S9a), lacrimals, and temporal complex (except for *Deinonychus*; Figure S9b). *Tsaagan* and *Deinonychus* show greater peak tensile stresses than the tube‐snouted unenlagine *Buitreraptor* (Figure S9c). The dromaeosaurids show low compressive stress in the crania, with moderately high peak stresses occurring in the nasal‐lacrimal connection and jugals (Figure S10a–c). The Mongolian dromaeosaurid *Tsaagan* (Figure S10a) shows greater compressive peak stress than *Deinonychus* (Figure S10b) and *Buitreraptor* (Figure S10c). The troodontid *Zanabazar* (Figure S9d) shows tensile stress in analogous regions of the cranium similar to dromaeosaurids but are relatively lower in magnitude than *Deinonychus* (Figure S9b) and *Tsaagan* (Figure S9a). Moderately low compressive stress occurs in the cranium of *Zanabazar*, with prominent peaks present in the nasals and jugal of the troodontid (Figure S10d). The basal maniraptoran *Ornitholestes* shows low tensile stress magnitudes with prominent peaks occurring at the lacrimal and jugal (Figure S9e). Low compressive stress is present in the cranium of *Ornitholestes* with prominent peaks occurring in the nasals, temporal complex, and jugals (Figure S10e).

#### 4.3.6 Clades proximate to Tyrannosauroidea

The composite megaraptorid shows a von Mises stress distribution similar to the tyrannosauroids, with stresses showing at the nasals (0.9 MPa) and nasal‐lacrimal connection (1.3 MPa) of *Megaraptor* and *Murusraptor* (Figure 13a). A major difference between the composite megaraptorid and the tyrannosauroids is moderately high von Mises stress occurring at the ventral portion of the quadrate of *Orkoraptor* (Figure 13a). The composite megaraptorid shows an average von Mises cranial stress of 1.3 MPa. The small compsognathid *Compsognathus* shows prominent high von Mises stress at the postorbital, temporal complex, nasals, dorsal border of the postorbital fenestra, and the ventral portion of temporal complex and quadratojugal (Figure 13b). Larger compsognathids such as *Sinocalliopteryx* (Figure 13c) and *Huaxiagnathus* (Figure 13d) show von Mises stress that occurs at similar regions of the crania like *Compsognathus* (Figure 13b) but is lower in magnitude. The large compsognathid *Sinocalliopteryx* (1.5 MPa) and the small *Compsognathus* (1.5 MPa) show relatively high von Mises stress averages than the deeply robust *Huaxiagnathus* (1.1 MPa), because of the former two having relatively slender crania (Figures 13, S11, and S12).

The composite megaraptorid displays moderately low tensile stress at the maxilla from *Megaraptor* and postorbital from *Murusraptor* (Figure S11a). The composite megaraptorid shows moderately low compressive stress that occurs in the nasals (*Megaraptor*) and temporal region (*Murusraptor*; Figure S12a). The compsognathids show moderately low tensile stress in the crania with prominent peaks occurring at the maxilla‐jugal connections, nasal‐lacrimal connections, maxillae, and temporal complex (Figure S11b). The small compsognathid *Compsognathus* shows greater tensile peak stresses than the larger compsognathids (*Sinocalliopteryx* and *Huaxiagnathus*; Figure S11c,d, respectively). The compsognathids show low compressive stress in the crania, with prominent peak stresses occurring at the nasals, lacrimals, temporal complex, and jugals (Figure S12b–d). Similar to the comparative tensile stresses, *Compsognathus* (Figure S12b) shows greater peak compressive stresses in the jugal than *Sinocalliopteryx* (Figure S12c) and *Huaxiagnathus* (Figure S12d).

### 4.4 Tyrannosauroids

Among the basal tyrannosauroids (Figures 7 and 14, 15, 16), *Dilong* (Figure 14i) displays higher von Mises distributions and averages than the proceratosaurids, *Yutyrannus* (Figure 14j) and *Guanlong* (Figure 14k). In contrast to the qualitative von Mises stress distributions, the three basal tyrannosauroids differ in von Mises cranial stress averages with *Dilong* having a von Mises stress average of 1.4 MPa, *Yutyrannus* having a von Mises stress average of 1.2 MPa, and *Guanlong* having an average von Mises stress of 0.3 MPa. Low stress occurs at the nasal crest of *Guanlong* (0.002 MPa) and rugose nasals of *Yutyrannus* (0.2 MPa). In the basal tyrannosauroids, high von Mises stress magnitudes are present at the ventral portion of the squamosals, the ventral portion of the quadrate, and the temporal complex, the posterior side of the postorbital, the quadratojugal, and quadrate (Figure 14i–k). The slender‐snouted alioramin *Qianzhousaurus* exhibits high von Mises stress magnitudes that are widely distributed across the cranium, with an average von Mises stress value of 1.9 MPa, indicating the alioramin's cranium may not be adapted for resisting high forces (Figure 14f) compared to deep‐snouted tyrannosaurids. Deep‐snouted tyrannosaurids proper and their large sister taxon *Bistahieversor* exhibit notably low von Mises stress magnitudes (average von Mises stress = 0.9 MPa), with *Bistahieversor* showing predominantly low stress at the nasals (0.4 MPa) and small margins of stress occurring at the ventral portion of the quadrate and squamosal bar (1.6 MPa; Figure 14h). The deep‐snouted tyrannosaurines *Lythronax* (average von Mises stress = 1.0 MPa; Figure 14e), *Daspletosaurus* (average von Mises stress = 1.1 MPa; Figure 14c), *Teratophoneus* (average von Mises stress = 1.3 MPa; Figure 14d; UMNH VP 16690‐1, mirrored right jugal of BYU 8120), and *Tarbosaurus* (average von Mises stress = 0.8 MPa; Figure 14b) show moderately low stress concentrations at the squamosal, dorsal region of the jugal, and lateral region of the quadratojugal (Figure 14). Among the four tyrannosaurines, *Teratophoneus* shows the highest von Mises stress, and *Tarbosaurus* the lowest. The albertosaurine *Gorgosaurus* (average von Mises stress = 1.4 MPa) shows a von Mises stress distribution similar to the deep‐snouted tyrannosaurines, with the exception of low stress magnitudes occurring at the temporal complex and the ventral portion of the quadratojugal (Figure 14g). The cranium of the adult *Tyrannosaurus* specimen (AMNH FARB 5027) exhibits primarily low von Mises stress (average von Mises stress = 0.9 MPa), with low stress concentrations occurring in the nasals (0.7 MPa), nasal‐lacrimal connection (1.3 MPa), posterior jugal (0.5 MPa), and the ventral region of the squamosal bar (1.2 MPa), similar to USNM 555000, another adult *Tyrannosaurus* specimen (Figures 4a, 7a, and 14a).

The crania of other basal tyrannosauroids display moderately low tensile stress (Figure 15i–k). Both proceratosaurids, *Guanlong* (Figure 15k) and *Yutyrannus* (Figure 15j), show peak tensile stress at the ventral portions of the cranium, with prominent peak stress occurring at the maxilla and jugal, with *Yutyrannus* having prominent peak stress in the squamosals (Figure 15j) than *Guanlong* (Figure 15k). *Dilong's* cranium shows low peak tensile stresses at the maxilla, lacrimal, and temporal complex, with high peak stress occurring at the ventral portion of the jugal (Figure 15i). Similar to more derived tyrannosauroids, the three basal tyrannosauroids show peak compressive stress occurring at aforementioned regions of the crania, with *Dilong* (Figure 16i) and *Guanlong* (Figure 16k) showing higher peak stress magnitudes at the temporal complex, lacrimal crest, and ventral region of the jugal than the larger *Yutyrannus* (Figure 16j). *Bistahieversor* shows moderately low tensile stress in its cranium, with peak stresses occurring at the maxilla, ventral region of the jugal, nasals, and quadratojugal (Figure 15h). *Bistahieversor* shows similar compressive stress occurring in accordance with other deep‐snouted tyrannosaurines, with a prominent peak stress occurring at the jaw joint (Figure 16h).

The alioramin *Qianzhousaurus* displays moderately low tensile stress at the jugal with higher magnitudes in distal regions of the cranium such as the temporal complex, squamosals, and near the jaw joint (Figure 15f). Similar to other tyrannosauroids, compressive stress occurs in the nasals, lacrimals, and temporal complex of *Qianzhousaurus*, with prominent peak stress occurring at the ventral portion of the jugal and quadrate (Figure 16f).

Among the deep‐snouted tyrannosaurines (*Tarbosaurus* Figure 15b; *Daspletosaurus* Figure 15c; *Teratophoneus* Figure 15d UMNH VP 16690‐1, BYU 8120; and *Lythronax* Figure 15e), *Tarbosaurus* shows the highest tensile stress with prominent peak stress occurring at the ventral jugal and quadrate (similar to other deep‐snouted tyrannosaurines). The deep‐snouted tyrannosaurines show tensile stress occurring at the maxillae, lacrimals, and squamosals (Figure 15a–e). The deep‐snouted tyrannosaurines exhibit compressive stress occurring consistently at the aforementioned regions (nasals, squamosals, and temporal complex) of the crania, with the exception of the *Lythronax* specimen which lacks a complete braincase (Figure 16a–e). Being the same body size as *Daspletosaurus*, the albertosaurine *Gorgosaurus* exhibits higher tensile stress at the temporal complex and at the jugal and squamosal (Figure 15g). *Gorgosaurus* also exhibits higher compressive stress at the temporal complex with greater magnitudes present at the squamosals and at the jugal (Figure 16g).

The adult *Tyrannosaurus* exhibits moderately low tensile stress at the squamosal and maxilla, with higher tensile stress occurring at the jugal (Figure 15a). An adult *Tyrannosaurus* shows consistently low compressive stress at the temporal complex, nasals, jugal, squamosal, and near the lacrimals, as seen with other tyrannosauroids (Figure 16a).

## 5 ANCESTRAL‐STATE CRANIAL STRESSES AT SAMPLED LOCATIONS

### 5.1 Maximum likelihood ancestral character state estimates

The maximum likelihood analysis was the total summation and average of the sampled cranial stress points of the studied theropods (Figure 17 and Table S3). For the maximum likelihood analysis, the theropods *Buitreraptor* and *Guanlong* were excluded, because they were viewed as outliers of the log‐transformed von Mises stress (standard deviation = 0.11). *Guanlong's* log(von Mises stress) was 5.5 MPa, which was about 4–5 standard deviations away from the lowest log(von Mises stress). *Buitreraptor's* log(von Mises stress) was 7.5 MPa, about 10 standard deviations away from the highest log(von Mises stress). While *Buitreraptor* and *Guanlong* were excluded from the maximum likelihood analysis, the two theropods were included in the squared‐change parsimony analysis.

The average von Mises stress for Theropoda was 2.0 MPa. The ancestral condition of Ceratosauria has an average von Mises stress value of 1.3 MPa, with Abelisauridae's at 1.0 MPa. Ceratosauria's ancestral condition has a relatively lower von Mises stress average than the ancestral condition of Avetheropoda (2.8 MPa), a clade of mostly derived theropods. The total von Mises stress average for Megalosauroidea's ancestral condition is 1.7 MPa, with Spinosauridae's ancestral condition (1.8 MPa) having a relatively greater von Mises stress average than Megalosauridae's ancestral condition (1.6 MPa). The ancestral condition of Allosauroidea has an average von Mises value of 1.1 MPa, with Carcharodontosauridae having an average stress value of 1.3 MPa. The ancestral condition of Coelurosauria has a value of 2.8 MPa with the ancestral condition of Maniraptoriformes (7.5 MPa) having a relatively greater von Mises stress average than the ancestral conditions of coelurosaurs proximate to tyrannosauroids (1.3 MPa) and Tyrannosauroidea (1.2 MPa). The ancestral condition of Tyrannosauridae shows an average stress value of 1.1 MPa.

### 5.2 Squared‐change parsimony ancestral character state estimates

In contrast to the maximum likelihood analysis which used an average of the sampled cranial points and overall cranial performance (Figure 17), the square‐change parsimony analysis (Figures [Link], [Link]) emphasized specific cranial regions (e.g., nasals, jugal, and nasal‐lacrimal suture) of the studied theropod clades. The sampled cranial points were selected not only for evaluating stress performance, but to infer functional performance during feeding (Ma et al., 2022; Salcido & Polly, 2025). Qualitatively, the ancestral nodes of the theropod clades show relatively low stress magnitudes. To visually highlight and more easily compare stress distributions among clades, we defined the color scale boundaries on the trees (Figures [Link], [Link]) such that the highest and lowest magnitudes on each tree were assigned to their own color bins, with the remaining color bins equally divided among the range bounded by the second highest and lowest von Mises stress averages in Mesquite. An example would be setting the boundaries for the nasal‐lacrimal suture (Figure S15), using the theropods *Guanlong* and *Dubreuillosaurus* with the second lowest and highest stress values, 0.2 and 2.7 MPa, respectively, to define the color value bins inclusive of all sampled theropods except for *Yangchuanosaurus* and *Buitreraptor*, which had the overall lowest and highest values, respectively, and are assigned their own bins to allow for the ranges of values of the other taxa to be more easily visually distinguished.

The nasals (Figure S13) in Theropoda show a von Mises stress value of 1.0 MPa, indicative of low stress. Early non‐averostran theropod clades (e.g., *Coelophysis* and *Dilophosaurus*) show low nasal stress values of 1.3 and 0.2 MPa. Because Ceratosauria consists of differing clades (Ceratosauridae, Noasauridae, and Abelisauridae), the overall theropod clade shows a low nasal stress value of 0.9 MPa, with Abelisauridae showing the lowest overall nasal stress (0.8 MPa). Tetanuran theropods (e.g., Megalosauroidea, Allosauroidea, and Coelurosauria) show relatively lower nasal stress (0.5 MPa) than other basal theropod clades. Within Megalosauroidea, megalosaurids show relatively lower nasal stress (0.9 MPa) than spinosaurids (1.4 MPa). Allosauroidea shows relatively lower nasal stress (0.5 MPa) than Coelurosauria (1.4 MPa). The large carcharodontosaurids show relatively greater nasal stress (1.1 MPa) than smaller allosauroids (e.g., *Allosaurus*, 0.6 MPa; Metriacanthosauridae, 0.5 MPa). Maniraptoriformes show relatively greater nasal stress (2.2 MPa) than tyrannosauroids (0.7 MPa) and clades proximate to Tyrannosauroidea (0.9 MPa). Among Maniraptoriformes, dromaeosaurids show relatively greater nasal stress (9.8 MPa) than troodontids (1.1 MPa). Within Tyrannosauroidea, the crested proceratosaurids show relatively lower nasal stress (0.3 MPa) than derived pantyrannosaurs (1.0 MPa; e.g., *Gorgosaurus* and Tyrannosaurinae). The deep‐snouted tyrannosaurines show relatively lower nasal stress (1.0 MPa) than *Gorgosaurus* (1.1 MPa) and *Qianzhousaurus* (1.3 MPa).

The sampled lacrimal‐jugal stresses (Figure S14) vary in stress magnitude than the sampled nasal stresses, with Theropoda showing a relatively low lacrimal‐jugal stress value of 0.7 MPa compared to the nasals (1.0 MPa). Large non‐averostran theropods (e.g., *Dilophosaurus*) show relatively greater lacrimal‐jugal stress (1.0 MPa) than *Coelophysis* (0.5 MPa). Ceratosauria shows a relatively low lacrimal‐jugal stress of 1.1 MPa, with abelisaurids showing consistently low lacrimal stress (0.9 MPa). In contrast to the nasal stress, tetanuran theropods show relatively greater lacrimal‐jugal stress (1.2 MPa) than non‐averostran theropods. Consistently within Megalosauroidea (lacrimal‐jugal stress = 1.1 MPa), spinosaurids show relatively greater lacrimal‐jugal stress (1.3 MPa) than megalosaurids (1.0 MPa). Like the nasal stress of Allosauroidea (lacrimal‐jugal stress = 1.1 MPa), the large carcharodontosaurids show relatively greater lacrimal‐jugal stress (0.9 MPa) than smaller allosauroids (Metriacanthosauridae lacrimal‐jugal stress = 0.7 MPa, *Allosaurus* lacrimal‐jugal stress = 0.7 MPa). Coelurosauria shows a lacrimal‐jugal stress of 2.0 MPa with Maniraptoriformes, specifically dromaeosaurids (lacrimal‐jugal stress = 1.3 MPa), showing relatively greater lacrimal‐jugal stress (3.0 MPa) than tyrannosauroids (1.1 MPa) and clades proximate to Tyrannosauroidea (1.2 MPa). Within Tyrannosauroidea, proceratosaurids show similar lacrimal‐jugal stress (0.9 MPa) to pantyrannosaurs (0.9 MPa). The deep‐snouted tyrannosaurines (lacrimal‐jugal stress = 1.0 MPa) and *Gorgosaurus* (lacrimal‐jugal stress = 1.1 MPa) show relatively lower lacrimal‐jugal stress than *Qianzhousaurus* (1.4 MPa).

The nasal‐lacrimal suture (Figure S15) is a connection between the nasals and lacrimal of the studied theropod and may have likely been akinetic during feeding (Cost et al., 2020; Hurum & Sabath, 2003). Theropoda consistently shows a moderately low nasal‐lacrimal stress value of 1.5 MPa. Small early theropods (*Coelophysis*, nasal‐lacrimal stress = 1.6 MPa) show relatively greater nasal‐lacrimal stress than *Dilophosaurus* (1.2 MPa). In Ceratosauria (nasal‐lacrimal stress = 1.8 MPa), *Ceratosaurus* shows relatively greater nasal‐lacrimal stress (2.4 MPa) than Abelisauridae (1.2 MPa). Within Megalosauroidea (nasal‐lacrimal stress = 1.8 MPa), spinosaurids show relatively lower nasal‐lacrimal stress (1.6 MPa) than megalosaurids (2.3 MPa). Consistently, allosauroids show relatively lower nasal‐lacrimal stress (1.2 MPa) than coelurosaurs (1.7 MPa). Within Allosauroidea, metriacanthosaurids (nasal‐lacrimal stress = 0.8 MPa) and *Allosaurus* (1.0 MPa) show relatively lower nasal‐lacrimal stress than carcharodontosaurids (1.4 MPa). Within Maniraptoriformes, dromaeosaurids show relatively greater nasal‐lacrimal stress (7.3 MPa) than *Zanabazar* (1.5 MPa). Coelurosaurs proximate to tyrannosauroids (e.g., compsognathids = 1.5 MPa and composite megaraptorid = 1.3 MPa) show relatively greater nasal‐lacrimal stress (1.4 MPa) than Tyrannosauroidea (1.1 MPa). Within Tyrannosauroidea, Proceratosauridae shows relatively lower nasal‐lacrimal stress (0.8 MPa) than Pantyrannosauria (1.2 MPa). The deep‐snouted tyrannosaurines (nasal‐lacrimal stress = 1.2 MPa) and *Gorgosaurus* (1.1 MPa) show relatively lower nasal‐lacrimal stress than *Qianzhousaurus* (1.5 MPa).

Two regions of the jugal (inferior portion, underneath the orbit, Figure S16; posterior portion, Figure S17) were selected as proxies for 2D attachments for the pterygoid muscles (Johnson‐Ransom et al., 2024). In Theropoda, the stress value for the jugal is 1.6 MPa (inferior) and 1.5 MPa (posterior), indicative of relatively low jugal (or pterygoid) stress during biting. The early theropod *Coelophysis* shows relatively low jugal stress of 1.9 MPa for the inferior and posterior regions of the jugal. Ceratosauria shows relatively low jugal stress values of 1.4 MPa (inferior) and 1.1 MPa (posterior), with Abelisauridae showing the relatively lowest jugal stress (0.98 MPa, inferior and 1.0 MPa, posterior). Both the inferior and posterior regions of the jugal in Megalosauridae show similar values of 1.4 MPa, with Spinosauridae showing jugal stress values of 1.6 MPa (inferior) and 1.9 MPa (posterior), which are relatively higher jugal stress values than megalosaurids'. Similar to Megalosauridae, Allosauroidea show similar stress values of 1.1 MPa in the ventral and posterior regions of the jugal. Coelurosauria shows jugal stress values of 3.0 MPa (inferior) and 2.4 MPa (posterior). Maniraptoriformes show relatively higher jugal stress values of 5.1 MPa (inferior) and 3.7 MPa (posterior) than tyrannosauroids (1.5 MPa = inferior and posterior) and coelurosaurs proximate to Tyrannosauroidea (1.3 MPa = inferior, 1.9 MPa = posterior). Proceratosaurids show relatively lower jugal stress (0.94 MPa = inferior and 0.97 MPa = posterior) than Pantyrannosauria (1.5 MPa = inferior and 1.6 MPa = posterior). Deep‐snouted tyrannosaurines (1.3 MPa) and *Gorgosaurus* (1.4 MPa) show relatively lower stress in the inferior region of the jugal than *Qianzhousaurus* (1.9 MPa), although albertosaurines show relatively greater stress in the posterior region of the jugal (2.7 MPa) than deep‐snouted tyrannosaurines (1.2 MPa) and *Qianzhousaurus* (1.4 MPa).

Similar to the nasal‐lacrimal suture, the maxilla‐jugal sutural points (Figure S18) of the studied theropods were selected to evaluate possible akinesis in the crania during feeding (Cost et al., 2020; Rayfield, 2004). Theropoda shows relatively low maxillary‐jugal stress (0.7 MPa). Early theropods such as *Coelophysis* and *Dilophosaurus* show relatively low maxillary‐jugal stress values of 0.3 and 1.4 MPa, respectively. Ceratosauria shows relatively low maxillary‐jugal stress (1.1 MPa), while Abelisauridae shows the overall relatively lowest maxillary‐jugal stress (0.8 MPa). Tetanurae shows relatively greater maxillary‐jugal stress (1.3 MPa) than ceratosaurs. Megalosauroidea shows a maxillary‐jugal stress of 1.2 MPa, with megalosaurids showing relatively greater maxillary‐jugal stress (1.2 MPa) than spinosaurids (1.1 MPa). Allosauroidea shows a maxillary‐jugal stress value of 1.2 MPa, with carcharodontosaurids showing relatively greater stress (1.5 MPa) than smaller allosauroids (metriacanthosaurids = 0.9 MPa, *Allosaurus* = 0.9 MPa). Maniraptoriformes show relatively greater maxillary‐jugal stress (3.1 MPa) than coelurosaurian clades proximate to Tyrannosauroidea (1.1 MPa) and Tyrannosauroidea (1.0 MPa). Proceratosaurids showing relatively greater maxillary‐jugal stress (1.2 MPa) than Pantyrannosauria (0.9 MPa). Deep‐snouted tyrannosaurines (1.3 MPa) and *Gorgosaurus* (0.5 MPa) show relatively lower maxillary‐jugal stress than *Qianzhousaurus* (2.3 MPa).

The stress point in the squamosals (Figure S19) of the studied theropods was selected because the squamosal bar is an attachment site for the *M. adductor mandibulae externus superficialis* (Johnson‐Ransom et al., 2024). Theropoda shows a squamosal stress of 1.5 MPa. The early theropod *Dilophosaurus* shows a relatively greater squamosal stress (2.1 MPa) than *Coelophysis* (1.1 MPa). Ceratosauria shows a squamosal stress of 1.9 MPa with Abelisauridae showing a relatively lower squamosal stress of 1.9 MPa than *Ceratosaurus* (2.1 MPa). Tetanurae shows a squamosal stress of 2.0 MPa. Within Megalosauroidea (squamosal stress = 2.5 MPa), the megalosaurids show a relatively greater squamosal stress (3.4 MPa) than spinosaurids (1.6 MPa). The allosauroids show a squamosal stress value of 1.9 MPa, relatively lower than Coelurosauria (2.2 MPa). Within Maniraptoriformes (squamosal stress = 2.5 MPa), Dromaeosauridae shows a squamosal stress of 1.0 MPa. Within Tyrannosauroidea (squamosal stress = 1.8 MPa), proceratosaurids show a relatively lower squamosal stress (1.5 MPa) than Pantyrannosauria (1.9 MPa). Deep‐snouted tyrannosaurines (1.5 MPa) and *Gorgosaurus* (1.6 MPa) consistently show relatively lower squamosal stress than *Qianzhousaurus* (2.4 MPa).

The postorbital‐lacrimal suture (Figure S20) was selected as another akinetic cranial suture during feeding (Hurum & Sabath, 2003; Rayfield, 2004) with Theropoda showing a postorbital‐lacrimal stress of 1.8 MPa. With non‐averostran theropods, *Dilophosaurus* shows a postorbital‐lacrimal stress of 0.2 MPa and *Coelophysis* shows a postorbital‐lacrimal stress of 2.2 MPa. Within Ceratosauria (postorbital‐lacrimal stress = 3.3 MPa), abelisaurids show a relatively lower postorbital‐lacrimal stress (1.8 MPa) than *Ceratosaurus* (5.2 MPa). Within Megalosauroidea (postorbital‐lacrimal stress = 2.0 MPa), megalosaurids have relatively greater postorbital‐lacrimal stress (2.1 MPa) than spinosaurids (1.9 MPa). Allosauroidea shows a postorbital‐lacrimal stress of 1.6 MPa, relatively lower than Coelurosauria (3.3 MPa). Maniraptoriformes show a relatively greater postorbital‐lacrimal stress (5.3 MPa) than tyrannosauroids (1.6 MPa) and coelurosaur clades proximate to Tyrannosauroidea (2.2 MPa). Consistently, proceratosaurids show a relatively greater postorbital‐lacrimal stress (1.0 MPa) than Pantyrannosauria (1.5 MPa). Within Tyrannosauridae, deep‐snouted tyrannosaurines (1.5 MPa) and *Gorgosaurus* (1.3 MPa) have relatively lower postorbital‐lacrimal stress than *Qianzhousaurus* (2.8 MPa).

### 5.3 Allometric scaling of stress

A regression of log‐transformed von Mises stress averages and actual cranium lengths results in an *R* ^2^ value of 0.117 with a negative slope of −0.0686, indicating essentially no correlation between cranium length (a proxy for body size) and relative von Mises stress. Among the small theropods (up to 300 kg), *Compsognathus* and *Coelophysis* have lower von Mises stress than the longer‐skulled unenlagine *Buitreraptor* and tyrannosauroid *Guanlong*. Among the small tyrannosauroids, the proceratosaurid *Guanlong* has a longer cranium than *Dilong* but relatively a greater von Mises stress average. The abelisaurs (*Carnotaurus* and *Majungasaurus*) have relatively lower von Mises stress averages than medium‐sized tyrannosauroids (*Teratophoneu*s and *Qianzhousaurus*) and spinosaurids (e.g., *Baryonyx*). The larger tyrannosaurids (*Tyrannosaurus* and *Daspletosaurus*) have lower stress than other large theropods with longer skulls, such as *Spinosaurus* and *Giganotosaurus*. Among the large tyrannosauroids, the deep‐snouted *Bistahieversor* and *Tarbosaurus* have relatively lower cranium lengths and lower von Mises stress averages than a large *Tyrannosaurus*.

## 6 DISCUSSION

### 6.1 What we can and cannot infer from 2D FEA results for theropod crania

The regional validation of 2D FEA from 3D models supports its utility to test the cranial performance of theropods and tentatively to infer their diet and relative feeding capabilities (Cuff & Rayfield, 2013; Ma et al., 2022; Rayfield, 2011; Tseng, 2013; Tseng & Flynn, 2015; Wroe et al., 2008). There are caveats to using 2D versus 3D models (Rayfield, 2011). With 2D models, we are limited to a specific plane of the crania, in this case the lateral view. Some theropod crania (e.g., megaraptorids and *Deinonychus*) are incomplete, leaving much of the volume to be reconstructed and high‐fidelity 3D FEA impossible—although the side of the cranium is well‐represented for 2D approximation. The 2D models show some greater stress magnitudes in some areas of the crania, such as high peak stress present at the temporal complex and the nasal‐lacrimal connection of *Raptorex* and high stress at the squamosals of *Tyrannosaurus* (USNM 555000). The 3D models show more realistic stress magnitudes in the same areas of the crania (e.g., temporal complex, quadrate, and nasal‐lacrimal connection).

### 6.2 Addressing the hypotheses

The hypotheses delineate structural performance of theropod crania at standardized lengths, muscle forces, and bite locations. Qualitatively, the von Mises results show the crania of the subadult eutyrannosaurian, *Raptorex*, and slender‐snouted *Qianzhousaurus* having higher cranial stress than deep‐snouted tyrannosauroids (Hypothesis 1).

The same can also explain spinosaurids having higher von Mises stresses than abelisaurids and large carcharodontosaurids (Hypothesis 2). The quantitative results support the hypothesis that as tyrannosauroid cranial length (a proxy for body size) increased, the level of cranial stress (von Mises stress) decreased with normalized forces (Hypothesis 4). Notably, the von Mises averages in the crania of non‐tyrannosauroid theropods such as spinosaurids and allosauroids show a similar trajectory to that of tyrannosauroids (large carcharodontosaurids and spinosaurids exhibiting lower von Mises averages than medium‐sized allosauroids and spinosaurids), in contrast with a finding of Rayfield (2011).

While the crests of theropods (proceratosaurids and *Dilophosaurus*) will require further analysis (e.g., including internal crest structures), the von Mises analyses do not reject the role of the crests dissipating high stress in the crania of theropods such as *Guanlong* and *Dilophosaurus* (Hypothesis 3). For example, upon excluding the crests of theropods, the cranial stress magnitudes either remained unchanged (*Guanlong*) or the level of cranial stress increased (high nasal stress in *Dilophosaurus*). This may suggest that crests and their internal anatomy (pneumaticity shaping the bone) may have allowed crested theropods to resist high loadings (Hypothesis 3). Time‐calibrated phylogenies (Figure 17) indicate that as tyrannosauroids and other theropod lineages evolved, the average von Mises stress decreased, except for the ancestral lineages of Spinosauridae and Dromaeosauridae (Hypothesis 4). Small‐bodied tyrannosauroids (e.g., *Dilong*) show high cranial stress and low jaw muscle forces, and large‐bodied tyrannosauroids show relatively low cranial stress and high jaw muscle force (Johnson‐Ransom et al., 2024). Few medium‐sized tyrannosauroids of the mid‐Cretaceous have been recovered and may be important in evaluating the evolution of feeding performance in Tyrannosauroidea.

### 6.3 Allometric relationship between head size and cranial stress

The variable von Mises stress averages contradict the structurally expected correlation between head size and relative levels of cranial stress, where cranial stress decreases as cranial length increases. Some small theropods have relatively more robust crania than others in their size class. *Abelisaurus* has anteroposteriorly short crania for their size, which are relatively more robust than in large allosauroids and tyrannosauroids with skulls three times as long.

This lack of a trend for allometric cranial robustness with increasing size across Theropoda highlights the variety of adaptations between and within theropod clades. The cranium has functional and adaptive roles beyond feeding, such as harboring the brain and major somatic sense organs (Rowe & Rayfield, 2025). Theropod mandibles may be more subject to increasing robustness with element length through ontogeny and within lineages. This allometry occurred in tyrannosaurids (Ma et al., 2022; Rowe & Snively, 2021; Therrien et al., 2005; Therrien et al., 2021) whose mandibular ontogeny resembled that of durophagous Nile monitors Rieppel and Labhardt (1979), but not in *Coelophysis* (Jasinski, 2011). Similar within‐lineage comparisons have been enlightening about such trends and variation for crania as well, albeit at modest sample sizes (Johnson‐Ransom et al., 2024; Rayfield, 2011; Rowe & Rayfield, 2024).

### 6.4 Niche occupation by tyrannosauroids and other theropods: Synthesizing cranial stress with morphological, ecological, and dietary evidence

We broadly and testably infer the paleoecology and niche occupation and/or divergence in the theropod clades assessed in this study through comparisons of von Mises and principal stresses, and through analogy with the feeding behavior of modern predators (Bourke et al., 2008; Gignac & Erickson, 2016; McCurry et al., 2017; McHenry et al., 2006, 2007; Tseng, 2013; Tseng & Flynn, 2015; Walmsley et al., 2013; Wroe et al., 2005; Wroe et al., 2007).

#### 6.4.1 Inferred feeding behavior and paleoecology of non‐averostran theropods

*Coelophysis*, a phenotypic representative of early theropod lineages, was a relatively small carnivore, sharing its environment with relatively larger phytosaurs and pseudosuchians, including a non‐crocodyliform crocodylomorph (Irmis et al., 2007; Nesbitt & Sues, 2021). Because *Coelophysis* shows relatively low cranial stress magnitudes and a low von Mises stress value (1.4 MPa), it is possible that the early theropod may have fed upon similarly sized animals such as the shuvosaurid *Effigia* (Nesbitt & Norell, 2006) and the theropod dinosaur *Daemonosaurus* (Nesbitt & Sues, 2021), in addition to smaller prey (e.g., small co‐occurring reptiles and fishes; Rinehart et al., 2009; Nesbitt & Sues, 2021). The relatively long forelimbs and hooked hand claws of *Coelophysis* suggest the early theropod had good forelimb flexibility and range of motion, allowing it to grasp smaller prey (Carpenter, 2002). This is supported by an analysis of a small crocodylomorph found in the abdominal cavity of *Coelophysis* (Nesbitt & Norell, 2006).

Initial functional interpretations suggested that *Dilophosaurus* did not have a forceful bite, with the articulation of the premaxilla and maxilla likely being kinetic (Welles, 1984). Adults of the species are hypothesized to have fed on smaller prey such as fish, but may have hunted larger prey animals (Bakker, 1986; Milner & Kirkland, 2007; Paul, 1988; Therrien et al., 2005; Welles, 1984). In a redescription of *Dilophosaurus*, the articulation of the premaxilla and maxilla was found to be immobile and more robust than previously thought (Marsh & Rowe, 2018, 2020). These authors also suggested that *Dilophosaurus* had jaws that could puncture bone (Brown & Marsh, 2021; Marsh & Rowe, 2020), that the vertebrae of *Dilophosaurus* indicate avian‐style respiratory air sacs, and that the theropod was likely a fast and agile hunter (Brown & Marsh, 2021). The sauropodomorph *Sarahsaurus* was found with tooth marks along with shed teeth, possibly of *Dilophosaurus* (Marsh & Rowe, 2018, 2020). The robust skull morphology and low von Mises stress magnitudes (average von Mises stress value = 0.9 MPa) in *Dilophosaurus* support the non‐averostran theropod being a forceful biter. This is further supported by the lower von Mises and principal stresses of *Dilophosaurus*. The forelimbs were found to have been powerfully strong and flexible, with *Dilophosaurus* able to grip and hold objects between two hands and one hand, bring an object close to its mouth and belly, swing its arms in an arc, as well as scratch its belly (Senter & Sullivan, 2019).

#### 6.4.2 Inferred feeding behavior and paleoecology of Ceratosauria

*Ceratosaurus* and *Masiakasaurus* display high von Mises cranial stress averages, 1.9 and 1.8 MPa, respectively, in contrast to abelisaurids. The von Mises results imply that *Ceratosaurus* had a weak bite‐force performance and likely used its large ziphodont teeth to quickly slice and rake flesh (Rowe & Rayfield, 2025; Snively & Russell, 2007), coupled with robust dorsolatero‐ and ventroflexor craniocervical insertions (Snively & Russell, 2007). Because it coexisted with larger theropods, *Ceratosaurus* probably avoided the competition by hunting smaller, possibly more aquatic prey than other theropods of the Morrison Formation (Bakker & Bir, 2004; Foster & Chure, 2006; Henderson, 1998; Yun, 2019). *Ceratosaurus*' diet may not have been limited to feeding upon smaller animals, given the high occurrence of bite marks on the bones of *Allosaurus* likely from *Ceratosaurus* or *Torvosaurus*, indicative of intraspecific combat or scavenging (Drumheller et al., 2020). Similarly, Chure et al. (2000) document tooth marks on an *Allosaurus* pubis that may have been made by *Ceratosaurus* or *Torvosaurus* (a megalosauroid, not a ceratosaur). The noasaurid *Masiakasaurus* likely used its derived front teeth for grasping smaller prey, as well as cutting and slicing, and was likely incapable of tearing larger food apart (Carrano et al., 2002).

Abelisaurids consistently show relatively low von Mises values (0.7–1.6 MPa), attributed to their deeply robust crania, indicating that they would have resisted high forces and likely fed upon relatively large prey (e.g., sauropod dinosaurs). The von Mises results are comparable with previous biomechanical analyses of abelisaurids such as a study that showed the cranium of *Carnotaurus* to have been capable of withstanding high forces when biting and exerting a bite force of around 3341 N (Mazzetta et al., 2009). *Majungasaurus* was the only large predator of its environment and likely fed upon the titanosaurian sauropod *Rapetosaurus* (Rogers et al., 2009). Further evidence of this consists of shed abelisaurid teeth found with sauropod skeletons (Meso et al., 2021). *Majungasaurus* possessed craniocervical features that allowed for forceful feeding. These included robust cervical vertebrae serving as attachment sites for powerful neck muscles and a skull with ossified dermis creating a rough texture and conjoined nasals, which were pneumatized and thus dorsoventrally expanded (Sampson & Witmer, 2007) likely resulting in high structural strength (Snively et al., 2006, 2011; Therrien et al., 2005, 2021). Having deep, short skulls and ossified dermis likely facilitated forceful bites in abelisaurids (Hieronymus, 2009; Hieronymus et al., 2009; Sampson & Witmer, 2007).

#### 6.4.3 Inferred feeding behavior and paleoecology of megalosauroids

A biomechanical study by Rowe and Rayfield (2025) evaluated the feeding performance of the megalosaurid *Torvosaurus* in comparison with other theropods. *Torvosaurus* had relatively low von Mises cranial stress than the spinosaurids *Suchomimus* and *Spinosaurus*, but relatively high mandibular stress (Rowe & Rayfield, 2025). The skulls of *Torvosaurus* and other megalosaurids are incomplete, with the missing elements being filled in and modified by close relatives (e.g., spinosaurids, Rowe & Rayfield, 2025). The North American megalosaurid *Torvosaurus* was a large, robust predator with an estimated body length of 10 m and a body mass of 3.6–4.5 tons (Galton & Jensen, 1979; Hendrickx & Mateus, 2014; Paul, 1988). Compared with *Allosaurus Torvosaurus* may have preferred hunting in a wetland environment, similar to *Ceratosaurus*, based on sedimentological evidence, although it could be attributed to preservation bias (Bakker & Bir, 2004). Bakker and Bir (2004) also state that many *Allosaurus* teeth were found in aquatic environments, as possible evidence for a seasonal shift in diet away from large terrestrial dinosaurs during the dry season. Rauhut et al. (2016) found that megalosauroids may have preferred nearshore over inland environments, but caution that geographic biases may also explain these results, and that these environmental categories may encompass multiple habitats. The low von Mises stresses in the 3D (Rowe & Rayfield, 2025) and 2D (1.6 MPa) cranial models of *Torvosaurus* suggest that the large megalosaurid hunted large terrestrial prey such as the *Stegosaurus* and sauropods and may have been capable of resisting high forces. As mentioned previously, it is likely that *Torvosaurus* may have engaged in interspecific combat with other theropods (Drumheller et al., 2020) and is at least one candidate for leaving feeding traces on an *Allosaurus* pubis (Chure et al., 2000).

This current study and the 3D analysis by Rowe and Rayfield (2025) show spinosaurids having relatively low cranial and mandibular stress except for *Baryonyx*. The relatively low von Mises stress values of *Spinosaurus* and *Suchomimus*, and the variable stress magnitudes indicate spinosaurids were not limited to piscivory, although they lacked relatively forceful biting compared to other theropods (D'Amore et al., 2024). While the diet and lifestyle of spinosaurids have initially been attributed to piscivory, spinosaurids had a more diverse diet (Buffetaut et al., 2004; Charig & Milner, 1997; Cuff & Rayfield, 2013; D'Amore et al., 2024; Hone & Holtz, 2021; Ibrahim et al., 2014; Therrien et al., 2005). Juvenile *Iguanodon* elements were found in the stomach cavity of a *Baryonyx*, and a spinosaurid tooth was embedded in the cervical vertebrae of a pterosaur (Buffetaut et al., 2004; Charig & Milner, 1997). The larger *Spinosaurus* (averages von Mises cranial stress value = 1.5 MPa) displayed low stress magnitudes at the nasals, lacrimal, nasal‐lacrimal suture, and temporal complex. The rostral morphology of *Spinosaurus* suggests that it resisted bending in the vertical direction but had jaws that were poorly adapted to resisting lateral bending compared to other spinosaurids (e.g., *Baryonyx*) (Cuff & Rayfield, 2013; Hone & Holtz, 2021; Ibrahim et al., 2014). Spinosaurids likely had pronounced neck muscles enhancing their ability to strike quickly and withdraw their head rapidly (Evers et al., 2015; Sues et al., 2002). Spinosaurids likely frequented semiaquatic environments based on the isotopic composition of the teeth, neuroanatomy, bone histology, and skull morphology, lowering direct competition with terrestrial theropods (Amiot et al., 2010; D'Amore et al., 2024; Hassler et al., 2018; Hone & Holtz, 2021; Ibrahim et al., 2014; Schade et al., 2020; Sereno et al., 2023). Estimated von Mises results are consonant with spinosaurids feeding upon large aquatic prey, in addition to non‐aquatic animals (e.g., small dinosaurs and pterosaurs).

#### 6.4.4 Inferred feeding behavior and paleoecology of allosauroids

The clade Allosauroidea shows an average von Mises cranial stress values of 1.1 MPa, indicative that allosauroids (specifically the large carcharodontosaurids) could resist high forces and feeding on relatively large prey (e.g., sauropods). Although allosauroids were apex predators, the bite forces of allosauroids were lower in comparison to tyrannosaurids, and they showed high cranial stress at equivalent force (Rayfield et al., 2001; Rowe & Rayfield, 2025; Therrien et al., 2005, 2021). *Allosaurus'* cranium has average von Mises stress of 1.6 MPa, indicative of the allosaurid resisting high forces. Because of the relatively low von Mises stress and gracile craniocervical morphology (Lautenschlager, 2015; Rayfield et al., 2001; Snively et al., 2013), *Allosaurus* likely fed upon large sauropods and ornithischians (e.g., *Stegosaurus* and *Camptosaurus*; Dodson et al., 1980; Carpenter et al., 2005; Drumheller et al., 2020; Lei et al., 2023). Other large theropods (*Ceratosaurus* and *Torvosaurus*) coexisted with *Allosaurus*, but likely had different predatory niches based on their differing anatomical adaptations and fossil locations (Henderson, 1998; Mateus et al., 2006; Snively & Russell, 2007). Anatomical adaptations of the two theropods include *Allosaurus* having powerful ventroflexor dynamics of the head and neck, ziphodont dentition, modestly powerful adductor jaw muscles, whereas *Ceratosaurus* may have had greater dorsolateroflexive torque, and a more brevirostrine cranium (Rowe & Snively, 2021; Snively et al., 2013; Snively & Russell, 2007). *Allosaurus* was likely fed upon by larger theropods (larger *Allosaurus*, *Torvosaurus*, or *Ceratosaurus*) and *Ceratosaurus* either as a result of inter‐or intra‐specific combat, predation, or scavenging based on a high frequency of bitemarks on *Allosaurus* bones (Chure et al., 2000; Drumheller et al., 2020). Based on *Allosaurus* bite marks on sauropod bones and the low average von Mises cranial stress estimations in carcharodontosaurids, it is likely that the large carcharodontosaurids fed upon the large titanosaurian sauropods with the two dinosaur groups coexisting in similar environments (Leanza et al., 2004; Motta et al., 2016; Novas et al., 2013). The crania and mandibles of carcharodontosaurids such as *Carcharodontosaurus*, *Giganotosaurus* and *Acrocanthosaurus* lacked the adaptations to resist torsional loads seen in tyrannosaurids (Snively et al., 2006; Therrien et al., 2005, 2021), and had relatively low jaw muscle forces (Sakamoto, 2022). The ziphodont teeth of carcharodontosaurids had less bending strength (Snively et al., 2006) and, like those of juvenile tyrannosaurids, were less capable of resisting torsional stresses from struggling prey and bone contact than the incrassate teeth of adult tyrannosaurids (Therrien et al., 2021). The relatively high stress magnitudes in allosauroids, including carcharodontosaurids (average von Mises stress value = 1.3 MPa), are consistent with a different predation style compared with that of tyrannosaurids (average von Mises stress value = 1.2 MPa, including higher‐stressed juveniles and alioramins; Snively et al., 2019).

#### 6.4.5 Inferred feeding behavior and paleoecology of clades proximate to Tyrannosauroidea

The ecology and feeding behavior of megaraptorans are relatively under‐studied, but their anatomy suggests that megaraptorans would have functioned as active predators (White et al., 2015). Megaraptorans are characterized by their slender build, gracile‐snouted skulls, and long forelimbs with strongly curved hand claws (Coria & Currie, 2016; Porfiri et al., 2014; Porfiri et al., 2018; White et al., 2015). The composite megaraptorid shows an average von Mises stress value of 1.3 MPa, indicative of relatively low cranial stress and may have fed upon small prey (small ornithopods) and relatively large animals (e.g., titanosaurs and large ornithopods). Megaraptorans shared their environment with abelisaurids, small ornithopods, and titanosaurs (White et al., 2015). The skull of megaraptorans was relatively gracile and contained ziphodont teeth, indicative that megaraptorans lacked the crushing bite force in deep‐snouted tyrannosaurids. The forelimbs of megaraptorans were capable of wide ranges of motion similar to maniraptorans and allosauroids, which together suggests that megaraptorans could have used their long‐clawed forelimbs to grab smaller animals and dispatched prey with their jaws (White et al., 2015). A recently described megaraptorid, *Joaquinraptor*, was discovered with a crocodyliform humerus in contact with the dentary tooth crowns of the holotype specimen. The crocodyliform humerus may bear tooth marks, and further taphonomic studies are underway to determine how these two animals likely came to be associated with each other (Ibiricu et al., 2025).

von Mises stresses in sampled compsognathids are lower than in other coelurosaurian theropod outgroups to Tyrannosauroidea. “Compsognathidae” as usually constituted was recently found to be polyphyletic, with specimens of some taxa (mostly excluded here) being juveniles and/or small representatives of other tetanuran theropod clades (Cau, 2024). Small compsognathids (traditionally classified; Cau, 2024) such as *Compsognathus* show relatively low von Mises stress averages (1.5 MPa) indicative of the small compsognathid showing low cranial stress during feeding. A small lizard was found within the stomach cavity of one *Compsognathus* specimen (Conrad, 2018; Nopcsa, 1903; Ostrom, 1978) with small lizard or sphenodontid remains in another specimen (Peyer, 2006), suggesting that smaller animals were part of *Compsognathus'* diet. Larger compsognathids such as *Sinocalliopteryx* and *Huaxiagnathus* show relatively low von Mises stress values and low cranial stress magnitudes, indicating that they may have fed upon prey similar in size to themselves in addition to smaller animals. The well‐preserved specimens of *Sinocalliopteryx* were found preserved with the bones of smaller vertebrates such as the pes and distal part of a leg from a dromaeosaurid, the avian *Confuciusornis*, and the scapula of an herbivorous ornithischian (Xing, Bell, Persons, et al., 2012). Large compsognathids such as *Sinocalliopteryx* were likely cursorial and capable of hunting agile prey, including birds (Xing, Bell, Persons, et al., 2012).

Dromaeosaurids fed upon a variety of prey. Remains of *Deinonychus* have been found in association with the ornithopod *Tenontosaurus* (Brinkman et al., 1998; Forster, 1984; Gignac et al., 2010; Maxwell & Ostrom, 1995). Puncture marks were found on the bones of a *Tenontosaurus* attributed to *Deinonychus*, with physical tests revealing that *Deinonychus* could exert a high bite force between 4100 to 8200 N (Gignac et al., 2010), coupled with its low von Mises cranial stress average (1.7 MPa). Despite having a high bite force, *Deinonychus* may not have consumed bone as regularly as *Tyrannosaurus*, with its wedge‐like teeth (Gignac et al., 2010). Velociraptorines such as *Tsaagan* show high cranial stress magnitudes and an average von Mises value of 1.7 MPa, implying that the dromaeosaurids including *Deinonychus* used the sickle‐claw on their toes to capture and restrain prey, while using their jaws as the killing weapon (Bishop, 2019; Fowler, Freedman, et al., 2011). These dromaeosaurids did feed upon larger animals, with a *Velociraptor* consuming a *Protoceratops* carcass and another with the long bone of an azhdarchid pterosaur preserved within its ribcage (Hone et al., 2010; Hone et al., 2012). In contrast to the crania of robust dromaeosaurids, the small, gracile unenlagiine dromaeosaurid *Buitreraptor* shows especially high cranial stress magnitudes and a von Mises stress value of 3.1 MPa. *Buitreraptor* likely would have hunted for smaller prey (Gianechini et al., 2020). Because *Buitreraptor* lacked the serrated teeth capable of tearing flesh, its teeth likely enabled it to hold and swallow its prey whole (Gianechini et al., 2020). Troodontids were unique among the coelurosaurian outgroups to tyrannosauroids. Troodontids may have been omnivorous, given their dental morphology and microwear (Cullen & Cousens, 2024; Holtz et al., 1998). Biomechanical analyses indicated that the dental microwear of troodontids was not suited for feeding on similarly sized prey in contrast to the dental microwear of dromaeosaurids (Cullen & Cousens, 2024; Torices et al., 2018). This would suggest that troodontids would have hunted smaller animals and scavenged, in addition to being omnivorous (Cullen & Cousens, 2024; Torices et al., 2018). Qualitatively, the cranium of *Zanabazar* showed low von Mises stress, but the sampled cranial points and von Mises average were relatively greater than the studied dromaeosaurids (e.g., *Tsaagan* and *Deinonychus*). *Zanabazar* had an average von Mises stress of 1.9 MPa, higher than that of *Tsaagan* (1.7 MPa) and *Deinonychus* (1.7 MPa).

#### 6.4.6 Inferred feeding behavior and paleoecology of tyrannosauroids

Consistently, all the tyrannosauroid crania showed stress occurring at the nasals and nasal‐lacrimal connection, supporting the hypothesis that the nasals' fusion, transverse arching, and interlocking articulation with other bones resisted high feeding forces (Rauhut et al., 2009; Snively et al., 2006).

Smaller tyrannosauroids such as *Dilong* and the early‐stage juvenile tyrannosaurids (*Raptorex*) had delicate crania and were less capable of resisting high bite forces (Rauhut et al., 2009; Rowe & Snively, 2021; Xu et al., 2004) than those of adults. Other small tyrannosauroids may have fed on smaller animals and possibly prey of similar size to themselves based on low cranial stress in *Guanlong* and the average von Mises stress (0.3 MPa in the crested version and 2.7 MPa in the crestless version; Rauhut et al., 2009; Xu et al., 2006). However, low stress in the crests reduced evident overall stress in the crania, but the crests themselves were outside the trajectory of force loading and thus may not have acted as stress sinks. Small, early tyrannosauroids (e.g., *Guanlong*) would have functioned as general carnivores, especially as contemporaries to larger predatory allosauroids (e.g., *Sinraptor*).

Medium‐sized tyrannosauroids such as *Teratophoneus*, *Qianzhousaurus*, and late‐stage juvenile tyrannosaurids showed lower cranial stress than smaller tyrannosauroid taxa, with few exceptions. The subadult eutyrannosaurian (BMRP 2002.4.1) and the alioramin *Qianzhousaurus* showed higher cranial stress than deep‐snouted tyrannosauroids. The crania of the aforementioned tyrannosauroids were rather gracile, in conjunction with their slender body frame, implying that juvenile *Tyrannosaurus*, *Nanotyrannus* and alioramins fed on more agile, smaller‐bodied prey (e.g., ornithomimosaurs) or scavenged (Currie, 1983; Currie & Eberth, 2010; Dececchi et al., 2020; Erickson et al., 2004; Foster et al., 2022; Holtz, 2021; Hutchinson et al., 2011; Lü et al., 2014; McCrea et al., 2014; Persons & Currie, 2014; Schroeder et al., 2021; Snively et al., 2019; Zanno & Napoli, 2025). Bite marks on the bones of hadrosaurids by a subadult eutyrannosaurian also suggest that *Nanotyrannus* and juvenile *Tyrannosaurus* may have fed on larger herbivores as well (Peterson et al., 2021; Peterson & Daus, 2019). *Qianzhousaurus* showed higher cranial stress than the subadult eutyrannosaurian, suggesting that the crania of alioramins were not adapted to resist high forces (Rowe & Rayfield, 2024). The slender‐snouted alioramins are a unique case where their relatively gracile jaws may have been incapable of delivering forceful bites, and they may have hunted smaller and more gracile prey, possibly similar to the ecology of *Nanotyrannus* and a late‐stage juvenile *Tyrannosaurus* (Foster et al., 2022; Lü et al., 2014). The robust cranium and high jaw muscle forces of *Teratophoneus* may have allowed it to feed on large ornithischians that shared its environment, such as hadrosaurids (e.g., *Parasaurolophus* and *Gryposaurus*) and ceratopsians (e.g., *Nasutoceratops* and *Utahceratops*) (Holtz, 2021; Johnson‐Ransom et al., 2024; Rowe & Rayfield, 2024, 2025; Schroeder et al., 2021; Titus et al., 2021; Zanno & Sampson, 2005). Despite being similar in body size, deep‐snouted tyrannosaurids such as *Teratophoneus* showed lower cranial stress than a subadult eutyrannosaurian (BMRP 2002.4.1) and *Qianzhousaurus*.

Larger tyrannosauroids, especially the deep‐snouted taxa, showed lower cranial stress, indicating that their crania were capable of resisting high forces and the animals were capable of feeding on a variety of large prey (e.g., ceratopsians, hadrosaurids, ornithomimosaurs, and possibly sauropods; Rowe & Snively, 2021; Therrien et al., 2021; Holtz, 2021; Schroeder et al., 2021; Rowe & Rayfield, 2024, 2025). The large proceratosaurid *Yutyrannus* also showed low cranial stress similar to the crania of the deep‐snouted tyrannosaurids. *Yutyrannus* may have fed upon the sauropod *Dongbetitan*, they co‐occur in the Yixian Formation (Xing, Bell, Currie, et al., 2012; Xu et al., 2012), and a large theropod tooth, possibly from *Yutyrannus*, was discovered embedded in the rib of *Dongbetitan*, possibly the result of scavenging (Xing, Bell, Currie, et al., 2012). *Bistahieversor* was one of the earliest deep‐snouted tyrannosauroids to achieve a large body size and likely fed on larger ornithischians such as the ceratopsids *Terminocavus* and *Navajoceratops* and the hadrosaurid *Kritosaurus* (Fowler, 2017; Fowler & Freedman Fowler, 2020). In addition to a deeply robust skull, *Bistahieversor* possessed heightened senses of smell and good gaze stabilization (associated with agility), based on the morphology of its endocranium (McKeown et al., 2020). Features such as these would be consistent in later‐diverging tyrannosauroids (e.g., Tyrannosauridae). Similarly sized tyrannosaurids such as the tyrannosaurine *Daspletosaurus* and the albertosaurine *Gorgosaurus* shared similar environments but are hypothesized to have occupied their own predatory niches, with *Daspletosaurus* feeding on the more robust ceratopsians and *Gorgosaurus* feeding on the more gracile hadrosaurids (Farlow & Pianka, 2002; Russell, 1970). Larger tyrannosaurids such as *Tyrannosaurus* and *Tarbosaurus* were apex carnivores, with bite marks on the bones of various herbivorous dinosaurs (e.g., *Triceratops*, *Edmontosaurus*, *Saurolophus*, and *Deinocheirus*) attributed to them (Carpenter, 1997; Chin et al., 1998; DePalma et al., 2013; Happ, 2008; Hone & Watabe, 2010; Hurum & Sabath, 2003; Rothschild & DePalma, 2013; Varricchio, 2001).

## 7 CONCLUSION AND FUTURE DIRECTIONS

The presented results show the comparative feeding performance throughout the evolutionary history of tyrannosauroids and non‐tyrannosauroid theropods. Small early tyrannosauroids had gracile skulls and relatively lower jaw muscle forces than contemporaneous theropods with relatively deeper skulls. Upon the extinction of the large allosauroids in the Northern Hemisphere, the small early tyrannosauroids attained larger body sizes with relatively robust skulls and powerful jaw muscles capable of exerting incredible bite forces. Large non‐tyrannosauroid theropods (*Spinosaurus* and *Giganotosaurus*) and short‐faced abelisaurids show relatively low cranial stress magnitudes, suggesting that their skulls evolved to manage the high‐force regimes they routinely experienced.

Biomechanical and phylogenetic comparative analyses are important to assess the evolution of feeding performance in extinct clades. Future analyses will include using linear measurements and statistical analyses, including analysis of covariance (ANCOVA) to evaluate potential correlations between the skull shape (brevirostrine vs. longirostrine) and mechanical advantage of theropod feeding.

## AUTHOR CONTRIBUTIONS

**Evan Johnson‐Ransom:** Conceptualization; investigation; funding acquisition; writing – original draft; methodology; validation; visualization; writing – review and editing. **Paul Gignac:** Methodology; validation; writing – review and editing. **Daniel E. Barta:** Methodology; writing – review and editing; formal analysis; software; visualization; validation. **Ryan N. Felice:** Writing – review and editing; methodology; visualization; software; formal analysis. **Eric Snively:** Conceptualization; writing – review and editing; supervision; validation; writing – original draft; methodology; visualization.

## Supporting information


**Data S1.** Supporting Information.

**Data S2.** Supporting Information.

**Figure S1.** First principal stress (megapascals) in 2D planar models of early theropods, *Coelophysis bauri* (b) and *Dilophosaurus wetherilli* (a); “*” indicates theropod model being a composite. First principal stress primarily reflects tension. Height represents magnitude of tension and depth represents magnitude of compression. The peak magnitudes reflect artifacts at constraints (e.g., anterior teeth and jaw joint). Both *Coelophysis bauri* (b) and *Dilophosaurus wetherilli* (a) show tensile stress occurring at the maxillary‐jugal suture, lacrimal bar, jugal (representative of the pterygoids), and temporal complex. The peak tensile stresses that occur in the cranium of *Dilophosaurus wetherilli* (a) are lower than the tensile stresses in *Coelophysis bauri* (b). *Coelophysis bauri* (b) shows a higher distribution of tensile stress at the jugal and postorbital.

**Figure S2.** Third principal stress (megapascals) in orthogonal models of early theropods, *Coelophysis bauri* (b) and *Dilophosaurus wetherilli* (a); “*” indicates theropod model being a composite. Third principal stress reflects compression. Depth represents magnitude of compression. The peak magnitudes reflect artifacts at constraints (e.g., anterior teeth and jaw joint). Both *Dilophosaurus wetherilli* (a) and *Coelophysis bauri* (b) show high compressive stress in areas of the crania such as the nasals, temporal complex, lacrimals, and jugals.

**Figure S3.** First principal stress (megapascals) in 2D planar models of *Ceratosaurus nasicornis* (f), *Masiakasaurus knopfleri* (e), and abelisaurids [*Carnotaurus sastrei* (a), *Majungasaurus crenatissimus* (b), *Skorpiovenator bustingorryi* (c), and *Abelisaurus comahuensis* (d)]; “*” indicates theropod model being a composite. First principal stress primarily reflects tension. Height represents magnitude of tension, and depth represents magnitude of compression. The peak magnitudes reflect artifacts at constraints (e.g., anterior teeth and jaw joint). While the crania of *Ceratosaurus nasicornis* (f) and *Masiakasaurus knopfleri* (e) show overall low stress, moderately high tensile stress occurs at the temporal complex, postorbital, lacrimal bar, and jugal. The tensile stress in the cranium of *Masiakasaurus knopfleri* (e) is higher than the cranium of *Ceratosaurus nasicornis* (f). The abelisaurids show overall low stress, although the cranium of *Carnotaurus* shows higher stress. Similar to *Ceratosaurus nasicornis* (f) and *Masiakasaurus knopfleri* (e), the abelisaurids [*Carnotaurus sastrei* (a), *Majungasaurus crenatissimus* (b), *Skorpiovenator bustingorryi* (c), and *Abelisaurus comahuensis* (d)] exhibit peak tensile stresses at similar areas of the crania such as the jugal, lacrimal, and maxillary‐fugal suture. The peak tensile stresses in *Carnotaurus sastrei* (a) and *Abelisaurus comahuensis* (d) are higher than *Skorpiovenator bustingorryi* (c) and are widely distributed.

**Figure S4.** Third principal stress (megapascals) in 2D planar models of *Ceratosaurus nasicornis* (f), *Masiakasaurus knopfleri* (e), and abelisaurids [*Carnotaurus sastrei* (a), *Majungasaurus crenatissimus* (b), *Skorpiovenator bustingorryi* (c), and *Abelisaurus comahuensis* (d)]; “*” indicates theropod model being a composite. Third principal stress reflects compression. Depth represents magnitude of compression. The peak magnitudes reflect artifacts at constraints (e.g., anterior teeth and jaw joint). The abelisaurids display low compressive stress, with the exception of *Masiakasaurus knopfleri* (e) and *Ceratosaurus nasicornis* (f) showing higher compressive stress, at the temporal complexes, nasals, lacrimals, and jugals. Abelisaurids [*Carnotaurus sastrei* (a), *Majungasaurus crenatissimus* (b), *Skorpiovenator bustingorryi* (c), and *Abelisaurus comahuensis* (d)] show higher peak compressive stress at the back of the crania.

**Figure S5.** First principal stress (megapascals) in 2D planar models of megalosauroids and basal tetanuran [e.g. *Monolophosaurus jiangi* (g)]; “*” – indicates theropod model being a composite. First principal stress primarily reflects tension. Height represents magnitude of tension, and depth represents magnitude of compression. The peak magnitudes reflect artifacts at constraints (e.g. anterior teeth and jaw joint). The crania of *Monolophosaurus jiangi* (g) and the megalosaurids [*Torvosaurus tanneri* (d), *Afrovenator abakensis* (e), and *Dubreuillosaurus valesduensis* (f)] show moderately high tensile peak stresses that consistently occur at the maxillary‐fugal suture, lacrimal, jugal, temporal complex, and postorbital. The crania of spinosaurids [*Spinosaurus aegyptiacus* (a), *Suchomimus tenerensis* (b), and *Baryonyx walkeri* (c)] show tensile stress occurring at the jugal, squamosal, and the lacrimal and postorbital bar. *Monolophosaurus jiangi*, megalosaurids, and spinosaurids show an increase in the level of tensile stress as theropod body size increases, with the larger *Torvosaurus tanneri* (d) and *Spinosaurus* showing higher tensile stress than the smaller *Dubreuillosaurus valesdunensis* (f) and *Baryonyx walkeri* (c), respectively.

**Figure S6.** Third principal stress (megapascals) in 2D planar models of megalosauroid and basal tetanuran [e.g. *Monolophosaurus jiangi* (g)]theropods; “*” – indicates theropod model being a composite. Third principal stress reflects compression. Depth represents magnitude of compression. The peak magnitudes reflect artifacts at constraints (e.g., anterior teeth and jaw joint). Megalosaurids [*Torvosaurus tanneri* (d), *Afrovenator abakensis* (e), and *Dubreuillosaurus valesdunensis* (f)] show high compressive peak stresses that are widely distributed throughout the crania, appearing at the nasals, nasal‐lacrimal suture, temporal complex, and jugal. The level of peak compressive stress decreases in the size of megalosaurids. Spinosaurids [*Spinosaurus aegyptiacus* (a), *Suchomimus tenerensis* (b), and *Baryonyx walkeri* (c)] show high compressive stress at the nasals and temporal complex, but the level of compressive stress increases as spinosaurids' body size increases. *Monolophosaurus jiangi* (g) shows moderately high compressive peak stress that is present at the cranium, such as the crest, nasals, temporal complex, quadratojugal, and temporal complex.

**Figure S7.** First principal stress (megapascals) in 2D planar models of allosauroids; “*” indicates theropod model being a composite. First principal stress primarily reflects tension. Height represents magnitude of tension and depth represents magnitude of compression. The peak magnitudes reflect artifacts at constraints (e.g., anterior teeth and jaw joint). The crania of allosauroids show high tensile stress occurring at maxillary‐jugal suture, nasals, nasal‐lacrimal suture, lacrimal, jugal, temporal complex, and postorbital. Both *Sinraptor dongi* (g) and the large carcharodontosaurids [*Giganotosaurus carollini* (a), *Carcharodontosaurus iguidensis* (b), *Mapusaurus roseae* (c), and *Acrocanthosaurus atokensis* (d)] show a wide distribution of tensile stress at the aforementioned areas of the crania, with high magnitudes being present at the crania, whereas *Allosaurus jimmadseni* (e), *Yangchuanosaurus magnus* (h), and *Asfaltovenator vialidadi* (f) show a low magnitude of tensile stress.

**Figure S8.** Third principal stress (megapascals) in 2D planar models of allosauroid theropods; “*” indicates theropod model being a composite. Third principal stress reflects compression. Depth represents magnitude of compression. The peak magnitudes reflect artifacts at constraints (e.g., anterior teeth and jaw joint). Allosauroids show low compressive stress at the nasals, temporal complex, postorbital, and jugal. Similar to megalosaurids, the level of peak compressive stress decreases as allosauroid body size increases. The large carcharodontosaurids [*Giganotosaurus carollini* (a), *Carcharodontosaurus iguidensis* (b), *Mapusaurus roseae* (c), and *Acrocanthosaurus atokensis* (d)] show low peak compressive stress in areas of the crania than smaller allosauroids such as *Allosaurus jimmadseni* (e), *Asfaltovenator vialidadi* (f), and the metriacanthosaurids [*Sinraptor dongi* (g) and *Yangchuanosaurus magnus* (h)].

**Figure S9.** First principal stress (megapascals) in 2D planar models of maniraptoran theropods; “*” indicates theropod model being a composite. First principal stress primarily reflects tension. Height represents magnitude of tension and depth represents magnitude of compression. The peak magnitudes reflect artifacts at constraints (e.g., anterior teeth and jaw joint). Dromaeosaurids [*Tsaagan mangas* (a), *Deinonychus antirrhopus* (b), and *Buitreraptor gonzalezorum* (c)] show tensile stress occurring at areas of the crania similar to compsognathids, with *Tsaagan mangas* (a) and *Deinonychus antirrhopus* (b) showing high peak tensile stress occurring at the jugals. Both the troodontid *Zanabazar junior* (d) and *Ornitholestes hermanni* (e) show low tensile stress occurring at similar areas of the cranium, except for *Ornitholestes hermanni* (e) showing high peak tensile stress occurring at the jugal.

**Figure S10.** Third principal stress (megapascals) in 2D planar models of maniraptoran theropods; “*” indicates theropod model being a composite. Third principal stress reflects compression. Depth represents magnitude of compression. The peak magnitudes reflect artifacts at constraints (e.g., anterior teeth and jaw joint). The crania of the maniraptorans [*Tsaagan mangas* (a), *Deinonychus antirrhopus* (b), and *Buitreraptor gonzalezorum* (c), *Zanabazar junior* (d), and *Ornitholestes hermanni* (e)] show high compressive stress at the posterior region of the crania (e.g., temporal complex, squamosal, jugal) and the nasals.

**Figure S11.** First principal stress (megapascals) in 2D planar models of coelurosaurian theropods with phylogenetic affinities to Tyrannosauroidea; “*” indicates theropod model being a composite. First principal stress primarily reflects tension. Height represents magnitude of tension, and depth represents magnitude of compression. The peak magnitudes reflect artifacts at constraints (e.g., anterior teeth and jaw joint). The crania of compsognathids show low tensile stress at the nasal‐lacrimal suture, temporal complex, squamosal, and lacrimal, with *Huaxiagnathus orientalis* (d) showing a wide distribution of tensile stress. Compsognathids show a high peak tensile stress occurring at the jugals. Similar to megalosauroids, the level of tensile stress increases ascompsognathid body size increases, with *Compsognathus longipes* (b) showing low tensile stress and *Sinocalliopteryx gigas* (c) showing high tensile stress. The cranium of the composite megaraptorid (a) shows low tensile stress at the maxillary‐jugal suture, nasal‐lacrimal suture, jugal, and temporal complex.

**Figure S12.** Third principal stress (megapascals) in 2D planar models of coelurosaurian theropods with phylogenetic affinities to Tyrannosauroidea; “*” indicates theropod model being a composite. Third principal stress reflects compression. Depth represents magnitude of compression. The peak magnitudes reflect artifacts at constraints (e.g., anterior teeth and jaw joint). The compsognathids [*Compsognathus longipes* (b), *Sinocalliopteryx gigas* (c), and *Huaxiagnathus orientalis* (d)] show high peak compressive stress at the back of the cranium (e.g., jugal, temporal complex, and squamosal bar). The composite megaraptorid (a) shows compressive stress occurring at the temporal complex, jugal, quadratojugal, nasals, and nasal‐lacrimal suture.

**Figure S13.** Cladogram depicting the average von Mises stress (Pascals) in the ancestral condition of theropod clades, specifically the nasals. The ancestral conditions of Abelisauridae, Allosauroidea, and Tyrannosauroidea show relatively low nasal stress than Megalosauroidea and Maniraptora. The theropod clades depicted include Ceratosauria (1), Megalosauroidea (2), Allosauroidea (3), Maniraptora (4), Compsognathidae (5), and Tyrannosauroidea (6). Artist credit: Scott Hartman (*Coelophysis*, *Ceratosaurus*, *Majungasaurus*, *Carnotaurus*, *Giganotosaurus*, *Deinonychus*, *Monolophosaurus*, *Guanlong*, *Bistahieversor*), Jagged Fang Designs (*Compsognathus*, *Torvosaurus*, *Acrocanthosaurus*, *Daspletosaurus*), Ivan Iofrida (*Spinosaurus*), Tasman Dixon (*Dilophosaurus*, *Allosaurus*, *Yutyrannus*), and Jack Mayer Wood (*Tyrannosaurus*), Alessio Ciaffi (*Abelisaurus*), Ivan Iofrida (*Baryonyx*, *Buitreraptor*, *Gorgosaurus*), Matt Martyniuk (*Ornitholestes*, *Tarbosaurus*), T. K. Robinson (composite megaraptorid), Ashley Patch (*Qianzhousaurus*), Conty (*Tarbosaurus*), and T. Michael Keesey (*Tarbosaurus*).

**Figure S14.** Cladogram depicting the average von Mises stress (Pascals) in the ancestral condition of theropod clades, specifically the lacrimal‐jugal suture. The sampled lacrimal‐jugal suture stress values were relatively greater than the sampled nasal stresses. The ancestral conditions of Abelisauridae and Allosauroidea show relatively lower lacrimal‐jugal stress than Tyrannosauroidea and Megalosauroidea. The ancestral condition of Maniraptora shows the highest lacrimal‐jugal stress than the previously mentioned theropod clades. The theropod clades depicted include Ceratosauria (1), Megalosauroidea (2), Allosauroidea (3), Maniraptora (4), Compsognathidae (5), and Tyrannosauroidea (6). Artist credit: Scott Hartman (*Coelophysis*, *Ceratosaurus*, *Majungasaurus*, *Carnotaurus*, *Giganotosaurus*, *Deinonychus*, *Monolophosaurus*, *Guanlong*, *Bistahieversor*), Jagged Fang Designs (*Compsognathus*, *Torvosaurus*, *Acrocanthosaurus*, *Daspletosaurus*), Ivan Iofrida (*Spinosaurus*), Tasman Dixon (*Dilophosaurus*, *Allosaurus*, *Yutyrannus*), and Jack Mayer Wood (*Tyrannosaurus*), Alessio Ciaffi (*Abelisaurus*), Ivan Iofrida (*Baryonyx*, *Buitreraptor*, *Gorgosaurus*), Matt Martyniuk (*Ornitholestes*, *Tarbosaurus*), T. K. Robinson (composite megaraptorid), Ashley Patch (*Qianzhousaurus*), Conty (*Tarbosaurus*), and T. Michael Keesey (*Tarbosaurus*).

**Figure S15.** Cladogram depicting the average von Mises stress (Pascals) in the ancestral condition of theropod clades, specifically the nasal‐lacrimal suture. Similar to the lacrimal‐jugal suture, the nasal‐lacrimal suture shows relatively greater stress averages than the nasals. The ancestral conditions of Abelisauridae, Allosauroidea, and Tyrannosauroidea show relatively low nasal‐lacrimal stress than Megalosauroidea and Maniraptora. The theropod clades depicted include Ceratosauria (1), Megalosauroidea (2), Allosauroidea (3), Maniraptora (4), Compsognathidae (5), and Tyrannosauroidea (6). Artist credit: Scott Hartman (*Coelophysis*, *Ceratosaurus*, *Majungasaurus*, *Carnotaurus*, *Giganotosaurus*, *Deinonychus*, *Monolophosaurus*, *Guanlong*, *Bistahieversor*), Jagged Fang Designs (*Compsognathus*, *Torvosaurus*, *Acrocanthosaurus*, *Daspletosaurus*), Ivan Iofrida (*Spinosaurus*), Tasman Dixon (*Dilophosaurus*, *Allosaurus*, *Yutyrannus*), and Jack Mayer Wood (*Tyrannosaurus*), Alessio Ciaffi (*Abelisaurus*), Ivan Iofrida (*Baryonyx*, *Buitreraptor*, *Gorgosaurus*), Matt Martyniuk (*Ornitholestes*, *Tarbosaurus*), T. K. Robinson (composite megaraptorid), Ashley Patch (*Qianzhousaurus*), Conty (*Tarbosaurus*), and T. Michael Keesey (*Tarbosaurus*).

**Figure S16.** Cladogram depicting the average von Mises stress (Pascals) in the ancestral condition of theropod clades, specifically the inferior jugal. The sampled inferior jugal stresses were relatively greater than the sampled nasal‐lacrimal, nasal, and lacrimal‐jugal stress averages. The ancestral conditions of Abelisauridae and Allosauroidea show relatively lower inferior jugal stress than Tyrannosauroidea and Megalosauroidea. The ancestral condition of Maniraptora shows the highest inferior jugal stress than the previously mentioned theropod clades. The theropod clades depicted include Ceratosauria (1), Megalosauroidea (2), Allosauroidea (3), Maniraptora (4), Compsognathidae (5), and Tyrannosauroidea (6). Images from Phylopic. Artist credit: Scott Hartman (*Coelophysis*, *Ceratosaurus*, *Majungasaurus*, *Carnotaurus*, *Giganotosaurus*, *Deinonychus*, *Monolophosaurus*, *Guanlong*, *Bistahieversor*), Jagged Fang Designs (*Compsognathus*, *Torvosaurus*, *Acrocanthosaurus*, *Daspletosaurus*), Ivan Iofrida (*Spinosaurus*), Tasman Dixon (*Dilophosaurus*, *Allosaurus*, *Yutyrannus*), and Jack Mayer Wood (*Tyrannosaurus*), Alessio Ciaffi (*Abelisaurus*), Ivan Iofrida (*Baryonyx*, *Buitreraptor*, *Gorgosaurus*), Matt Martyniuk (*Ornitholestes*, *Tarbosaurus*), T. K. Robinson (composite megaraptorid), Ashley Patch (*Qianzhousaurus*), Conty (*Tarbosaurus*), and T. Michael Keesey (*Tarbosaurus*).

**Figure S17.** Cladogram depicting the average von Mises stress (Pascals) in the ancestral condition of theropod clades, specifically the posterior jugal. The sampled posterior jugal stress averages were relatively lower than the posterior jugal. The ancestral conditions of Abelisauridae and Allosauroidea show relatively lower posterior jugal stress than Tyrannosauroidea and Megalosauroidea. The ancestral condition of Maniraptora shows the highest posterior jugal stress than the previously mentioned theropod clades. The theropod clades depicted include Ceratosauria (1), Megalosauroidea (2), Allosauroidea (3), Maniraptora (4), Compsognathidae (5), and Tyrannosauroidea (6). Images from Phylopic. Artist credit: Scott Hartman (*Coelophysis*, *Ceratosaurus*, *Majungasaurus*, *Carnotaurus*, *Giganotosaurus*, *Deinonychus*, *Monolophosaurus*, *Guanlong*, *Bistahieversor*), Jagged Fang Designs (*Compsognathus*, *Torvosaurus*, *Acrocanthosaurus*, *Daspletosaurus*), Ivan Iofrida (*Spinosaurus*), Tasman Dixon (*Dilophosaurus*, *Allosaurus*, *Yutyrannus*), and Jack Mayer Wood (*Tyrannosaurus*), Alessio Ciaffi (*Abelisaurus*), Ivan Iofrida (*Baryonyx*, *Buitreraptor*, *Gorgosaurus*), Matt Martyniuk (*Ornitholestes*, *Tarbosaurus*), T. K. Robinson (composite megaraptorid), Ashley Patch (*Qianzhousaurus*), Conty (*Tarbosaurus*), and T. Michael Keesey (*Tarbosaurus*).

**Figure S18.** Cladogram depicting the average von Mises stress (Pascals) in the ancestral condition of theropod clades, specifically the maxilla‐jugal suture. The ancestral condition of Abelisauridae shows relatively lower maxilla‐jugal stress than Megalosauroidea, Tyrannosauroidea, and Allosauroidea. The ancestral condition of Maniraptora shows the highest maxilla‐jugal stress. The theropod clades depicted include Ceratosauria (1), Megalosauroidea (2), Allosauroidea (3), Maniraptora (4), Compsognathidae (5), and Tyrannosauroidea (6). Images from Phylopic. Artist credit: Scott Hartman (*Coelophysis*, *Ceratosaurus*, *Majungasaurus*, *Carnotaurus*, *Giganotosaurus*, *Deinonychus*, *Monolophosaurus*, *Guanlong*, *Bistahieversor*), Jagged Fang Designs (*Compsognathus*, *Torvosaurus*, *Acrocanthosaurus*, *Daspletosaurus*), Ivan Iofrida (*Spinosaurus*), Tasman Dixon (*Dilophosaurus*, *Allosaurus*, *Yutyrannus*), and Jack Mayer Wood (*Tyrannosaurus*), Alessio Ciaffi (*Abelisaurus*), Ivan Iofrida (*Baryonyx*, *Buitreraptor*, *Gorgosaurus*), Matt Martyniuk (*Ornitholestes*, *Tarbosaurus*), T. K. Robinson (composite megaraptorid), Ashley Patch (*Qianzhousaurus*), Conty (*Tarbosaurus*), and T. Michael Keesey (*Tarbosaurus*).

**Figure S19.** Cladogram depicting the average von Mises stress (Pascals) in the ancestral condition of theropod clades, specifically the squamosal. The sampled squamosal stress averages were the highest compared to the other sampled cranial points. The ancestral conditions of Tyrannosauroidea and Allosauroidea show relatively lower squamosal stress averages than other theropod clades. The theropod clades depicted include Ceratosauria (1), Megalosauroidea (2), Allosauroidea (3), Maniraptora (4), Compsognathidae (5), and Tyrannosauroidea (6). Images from Phylopic. Artist credit: Scott Hartman (*Coelophysis*, *Ceratosaurus*, *Majungasaurus*, *Carnotaurus*, *Giganotosaurus*, *Deinonychus*, *Monolophosaurus*, *Guanlong*, *Bistahieversor*), Jagged Fang Designs (*Compsognathus*, *Torvosaurus*, *Acrocanthosaurus*, *Daspletosaurus*), Ivan Iofrida (*Spinosaurus*), Tasman Dixon (*Dilophosaurus*, *Allosaurus*, *Yutyrannus*), and Jack Mayer Wood (*Tyrannosaurus*), Alessio Ciaffi (*Abelisaurus*), Ivan Iofrida (*Baryonyx*, *Buitreraptor*, *Gorgosaurus*), Matt Martyniuk (*Ornitholestes*, *Tarbosaurus*), T. K. Robinson (composite megaraptorid), Ashley Patch (*Qianzhousaurus*), Conty (*Tarbosaurus*), and T. Michael Keesey (*Tarbosaurus*).

**Figure S20.** Cladogram depicting the average von Mises stress (Pascals) in the ancestral condition of theropod clades, specifically the postorbital and lacrimal suture. The ancestral conditions of Abelisauridae, Allosauroidea, Megalosauroidea, and Tyrannosauroidea show relatively lower postorbital‐lacrimal stress than Maniraptora. The theropod clades depicted include Ceratosauria (1), Megalosauroidea (2), Allosauroidea (3), Maniraptora (4), Compsognathidae (5), and Tyrannosauroidea (6). Images from Phylopic. Artist credit: Scott Hartman (*Coelophysis*, *Ceratosaurus*, *Majungasaurus*, *Carnotaurus*, *Giganotosaurus*, *Deinonychus*, *Monolophosaurus*, *Guanlong*, *Bistahieversor*), Jagged Fang Designs (*Compsognathus*, *Torvosaurus*, *Acrocanthosaurus*, *Daspletosaurus*), Ivan Iofrida (*Spinosaurus*), Tasman Dixon (*Dilophosaurus*, *Allosaurus*, *Yutyrannus*), and Jack Mayer Wood (*Tyrannosaurus*), Alessio Ciaffi (*Abelisaurus*), Ivan Iofrida (*Baryonyx*, *Buitreraptor*, *Gorgosaurus*), Matt Martyniuk (*Ornitholestes*, *Tarbosaurus*), T. K. Robinson (composite megaraptorid), Ashley Patch (*Qianzhousaurus*), Conty (*Tarbosaurus*), and T. Michael Keesey (*Tarbosaurus*).
