# Supplementary material for: Comparative cranial biomechanics reveal macroevolutionary trends in theropod dinosaurs, with emphasis on Tyrannosauroidea
Source: Anat Rec (Hoboken). 2026 Jan 27;309(11):3036–76. doi: 10.1002/ar.70126 (PMC13644910; doi:10.1002/ar.70126)
Supplement: Supplementary file 1 [file AR-309-3036-s001.html]

Comparative Analyses


# Comparative Analyses

Author

Johnson-Ransom et al

# Part 0: Load necessary R packages

load necessary R packages

```
library(ape)
library(phytools)
library(geiger)
library(tidyverse)
library(phytools)
library(phangorn)
library(strap)
library(EvoPhylo)
library(paleotree)
library(caper)
#library(RERconverge)
library(surface)
```

# Part 1: Tip Dating Analysis

Load the data and the tree

```
tree <- read.tree("./TREES/All_Theropods_FEA.tree")
data_raw <- read.csv("./DATA/Phylogenetic_Data_6.csv", row.names = 1)
colnames(data_raw) <- c("Skull_Length","vonMises","earliest","latest")
```

check that the data and tree match up.

```
nc <- name.check(phy = tree, data = data_raw)
nc
```

```
[1] "OK"
```

```
data <- data_raw
plot(tree, cex = .5)
```

We are going to use `MrBayes` to perform the tip dating analysis. We need to take the first and last occurrence dates and convert it into a different format where we tell `MrBayes` which time period each species existed in

```
Ranges <- binTimeData(data[,3:4])
rownames(Ranges$taxon.times) <- rownames(data)
```

Create and save a special nexus file that includes a block of data with instructions for `MrBayes`

an important note from Herrara-Flores et al 2021 (*Palaeontology*):

> “MrBayes ignores topological constraints at the root, if the root node is fully resolved, so we added two outgroup taxa, the lepidosauromorphs *Sophineta cracoviensis* (249.2–247.2 Ma) and *Paliguana whitei* (251.9–251.2 Ma) to a polytomy at the root including the clade Lepidosauria.”

As such, we added outgroup taxa *Tawa* and *Eodromaeus* as a polytomy at the root of the tree. Furthermore, we need to have one extant taxon to calibrate the node dates as `MrBayes` calculates node dates as time before present. We added in *Passer* as our extant tip.

We will add an additional node prior to this tree: Based on evidence from Xu Xing, et al. 2010 and Hendrickx et al 2015, we should expect the most recent common ancestor of Theropoda to be in the Carnian.

Then go to the terminal execute this code:

`mb`

`Execute "./TREES/tipdatingnexus2024.nex";`

# Part 2: Phylogenetic Comparative Analyses

When that is finished we can load in the posterior distribution of trees

```
treefile <- "./TREES/tipdatingnexus2024.nex.run1.t"
```

Probably the best thing to do is to run all the analyses on a sample of 10 or 100 trees from the post-burnin posterior. First we will get the MCCT (maximum clade credibility tree) for use in figures. this is a summary tree.

```
MCCT <- obtainDatedPosteriorTreesMrB(
    runFile = treefile,
    nRuns = 2, 
    burnin = 0.5,
    outputTrees = "MCCT",
    getFixedTimes = TRUE,
    file = NULL)

geoscalePhylo(MCCT)
```

get random trees from the posterior:

```
RandomlySelectedTrees <- obtainDatedPosteriorTreesMrB(
    runFile = treefile,
    nRuns = 2, 
    burnin = 0.5, 
    getFixedTimes = TRUE,
    getRootAges = TRUE,
    outputTrees = 400, 
    file = NULL)

maxager <- function(x){max(dateNodes(x))}

rootages<-lapply(RandomlySelectedTrees, maxager)
which(rootages < 237)
```

```
[1]  21  53  69 146 149 177 248 375
```

```
decent_trees<-RandomlySelectedTrees[which(rootages < 237)]
decent_trees
```

```
8 phylogenetic trees
```

```
#write.nexus(decent_trees,
#            file = "./TREES/tipdatingnexus2025sample.nex")
```

Drop the tips that for which we don’t have data. Also make sure we don’t have any branches that are too short.

```
trees_2 <- lapply(decent_trees, drop.tip, c("Tawa",
                                            "Eodromaeus",
                                            "Passer"))
trees_2 <- lapply(trees_2, minBranchLength, mbl = 1, modifyRootAge = FALSE)

rootages2<-sapply(decent_trees, `[[`, "root.time")

for (i in length(trees_2)){
trees_2[[i]]$root.time <- rootages2[i]
}

class(trees_2)<-"multiPhylo"

tree_MCCT <- drop.tip(MCCT, c("Tawa", "Eodromaeus", "Passer"))
tree_MCCT <- minBranchLength(tree = tree_MCCT, mbl = 1, modifyRootAge = FALSE)
```

```
geoscalePhylo(trees_2[[1]],
              boxes = "Epoch",
              units= c("Period", "Epoch"),
              x.lim = c(20,235),
              cex.age = 0.7,
              cex.ts = 0.7,
              cex.tip = 0.6,
              width = 2)
```

make sure data and tip labels are in same order:

```
data <- data[trees_2[[1]]$tip.label,]
```

first we will test the strength of phylogenetic signal, quantified using pagel’s lambda

```
vonmises<-data$vonMises
names(vonmises)<-rownames(data)

signal <- phytools::phylosig(tree = trees_2[[1]],
                   x = vonmises,
                   method = "lambda")
signal
```

```
Phylogenetic signal lambda : 0.0781174 
logL(lambda) : -3.48313
```

and repeat with the distribution of trees

```
sig7 <- lapply(trees_2, phytools::phylosig,
                x = data$vonMises,
                method = "lambda")
```

```
[1] "x has no names; assuming x is in the same order as tree$tip.label"
[1] "x has no names; assuming x is in the same order as tree$tip.label"
[1] "x has no names; assuming x is in the same order as tree$tip.label"
[1] "x has no names; assuming x is in the same order as tree$tip.label"
[1] "x has no names; assuming x is in the same order as tree$tip.label"
[1] "x has no names; assuming x is in the same order as tree$tip.label"
[1] "x has no names; assuming x is in the same order as tree$tip.label"
[1] "x has no names; assuming x is in the same order as tree$tip.label"
```

```
lambda7 <- sapply(sig7, `[[`, "lambda")
meanlambda <- mean(lambda7)
sdlambda <- sd(lambda7)

print(paste0("Mean phylogenetic signal (lambda) is ", meanlambda))
```

```
[1] "Mean phylogenetic signal (lambda) is 0.0558530092102263"
```

```
print(paste0("Standard deviation of phylogenetic signal (lambda) is ",
             sdlambda))
```

```
[1] "Standard deviation of phylogenetic signal (lambda) is 0.0491187593467261"
```

A couple of plotting options:

```
vonM <- setNames(data$vonMises,
    rownames(data))
phenogram(tree = trees_2[[1]], x=vonM)
```

```
Optimizing the positions of the tip labels...
```

Another way of visualizing this

```
obj<-phytools::contMap(trees_2[[1]],vonM,plot=FALSE)
obj<-phytools::setMap(obj,invert=TRUE)
#change colors to more colorblind friendly
n<-length(obj$cols)
obj$cols[1:n] <- viridis::viridis(n=n, option = "turbo")
plot(obj,fsize=c(0.8,1),outline=TRUE,lwd=c(3,7),leg.txt="log(von Mises Stress)")
```

next we will test the strength of allometry using pgls

```
data <- data %>% mutate(Species = rownames(data))
trees_2[[1]]$node.label <- c(1:length(trees_2[[1]]$node.label))
comp_dat <- comparative.data(phy = trees_2[[1]],
                             data = data,
                             names.col = Species)
fit1 <- pgls(formula = vonMises ~ Skull_Length,
             data= comp_dat,
             lambda = signal$lambda)
S1 <- summary(fit1)
S1
```

```
Call:
pgls(formula = vonMises ~ Skull_Length, data = comp_dat, lambda = signal$lambda)

Residuals:
      Min        1Q    Median        3Q       Max 
-0.097004 -0.007523  0.001895  0.008269  0.117968 

Branch length transformations:

kappa  [Fix]  : 1.000
lambda [Fix]  : 0.078
delta  [Fix]  : 1.000

Coefficients:
              Estimate Std. Error t value Pr(>|t|)    
(Intercept)   6.257418   0.185702 33.6959   <2e-16 ***
Skull_Length -0.093844   0.109231 -0.8591   0.3953    
---
Signif. codes:  0 '***' 0.001 '**' 0.01 '*' 0.05 '.' 0.1 ' ' 1

Residual standard error: 0.02635 on 41 degrees of freedom
Multiple R-squared: 0.01768,    Adjusted R-squared: -0.006275 
F-statistic: 0.7381 on 1 and 41 DF,  p-value: 0.3953
```

```
pvals<-c()
for ( i in 1:length(trees_2)){
  comp_dat <- comparative.data(phy = trees_2[[i]],
                             data = data,
                             names.col = Species)
  fitA <- pgls(formula = vonMises ~ Skull_Length,
             lambda = lambda7[i],
             data= comp_dat)
SA<-summary(fitA)
pvals[i] <- SA$coefficients[2,4]
}
pvals
```

```
[1] 0.3952636 0.3917468 0.3947871 0.3802219 0.4096974 0.3819668 0.3808923
[8] 0.3971260
```

```
min(pvals)
```

```
[1] 0.3802219
```

```
max(pvals)
```

```
[1] 0.4096974
```

**RESULT:** no significant correlation between skull length and vonMises stress. This result is robust to differences in tip dating. below is a messy plot showing that with the exception of *Buitreraptor* and *Guanlong*, there is very little variation in stress across skull sizes (the regression line is flat)

```
xx <- ggplot(comp_dat$data, aes(x=Skull_Length, 
                                y=vonMises,
                                label = rownames(comp_dat$data)))+
  geom_point()+
  geom_abline(intercept = coef(fit1)[1], slope = coef(fit1)[2])+
  geom_text()+
  theme_minimal()+
  xlab("log10(Skull Length (cm))")+
  ylab("log10(Average von Mises Stress (Pa))")
xx
```

# Part 3: Subsets of the data

It is now very clear that *Buitreraptor gonzalezorum* and *Guanlong wucaii* are outliers. We will now remove it and re-run the analyses

```
trees_trimmed<-drop.tip(trees_2, c("Buitreraptor_gonzalezorum","Guanlong_wucaii"))

for (i in 1:length(trees_trimmed)){
  trees_trimmed[[i]]$node.label <- NULL
  }
vonM_trimmed<-vonM[-which(names(vonM) %in% c("Buitreraptor_gonzalezorum","Guanlong_wucaii"))]

obj2<-phytools::contMap(trees_trimmed[[1]],vonM_trimmed,plot=FALSE)
obj2<-phytools::setMap(obj2,invert=TRUE)
#change colors to more colorblind friendly
n2<-length(obj2$cols)
obj2$cols[1:n] <- viridis::viridis(n=n2, option = "turbo")
plot(obj2,fsize=c(0.8,1),outline=TRUE,lwd=c(3,7),leg.txt="log10(von Mises Stress)")
```

```
pvals<-c()
data_trimmed<-data
data_trimmed <- data_trimmed[-which(data_trimmed$Species %in% c("Buitreraptor_gonzalezorum","Guanlong_wucaii")),]
for ( i in 1:length(trees_trimmed)){
  comp_dat <- comparative.data(phy = trees_trimmed[[i]],
                             data = data_trimmed,
                             names.col = Species)
  fitA <- pgls(formula = vonMises ~ Skull_Length,
             lambda = lambda7[i],
             data= comp_dat)
SA<-summary(fitA)
pvals[i] <- SA$coefficients[2,4]
}
pvals
```

```
[1] 0.2303348 0.2405036 0.2291725 0.2698492 0.2135790 0.2681726 0.2689057
[8] 0.2268178
```

```
min(pvals)
```

```
[1] 0.213579
```

```
max(pvals)
```

```
[1] 0.2698492
```

Removing the outliers doesn’t change much. There is still no good relationship between size and stress.

We also through it might be good to look at patterns within specific subclades

```
tyrannosauridae <- extract.clade(trees_2[[1]],
                                 getMRCA(phy = trees_2[[1]],
                                   c("Guanlong_wucaii",
                                     "Tyrannosaurus_rex")))
T.vonM <- vonM[which(names(vonM) %in% tyrannosauridae$tip.label)]


obj2<-phytools::contMap(tyrannosauridae,T.vonM,plot=FALSE,
                        lims = c(min(vonM),max(vonM)))
obj2<-phytools::setMap(obj2,invert=TRUE)
#change colors to more colorblind friendly
n<-length(obj2$cols)
obj2$cols[1:n] <- viridis::viridis(n=n, option = "turbo")
plot(obj2,fsize=c(0.4,1),outline=TRUE,lwd=c(3,7),leg.txt="log10(von Mises Stress)")
```

```
abelisauridae<- extract.clade(trees_2[[1]],
                                 getMRCA(phy = trees_2[[1]],
                                   c("Masiakasaurus_knopfleri",
                                     "Carnotaurus_sastrei")))
#data$clade[which(data$Species %in% abelisauridae$tip.label)] <- 1
```

# Part 4: Detecting Adaptive Optima

Now we will test convergence with the `surface` package and plot the results. This method fits an OU model that automatically detects the number of adaptive optima. I would predict that maybe the *Guanlong* and *Buitreraptor* have each evolved toward unique adaptive optima and the remaining taxa share a common adaptive optimum

```
data2 <- data 

data2 <- as.data.frame(data2)
trees_2[[1]]$node.label<-NULL
trees_2[[1]]<-nameNodes(trees_2[[1]])
result<-runSurface(trees_2[[1]],data2[2])

cbbPalette <- c("#000000", "#E69F00", "#56B4E9",
                "#009E73", "#F0E442", "#0072B2",
                "#D55E00", "#CC79A7")

surfaceTreePlot(trees_2[[1]],
                result$bwd[[1]],
                labelshifts = F,
                cex=.5,
                cols = cbbPalette)
```
